# Repeated polyploidization shapes divergence in floral morphology in *Lithophragma bolanderi* (Saxifragaceae)

**DOI:** 10.1073/pnas.2505119122

**Published:** 2025-08-13

**Authors:** Karin Gross, Homa Papoli Yazdi, Elisabeth Schlager, Jodie Lilley, Andrés Romero-Bravo, Anna Runemark, John N. Thompson, Magne Friberg

**Affiliations:** ^a^Department of Plant Ecology and Evolution, Evolutionary Biology Centre, Uppsala University, Norbyvägen, Uppsala 18D, 752 36, Sweden; ^b^Department of Biology, Lund University, Lund 223 62, Sweden; ^c^Department of Environment and Biodiversity, Paris Lodron University of Salzburg, Salzburg 5020, Austria; ^d^Faculty of Biology, Medicine and Health, University of Manchester, Manchester M13 9PL, United Kingdom; ^e^Department of Ecology and Evolution, School of Life Sciences, University of Sussex, Brighton BN1 9RH, United Kingdom; ^f^Department of Ecology and Evolutionary Biology, University of California, Santa Cruz, CA 95060

**Keywords:** polyploidy, diversification, floral evolution, geographic mosaic, pollination

## Abstract

Polyploidization is an important driver of evolution and diversification in flowering plants. Here, we assess how repeated polyploidization may have shaped diversification of floral morphology in *Lithophragma bolanderi* (Saxifragaceae). This species comprises multiple cytotypes and varies geographically in its interactions with specialized pollinating moths in the genus *Greya* (Prodoxidae). Past studies have shown that coevolution with these moths has favored particular suites of floral characters but does not fully explain local and regional floral diversification. We combined phenotypic and genomic data from more than 1,800 individuals from 40 *L. bolanderi* populations spread across its entire range. Flow-cytometric analyses revealed a geographic mosaic of populations comprising one to four of three dominant (diploid, tetraploid, hexaploid) and three rare (triploid, pentaploid, octoploid) cytotypes. Whole-genome resequencing of a subset of populations suggested that polyploids arose from multiple autopolyploidization events, rather than a single event and/or through hybridization, albeit with some signals consistent with low levels of introgression from the congener *Lithophragma glabrum*. Quantification of flower traits from plants grown in a common garden showed that cytotype explained more than 15% of the variation in floral morphology, with polyploids showing more variability than diploids. Experimental induction of neopolyploids directly induced phenotypic changes but also indicated that local selection may have favored subsequent convergence in floral morphology among cytotypes in natural populations. Collectively, this comprehensive and integrative approach provides insights into how variability generating processes, such as polyploidization integrates with selection from species interactions to shape local floral diversification.

## Significance

Polyploidization and coevolution with animals are two of the major drivers of plant diversification through their effect on ecologically important traits. Past studies have shown that coevolution with pollinating moths has shaped floral diversification in *Lithophragma bolanderi* (Saxifragaceae), but coevolution alone is insufficient to fully explain the current floral variation. We assessed how repeated polyploidization may have contributed to floral diversification. Ploidy varied from diploid to octoploid across the range of this species. Field and common garden studies together with experimental studies of newly formed polyploids suggest that repeated polyploidization contributed to the regional diversification of floral characters. Our results provide empirical insights into how polyploidization may contribute to the formation of geographic mosaics of coevolving interactions.

Polyploidy, the presence of more than two chromosome sets per cell, is associated with an increase in species richness in multiple angiosperm families (1–3) and is considered a major driver of plant diversification (3–7). Polyploidy can arise through autopolyploidization within species or as allopolyploidization involving hybridization (e.g., 8, 9). Strong postzygotic reproductive isolation among cytotypes (8) and direct effects of polyploidization on plant phenotypes (e.g., 10–14) may allow different cytotypes to immediately evolve along different evolutionary trajectories (14–17) and facilitate polyploids to establish into novel ecological niches (10). Divergent selection acting on the incipient species (18, 19) may drive among-cytotype niche differentiation, facilitating coexistence. Past studies have shown that polyploidization can affect patterns of attack by herbivores and visitation by pollinators (e.g., 20–27), which may lead to differential selection pressures in polyploids compared to diploids.

Polyploids may differ from diploid ancestors in ecologically important traits, including those involved in plant–pollinator interactions, such as floral size (10, 14, 15, 21, 28–31) and shape (15, 32). The direction and magnitude of such differences are, however, inconsistent among study systems (28) and among independent polyploidization events involving the same parental lineages (11, 15–17). In addition, an increased or fixed heterozygosity in polyploids, genomic instability, neo- or subfunctionalization of genes, altered gene expression, and/or multiple independent polyploidization events could increase trait variation (11, 33–38), whereas the involvement of few founding individuals in polyploidization events could bottleneck variation in polyploids (16).One major recent challenge for evolutionary biologists and plant breeders is to identify a polyploidy paradigm (3, 39) that allows predictions of phenotypic and genetic responses to polyploidization across plant lineages and how this integrates into the plants’ interaction with pollinators. However, such predictions have proven difficult, because most studies investigating the impact of polyploidy on floral morphology include only few populations and/or samples (but see 15, 40, 41). Limited ecological replicas impose a risk to overlook cytotypes and/or polyploidization events, to underestimate the extent of trait variation in a species, and to hamper our understanding of the impact of selective agents on phenotypic traits affected by polyploidization (but see 18, 19, 42).

A first step to allow predictions across plant lineages are studies that obtain more complete insights into natural model systems (3). Such studies should ideally include i) assessments of the polyploidization mode (i.e., auto- vs. allopolyploidization), ii) comparisons among sympatric and allopatric populations of different cytotypes, and iii) comparisons of natural trait variation among cytotypes with experimentally generated neopolyploids, and should focus on iv) species in which the selective agents and phenotypic targets of selection are established. Here, we utilize one such system, the plant *Lithophragma bolanderi* (Saxifragaceae), to test several hypotheses about the impact of polyploidy on floral trait diversification and how these effects integrate with the selection imposed by pollinators.

Polyploidy is particularly common in the plant family Saxifragaceae (2, 43). In the genus *Lithophragma* in general and in the species *L. bolanderi* in particular, chromosome counts have indicated considerable ploidy level variation (44), and this species also shows ample among-population variation in morphological (45) and chemical (46) floral traits. *Lithophragma bolanderi* is the only *Lithophragma* species with three documented even-numbered cytotypes (44)—diploids (2×, 2n = 14), tetraploids (4×, 2n = 28), and hexaploids (6×, 2n = 42). Analyses of internal transcribed spacer (ITS) regions of nuclear, ribosomal DNA (ITS) sequences suggested a possible allotetraploid origin, because the same tetraploid individuals clustered with both *L. bolanderi* and *L. glabrum* for different ITS sequence variants (47). However, this initial interpretation was based on few populations and one genetic marker, which prevented a full evaluation of the history and origin of polyploidy in *L. bolanderi* until broader sampling could be undertaken and multigene genomic tools were developed and refined.

*L. bolanderi*, along with several congeners, is coevolving with two *Greya* moth species (48–50), *Greya politella* and *Greya obscura* (Prodoxidae), that completely depend on *Lithophragma* as host- and nectar plants. *G. politella* pollinates *Lithophragma* flowers highly efficiently during oviposition into the floral ovary and also during nectaring, whereas *G. obscura* oviposits into nonreproductive floral and stem tissue and pollinates, less efficiently, only during nectaring (51–53). Two lines of recent evidence indicate how these *Greya* moths impose local selection on floral morphology. First, a genus-wide study showed evolutionary divergence in combinations of floral morphological traits both among *Lithophragma* species and among *L. bolanderi* populations, depending on whether they co-occur with only *G. politella*, only *G. obscura*, or both species (45). Second, two morphologically divergent *L. bolanderi* populations were most effectively pollinated by their local *Greya* moths (53).

Although these coevolving interactions between *Lithophragma* and *Greya* have contributed to the evolution and diversification of floral morphology, there remains much unexplained geographic and local variation in floral morphology. Collectively, the ample floral trait variation (45), the established selection agents (45, 53), and the probable multiple polyploidization events (44) primes *L. bolanderi* as an ideal species in which to investigate the evolutionary dynamics of polyploidy, its role as a driver of floral-trait evolution, and how this integrates with the already established contribution of pollinators in floral trait divergence.

Here, we assess the full extent of the number and geographic distribution of cytotypes within *L. bolanderi*, the evolution of polyploidy through auto- or allopolyploidization, and the extent to which divergence in floral morphology could reflect an immediate result of polyploidization. We used a combination of field collections and a common-garden approach, together comprising a total of 1802 *L. bolanderi* individuals from 40 populations covering the entire species range, genomic analyses, and experimentally induced polyploidization. We used flow cytometry to establish the number of cytotypes and their geographical distribution. We produced a reference genome and conducted whole-genome resequencing of a subset of the populations to assess population genetic structure and to determine the minimum number and nature (auto- and/or allopolyploidization) of the polyploidization events. We measured a set of floral morphological traits from all common garden individuals to partition the variance explained by cytotype, and to compare the extent of among-cytotype differences across populations with those in sympatry. For one population with diploid and tetraploid plants, we applied a colchicine treatment to generate neopolyploids, which allowed comparisons of immediate changes in floral morphology induced by polyploidization with the floral morphology of natural cytotypes. Collectively, these approaches allow us to integrate effects of polyploidization with effects of *Greya*-moth-imposed selection, which has previously been quantified (45), on local divergence in floral morphology in *L. bolanderi*.

## Results

### Cytotypes in *L. bolanderi* and their Geographical Distribution.

**Fig. 1.**
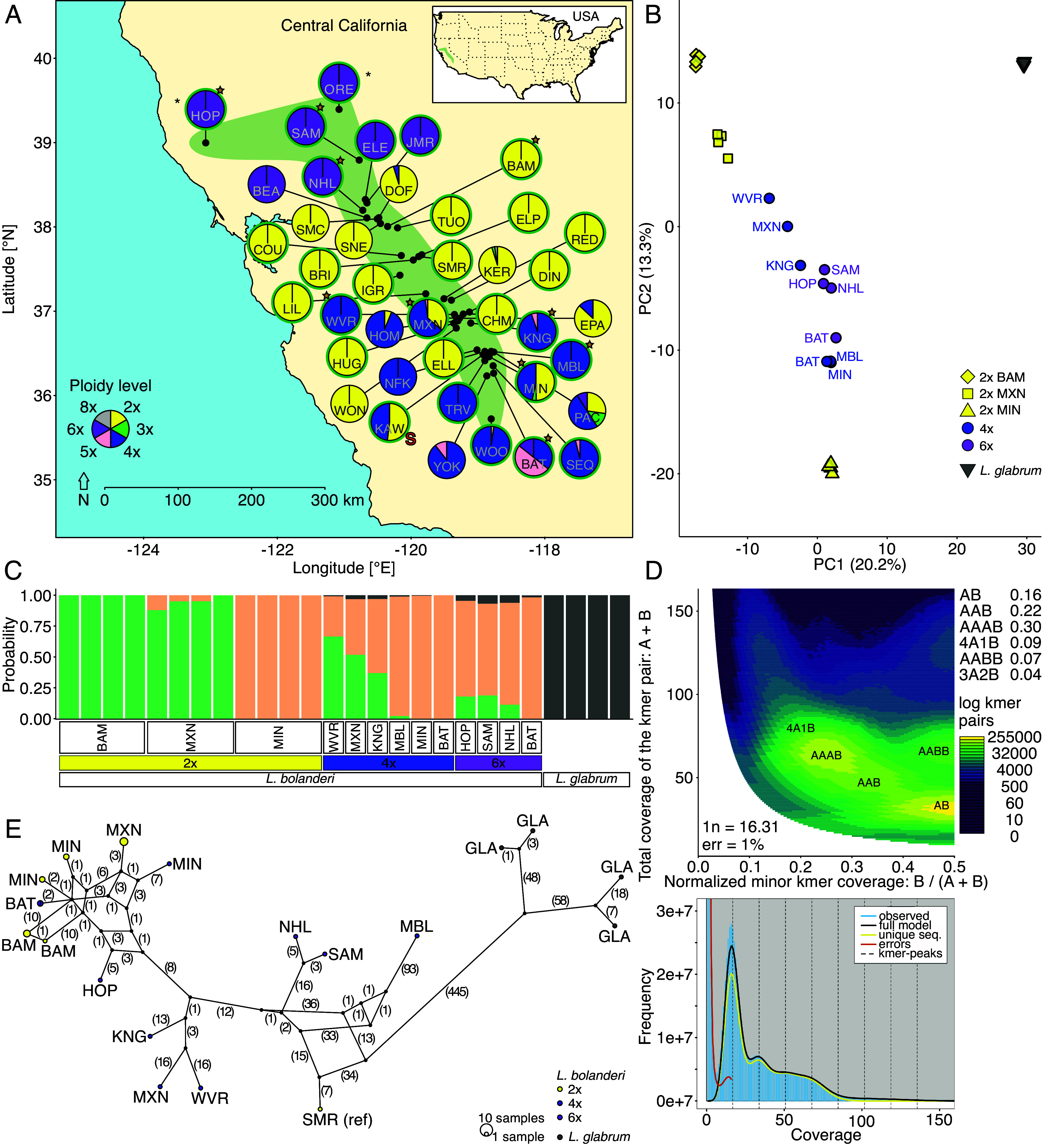
Geographical distribution of three dominant (2×: diploids, 4×: tetraploids, 6×: hexaploids) and three rare (3×: triploids, 5×: pentaploids, 8×: octoploids) cytotypes across the distribution range of *Lithophragma bolanderi* (*A*) and genetic relationships among selected individuals of polyploid *L. bolanderi* as well as of individuals from the parental lineages of polyploid *L. bolanderi* suggested by Kuzoff et al. (47)—diploid *L. bolanderi* and *L. glabrum*—based on the whole nuclear genome (*B*–*D*) and based on the whole chloroplast genome (*E*). (*A*) Each pie diagram represents a population and shows the relative abundance of each cytotype. The three letter codes within the pie diagrams indicate the population’s abbreviation and “*” next to the two northernmost populations indicates very low sample sizes (for details on populations and the sample sizes, see *SI Appendix*, Table S1). Pie diagrams framed in green indicate populations that were grown in the common garden, pie diagrams with a brown star indicate populations from which plants were used for whole-genome resequencing, and the pie diagram with a red “S” indicates the population from which plants were used for the synthetic polyploidization experiment. The approximate species’ distribution range is indicated by the green area both in the main map of central California and in the insert map of the United States on the *Top Right*. (*B*) Illustration by a principal component analysis of genetic structure based on 21,667 linkage-pruned single nucleotide polymorphisms (SNPs) shown as principal component (PC) scores. Symbols represent individuals color-coded according to cytotype and species. Population identity is provided, and the variance explained by the PCs is given in brackets. Note that all four *L. glabrum* individuals cluster very closely together. (*C*) Structure plot based on 21,667 SNPs clustering individuals into three clusters (K = 3) in STRUCTURE. Bars represent individuals with the proportion of the color corresponding to the likelihood of the individual belonging to that cluster, and cytotype and species identity are provided. Populations within cytotypes are ordered according to latitude from north (*Left*) to south (*Right*). (*D*) Smudgeplot (*Top*) and k-mer spectrum plot (*Bottom*) for the tetraploid *L. bolanderi* individual from the MBL population, which is representative of all tetraploids. (*E*) Haplotype network of cpDNA haplotypes, and the number of mutation steps are given in parentheses. Individuals/populations are color-coded according to cytotype and species. Note that the tetraploid and hexaploid individuals from BAT have the same haplotype.

The cytotype of 1045 *L. bolanderi* individuals from 40 populations was determined using flow cytometry. Among those individuals, 786 were from 501 seed families collected in 29 natural populations and grown in a common garden, and the remaining 249 were field-collected samples from 11 populations (*SI Appendix*, Table S1). We detected six clearly defined ploidy groups based on sample:internal-standard (IS) peak ratios (*SI Appendix*, Fig. S1 *A* and *B*), which match the cytotypes found by Taylor (44) using karyotypic analysis. They represent three dominant cytotypes (cf. 54—diploids (2×; 48% of samples), tetraploids (4×; 38%), and hexaploids (6×; 11%)—and three rare cytotypes—triploids (3×; 1%), pentaploids (5×; 2%), and octoploids (8×; 0.1%), of which triploids and pentaploids were most likely hybrids between two dominant cytotypes or their backcrosses into parental lineages (*SI Appendix*, Fig. S1 *A* and *B*). The distribution of cytotypes was geographically structured with hexaploids occurring mainly in the north, diploids mainly in the center, and tetraploids mainly in the south of the distribution (Fig. 1*A*). The geographical transition from hexaploid to diploid populations in the north was sharp, whereas in the south, pure diploid, pure tetraploid, and several mixed-ploidy populations occurred in a mosaic. Overall, 67.5% of the populations (n = 27) comprised a single cytotype and 32.5% (n = 13) two to four cytotypes. Diploids and hexaploids were more likely to occur in pure populations than tetraploids (*SI Appendix*, Fig. S2).

### Polyploidization Mode and Number of Polyploidization Events.

*Lithophragma glabrum* was identified as a potential parental lineage to polyploid *L. bolanderi* by Kuzoff et al. (47) as tetraploid *L. bolanderi* had both *L. glabrum* and diploid *L. bolanderi* ITS variants. Our analyses of the ITS sequences confirmed the patterns detected by Kuzoff et al. (47) (*SI Appendix*, Fig. S3 and Table S2). We further unraveled the history of polyploidization in *L. bolanderi* by leveraging whole-genome resequencing. We mapped 12 diploid individuals (four each from three populations), six tetraploid individuals (one each from six populations), and four hexaploid individuals (one each from four populations) for *L. bolanderi* (mapping rates: 97.14 to 97.46%; *SI Appendix*, Table S3), and four individuals from one population for *L. glabrum* (93.39 to 95.13%; *SI Appendix*, Table S3) to our *L. bolanderi* reference genome, which was generated based on PacBio-sequencing of one diploid individual from the SMR population (for details, see Methods; *SI Appendix*, Supporting Text S3.2 and Table S1). Collectively, our whole-genome analyses are consistent with the patterns expected under autopolyploidization (Fig. 1 *B*–*E* and *SI Appendix*, Table S4). First, analyses of mode of polyploidy based on k-mer frequencies support autopolyploid origin as the most parsimonious explanation for the data (55) (Fig. 1*D* and *SI Appendix*, Table S5). Second, allele frequency spectra of tetraploids and hexaploids divided into separate groups, lacked the peaks at intermediate allele frequencies expected in allopolyploids (56) (*SI Appendix*, Fig. S4). Further, SNPs fixed for different alleles in *L. bolanderi* and *L. glabrum* had a strong overrepresentation of *L. bolanderi* alleles (~90% in 4× and ~80% in 6×) across all polyploids (*SI Appendix*, Fig. S5). Population genomic analyses, including principal component analysis (PCA) (Fig. 1*B* and *SI Appendix*, Table S6), cluster analysis in STRUCTURE (57) (Fig. 1*C*), and phylogenetic analyses performed in TreeMix (58, 59) (*SI Appendix*, Fig. S6) clearly separated out *L. glabrum* as divergent from all *L. bolanderi* populations. However, the most parsimonious TreeMix phylogenetic tree (*SI Appendix*, Fig. S6) included five migration edges, of which two suggested some introgression from *L. glabrum* into the tetraploid individual from the KNG population and the hexaploid individual from the BAT population. Formal tests implemented in GRAMPA (60) identified *L. glabrum* as outgroup to all *L. bolanderi* individuals in 91% of the recovered trees, including two of the three most strongly supported trees (*SI Appendix*, Fig. S7 *A* and *C*). Nevertheless, in the third tree, *L. glabrum* was a sister species to one of the two major *L. bolanderi* branches, including both diploids and polyploids (*SI Appendix*, Fig. S7*B*) consistent with a reticulate but not allopolyploid history. Finally, the chloroplast network indicates a deep divide between all *L. bolanderi* (diploid and polyploid) and *L. glabrum* individuals (Fig. 1*E*).

Both our whole-genome and chloroplast analyses supported several independent origins of polyploids. The hexaploid individual from the BAT population clustered closely with the tetraploid individual from this population and the southern diploids, but not the other hexaploids, in several analyses (e.g., Fig. 1 *B* and *C*). The tetraploid and hexaploid individuals from the BAT population also had identical chloroplasts (Fig. 1*E*). Furthermore, the tetraploid individuals largely fell as expected based on the geographical clustering of diploids in the PCA (Fig. 1*B*) and best GRAMPA trees (*SI Appendix*, Fig. S7), consistent with the group assignments in the STRUCTURE analysis (Fig. 1*C*). However, the hexaploid individuals from the NHL, SAM, and HOP populations fell outside of the southern and northern diploid clades in one of the three best GRAMPA trees (*SI Appendix*, Fig. S7*B*). Consistent with multiple polyploidization events, the chloroplast network analysis uncovered population structure, with tetraploids from the MIN and BAT populations and hexaploids from the BAT and HOP populations clustering with three of the four diploid *L. bolanderi* populations. Chloroplasts of tetraploids from the KNG, MXN, and WVR populations formed a separate cluster, and those of tetraploids from the MBL population and hexaploids from the NHL and SAM populations cluster with the chloroplast of the diploid individual from the SMR population (Fig. 1*E*). Finally, the identical support for three different trees in the GRAMPA analyses was consistent with a more complex, reticulate, but not allopolyploid, evolutionary history (*SI Appendix*, Fig. S7). Taken together, the molecular analyses indicate several independently evolving autopolyploid lineages in *L. bolanderi* (*SI Appendix*, Table S4).

### Cytotype, Geography, Moth-Pollinator Communities, and Floral Morphology.

We analyzed the effects of ploidy level on floral morphology using four different approaches. First, we utilized the entire dataset including floral morphological analyses of 1,457 common-garden individuals from 479 seed families and 27 populations to evaluate the presence of a “polyploidization syndrome”, that is consistent variation in floral traits among diploids, tetraploids, and hexaploids across the *L. bolanderi* distribution. Second, we kept geography constant and compared only sites where different cytotypes grew intermixed. Third, we used a subset of the dataset, including only populations where we knew the presence or absence of the two major *L. bolanderi* pollinators, the prodoxid moths *G. politella* and *G. obscura*, to directly compare effects of ploidy-level variation and pollinator-mediated selection for explaining the geographic variation in floral morphology. Finally, we generated neotetraploids to assess direct effects of polyploidization on floral morphology by comparing them with natural diploids and tetraploids from a mixed-ploidy site.

**Fig. 2.**
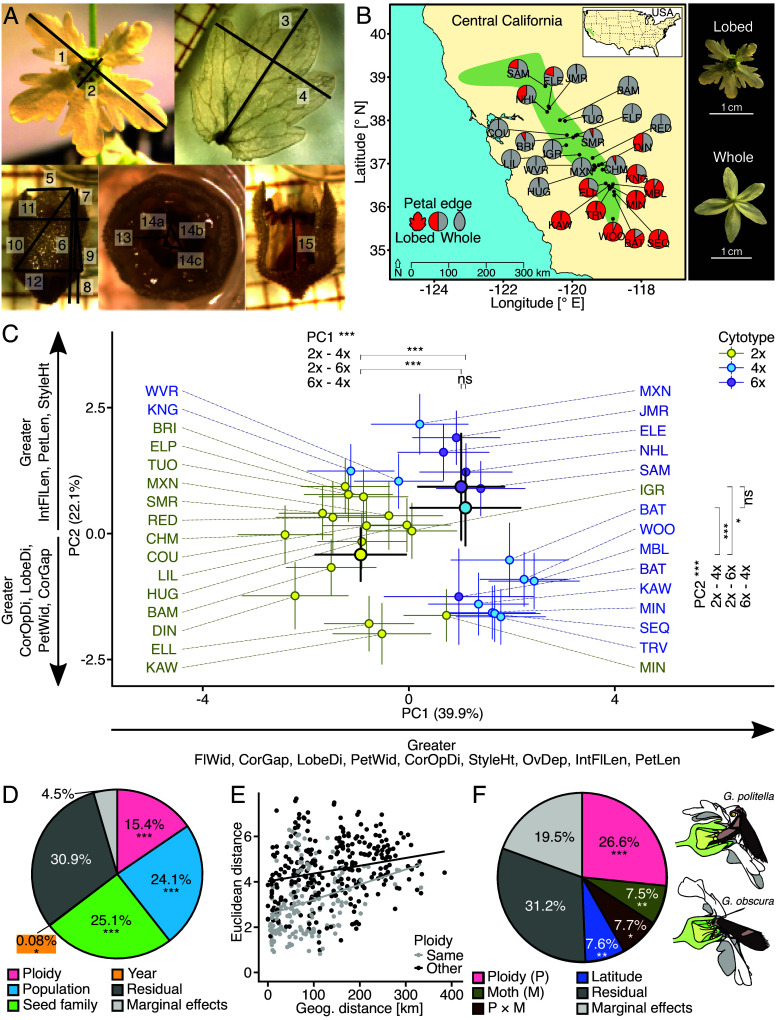
Morphological traits measured from *L. bolanderi* flowers collected in the common garden and the relationship between floral morphology, cytotype, geography, and *Greya* moth community. (*A*) For each flower, 15 floral morphological traits were quantified: (1) flower diameter (FlDi), (2) corolla-opening diameter (CorOpDi), (3) petal length (PetLen), (4) petal width (PetWid), (5) corolla gap (CorGap), (6) overall flower length (FlLen), (7) internal flower length (IntFlLen), (8) ovary depth (OvDep), (9) floral flair (FlFlair), (10) long floral angle (LgAng), (11) floral width (FlWid), (12) nectary disc length (NectLen), (13) diameter of the largest stigmatic lobe (LobeDi), (14) outer distances between lobes (LobeC), and (15) pistil height above the nectary disk (StyleHt) (for details on the measurements, see *SI Appendix*, Supporting Text S4 and Table S4). (*B*) Geographical variation in the shape of petal edge, which is either lobed or whole and a representative example of a flower with lobed petal edges (*Upper* photo; from MIN) and of a flower with whole petal edges (lower photo; from ELP). Each pie diagram on the map represents a population (three letter codes indicate the population’s abbreviation; for details, see Fig. 1*A* and *SI Appendix*, Table S1) and shows the proportion of individual with lobed and individuals with whole petal edges. (*C*) Differentiation in floral morphology among the three dominant cytotypes in *L. bolanderi* according to principal component 1 (PC1) and PC2. Smaller points and colored error bars represent population mean PC scores and *Upper* and *Lower* 95% confidence levels, respectively, and are color-coded according to cytotype. Populations are labeled (for details on populations, see Fig. 1*A* and *SI Appendix*, Table S1). Larger points with black borders and black error bars represent cytotype means and *Upper* and *Lower* 95% confidence levels, respectively. The variance explained by the PCs is given in brackets and a general interpretation of the PCs is given alongside the axes (for details on the loadings, see *SI Appendix*, Table S5 and Fig. S8). Significance levels of the linear mixed-effect models (LMMs) and of the pairwise post hoc comparisons among cytotypes are indicated on *Top* of the graph for PC1 and on the *Right* side of the graph for PC2: “ns” *P* > 0.5, *P < 0.05, ***P < 0.001. (*D*) The percentage of variance in floral morphology explained by the factors: cytotype (Ploidy), population, seed family, and year when the plants were grown (Year) estimated from permutational analyses of variance (PERMANOVA). Significance levels for these factors are indicated: *P < 0.05, ***P < 0.001. (*E*) Relationship between floral morphology similarity (Euclidean distances) and geographical distances among *L. bolanderi* populations depending on whether populations were of the same (Same ploidy) or of different (Other ploidy) cytotype. (*F*) The percentage of variance in floral morphology explained by the factors: cytotype (Ploidy), *Greya* moth community (Moth), and cytotype × moth-community interaction (P × M), as well as the covariate latitude estimated from a PERMANOVA based on population means. Significance levels are indicated: *P < 0.05, **P < 0.01, ***P < 0.001. To the *Right* of the pie chart, the two *Greya* moths interacting with *L. bolanderi* are shown represented by an ovipositing female for *G. politella* and a nectaring adult for *G. obscura*.

In the full dataset, the 15 continuously varying floral traits (Fig. 2*A* and *SI Appendix*, Table S7) typically showed larger trait values in higher ploidy levels, although both tetraploids and hexaploids differed more from diploids than between each other. Thirteen traits were significantly larger and one smaller in tetraploids than in diploids, ten were larger in hexaploids than in diploids, and five were larger and two smaller in hexaploids than in tetraploids (*SI Appendix*, Fig. S8*A*). The categorical floral trait petal-edge-shape was highly variable among and within populations (Fig. 2*B*) but did not differ significantly among cytotypes (*SI Appendix*, Fig. S8*B*).

At a multivariate level, a PCA including the nine floral traits with all pairwise correlations <0.7 (*SI Appendix*, Fig. S9 and Table S8) showed cytotype-specific clustering of floral size (PC1) and shape (PC2) (Fig. 2*C*). Both tetraploids and hexaploids had larger flowers, indicated by higher PC1 scores, and smaller corolla openings and more elongated flowers, indicated by higher PC2 scores, than diploids, but did not differ between each other (Fig. 2*C*).

In addition to average floral morphology (Fig. 2*D*), its variation, measured as multivariate dispersion, differed among cytotypes (*SI Appendix*, Table S9). Variation in morphology was lowest in diploids, intermediate in hexaploids, and largest in tetraploids. Overall, cytotype explained 15.4% of the total variation in multivariate floral morphology, and also population (24.1%) and seed family (25.1%) explained considerable amounts of the variation, whereas the year when plants were grown (<0.1%) had a negligible effect (Fig. 2*D* and *SI Appendix*, Fig. S10).

Populations growing closer together were more similar in floral morphology than more widely separated populations (partial Mantel test: *r* = 0.25, *P* = 0.001; *n*_2× populations_ = 16, *n*_4× populations_ = 10, *n*_6× populations_ = 5; Fig. 2*E*). In addition, floral morphology was more similar among populations of the same cytotype than among populations of different cytotypes (partial Mantel test: *r* = 0.38, *P* < 0.001; Fig. 2*E*). Thus, some differences in floral morphology among cytotypes were independent of geographical distances, but the higher similarity of populations growing in closer vicinity, independent of ploidy, indicates that other processes, such as local ecological selection, shape floral morphology.

One way to disentangle effects of local selection and polyploidy on floral morphology is to compare different cytotypes growing sympatrically, thereby keeping geography constant. We identified three diploid-tetraploid mixed populations (KAW, MIN, and MXN) and one tetraploid-hexaploid mixed population (BAT) (Fig. 1*A*). The extent and direction of among-cytotype differences in morphology varied among the three diploid-tetraploid mixed populations (*SI Appendix*, Table S10). Floral traits were, when significantly different, larger in tetraploids except for corolla opening diameter, which was smaller in tetraploids in MXN (*SI Appendix*, Fig. S11*A*). Three traits (flower length, long floral angle, style height) significantly differed between cytotypes in all three diploid-tetraploid mixed populations, whereas no floral trait differed significantly between tetraploids and hexaploids in BAT. In multivariate space, cytotypes were significantly diverged in PC1 and/or PC2 in all three diploid-tetraploid mixed populations but not in the tetraploid-hexaploid mixed population (*SI Appendix*, Figs. S10 and S11*B* and Table S10). In addition, multivariate floral morphology variation was larger in tetraploids than in diploids in MXN and larger in hexaploids than in tetraploids in BAT but did not differ between cytotypes in KAW and MIN (PERMDISPs; *SI Appendix*, Table S11 and Fig. S10). Overall, cytotype explained between 5.81% and 19.92% of the total floral morphology variation (PERMANOVAs; *SI Appendix*, Fig. S12). Together, these results indicate that floral morphology varied with cytotype in similar ways as across all populations but that the extent of these differences varied among sympatric sites.

For a subset of 24 populations, data were available on the presence of *Greya* moth pollinators (45, 46) (*SI Appendix*, Table S1). Fourteen of these interacted with *G. politella* only and 10 with both *G. politella* and *G. obscura*. In this dataset, 12 out of 15 continuously varying traits differed significantly among cytotypes, generally being larger in polyploids, whereas three floral traits were smaller and four larger in populations with *G. politella* only than in populations with both *Greya* species (*SI Appendix*, Fig. S13). Six traits differed both among cytotypes and between populations with different *Greya*-moth communities, and the cytotype × moth-community interaction was significant for the three traits: corolla gap, ovary depth, and stigmatic lobe diameter (*SI Appendix*, Fig. S13).

Like in the full dataset, both PC1- and PC2-scores were higher in polyploids than in diploids, whereas only PC2 scores differed significantly between *Greya*-moth communities being lower in populations with *G. politella* only (Fig. 2*F* and *SI Appendix*, Fig. S14). In addition, the cytotype × moth-community interaction was significant for PC1 scores (*SI Appendix*, Fig. S14). Cytotype explained more than three times as much of the total variation in multivariate floral morphology than either *Greya* moth community or the cytotype × moth-community interaction (PERMANOVA; Fig. 2*F*). In addition, latitude explained a significant amount of the total variation of multivariate floral morphology (PERMANOVA; Fig. 2*F*). However, the covariate latitude not only accounts for spatial autocorrelation but is also confounded with the geographical distribution of the presence/absence of the two *Greya* species (*SI Appendix*, Table S1).

Finally, we explored the direct contribution of polyploidization to variation in floral morphology by synthetically generating neotetraploids by treating diploid *L. bolanderi* seedlings from the diploid-tetraploid mixed population KAW with colchicine. Plants from this site showed the most pronounced differentiation between cytotypes among the mixed-ploidy sites in our common garden (*SI Appendix*, Figs. S10–S12). Plants in which polyploidization was induced were cross-pollinated, resulting in three F1 groups of the colchicine treatment: neotetraploids, neotriploids, and colchicine-treated plants that remained diploid. These three treatments were compared to two F1 control groups: untreated diploids (i.e., control diploids) and untreated tetraploids (i.e., control tetraploids). The results show that all 15 traits were larger in polyploids with 11 traits being significantly larger in neotetraploids than in untreated diploids and in colchicine-treated plants that remained diploid. Only three traits differed significantly between the colchicine and the control treatments (*SI Appendix*, Fig. S15). The differences among cytotypes of the colchicine treatment were significantly more pronounced than differences among cytotypes of the control treatment for four of the 15 traits (*SI Appendix*, Fig. S15).

**Fig. 3.**
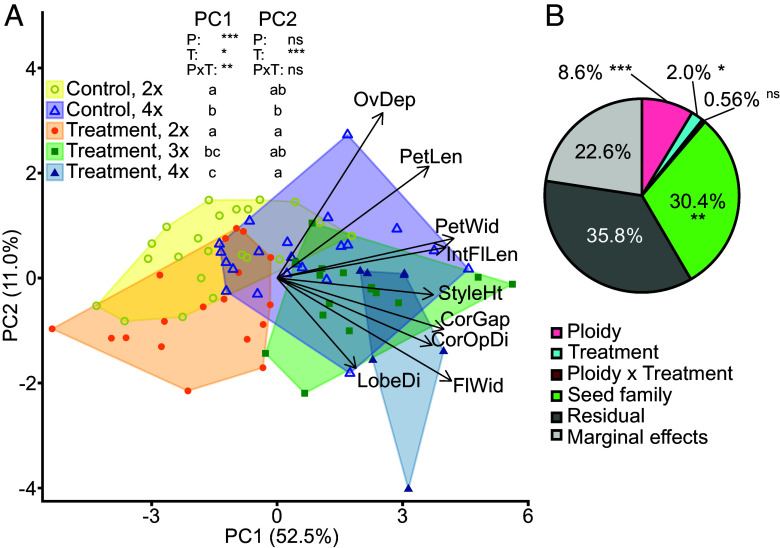
Differentiation in floral morphology of *L. bolanderi* among cytotypes in neopolyploids generated through colchicine treatment of seedlings and in control diploids and control tetraploids. (*A*) Differences among the five groups (“Control, 2×”: control diploids, “Control, 4×”: control tetraploids, “Treatment, 2×”: colchicine-treated plants that remained diploid, “Treatment, 3×”: neotriploids, and “Treatment, 4×”: neotetraploids) according to principal component 1 (PC1) and PC2. Symbols represent individuals. Symbols and areas are color-coded according to group, and correlations of the original floral traits with PCs are indicated (see also *SI Appendix*, Table S9; for full trait names, see Fig. 2). The variance explained by the PCs is given in brackets. Significance levels of the LMMs are indicated: “ns” *P* > 0.5, *P < 0.05, **P < 0.01, ***P < 0.001 (P: cytotype, T: treatment, PxT the cytotype × treatment interaction). Different lowercase letters next to the names of the groups indicate significant (*P* < 0.05) differences in pairwise post hoc comparisons among the five groups based on LMMs. (*B*) The percentage of variance in floral morphology explained by the factors cytotype (Ploidy), treatment, cytotype × treatment interaction (Ploidy × Treatment), donor-receiver seed family combination (Seed family) estimated from permutational analyses of variance are given and significance levels for these factors are indicated: “ns” *P* > 0.5, *P < 0.05, **P < 0.01, ***P < 0.001.

Colchicine-induced polyploidization had multivariate effects on floral morphology, similar to those observed in naturally occurring polyploids (Fig. 3). Multivariate floral morphology differed among the five groups both when analyzed in a PCA (Fig. 3*A* and *SI Appendix*, Table S12) and with a PERMANOVA (Fig. 3*B*). Neotetraploids and neotriploids clustered together with natural tetraploids and had higher PC1 scores, indicating larger flowers, than both natural diploids and colchicine-treated plants that remained diploid (Fig. 3*A*). PC1 scores also differed between the colchicine treatment and the control treatment, but significantly so only for neotetraploids compared to control tetraploids (Fig. 3*A*). PC2 scores did not differ among cytotypes but were lower in the colchicine treatment than in the control treatment, although significantly so only for neotetraploids and colchicine-treated plants that remained diploid compared to control tetraploids (Fig. 3*A*). Neither polyploidization nor the colchicine treatment affected the multivariate variance in floral morphology (PERMDISP: *F*_4,80_ = 0.66, *P* = 0.68; all pairwise tests: *P* > 0.05). Overall, cytotype explained 8.6% of the total variation in multivariate floral morphology, but also the colchicine treatment (2.0%) and the identity of the parental seed families (30.4%) had significant effects on multivariate floral morphology (PERMANOVA; Fig. 3*B*). However, when we performed cross-specific analyses to control for founder effects, we found similar patterns than for the overall results (*SI Appendix*, Figs. S16 and S17). Together, these results indicate that polyploidization had direct effects mainly on floral size and that the colchicine treatment had only minor effects on other aspects of floral morphology.

## Discussion

This study documents strong effects of plant ploidy level on floral morphology through comprehensive analyses of among- and within-population variation in cytotypes and floral morphology across the range of *L. bolanderi*. We unravel a complex history of repeated polyploidization that has shaped floral morphology. Plant ploidy level explained more than 15% of the total variation in multivariate floral morphology across the species range, and trait diversification from diploids was consistent across natural polyploids and experimental neopolyploids, together indicating that there are general, concerted effects of polyploidization on floral morphology. These effects primarily regard floral size, whereas the effects on floral shape are more population-specific, potentially as a result of pollinator-mediated selection.

Knowledge of the number of cytotypes present in a species, their geographical distribution, and their evolutionary history through auto- or allopolyploidization, represents a basis for understanding the evolutionary and ecological dynamics of polyploids (61). Our species-wide screening of *L. bolanderi* identified three dominant (diploid, tetraploid, hexaploid) and three rare (triploid, pentaploid, octoploid) cytotypes and a clear geographic structure in *L. bolanderi* cytotypes with multiple contact zones and a mosaic of mixed-ploidy and monomorphic populations. Our whole-genome resequencing generally supported autopolyploid origins of polyploid *L. bolanderi* plants (*SI Appendix*, Table S4), but we also found several lines of evidence consistent with low levels of introgression from *L. glabrum*. First, our analyses of the ITS region are consistent with the findings of *L. bolanderi* and *L. glabrum* sequence copy types by Kuzoff (47) in at least some *L. bolanderi* tetraploids, suggesting introgression from *L. glabrum*. This discrepancy to the nuclear whole-genome data could potentially arise from ITS being present in many paralogous copies, which can lead to confounding results when used for phylogenetic inference (62, 63). To fully resolve the ITS sharing in the species complex, a more contiguous genome assembly would be required. Further, one of the three strongest supported trees in the GRAMPA analyses was also consistent with some degree of *L. glabrum* introgression into *L. bolanderi*, but into both diploid and polyploid populations. Jointly, our analyses support a complex history of introgression rather than an allopolyploid origin of *L. bolanderi* polyploids (*SI Appendix*, Table S4) highlighting the use of whole-genome data for understanding the history of polyploidization (64, 65). Moreover, our genomic analyses suggest several independent origins of *L. bolanderi* polyploids. Clustering analysis of nuclear data suggests that the southern diploids and tetraploids form one group and the northern diploids and tetraploids another. Chloroplast network uncovers that all polyploid individuals have unique and diverged chloroplasts and form three major polyploid clusters, from which two include diploid *L. bolanderi* populations. Across analyses, the tetraploid and hexaploid BAT individuals cluster tightly, suggesting that the hexaploid has probably arisen from the union of reduced and unreduced gametes from the tetraploid (66). In contrast, three northern hexaploids cluster outside both the northern and southern clades in one of the three best supported GRAMPA trees, but the HOP chloroplast sequence clusters with the chloroplasts of both diploid and tetraploid populations. Thus, we cannot distinguish among alternative routes by which they have formed, e.g. through additional hybridization events involving autotetraploid and diploid plants, as suggested in *Campanula rotundifolia* (Campanulaceae) (66), or through union of reduced and unreduced gametes (66). Additional sequencing, producing long read and population data for polyploids to enable allele frequency spectrum based simulations (*cf.* 67), haplotype phasing, and formal introgression analyses, is paramount for testing the hypothesis of introgression between *L. glabrum* and *L. bolanderi*. In general, our findings on the genomic underpinnings of polyploids are crucial for interpreting how genomic architecture interacts with ecological selection and shape the floral phenotype of *L. bolanderi*.

Despite the complex history of repeated polyploidization and potential *L. glabrum* introgression, our comprehensive morphological analyses reveal consistent effects of plant ploidy level on floral traits across the *L. bolanderi* range. Several previous studies have found differences among cytotypes in floral size (e.g., 10, 14, 15, 21, 28–31) and in combinations of floral traits (i.e., floral shape) (e.g., 15, 32), although studies across a species range are rare (but see 15, 40, 41). By including more than 1,400 individuals from 27 populations covering the entire range of *L. bolanderi*, we show that polyploids had larger flowers and a different floral shape than diploids. The increased floral size may be a direct result of polyploidization, if the increased DNA amount results in an increased cell size (e.g., 10, 11). However, in *L. bolanderi*, hexaploids had a similar floral size and shape as tetraploids, regardless of the higher DNA amount. This is consistent with some other systems (29) and indicates that the floral size variation is not directly linked to the increased DNA amount in higher ploidy levels. Differences between diploids and tetraploids varied across mixed-ploidy populations. In combination with the complex history of polyploidization in *L. bolanderi* revealed by our genomic analyses, these results add to the evidence that independent, recurrent formation of polyploid lineages can have different effects on floral traits in different polyploid lineages (15). Such origin-specific effects alone and/or in combination with divergent selection could contribute to an increased floral trait variation in polyploids compared to diploids.

In addition to a distinct geographical distribution of the cytotypes, limited gene flow and genetic drift can cause floral trait differences to increase with increasing geographical distance (e.g., 46, 68, 69). Yet, in *L. bolanderi*, populations of the same cytotype had a more similar floral morphology than populations of different cytotype also after correcting for geographic distance, and floral morphology differed between cytotypes within mixed-ploidy populations where geography is naturally held constant. However, also after correcting for ploidy level, populations growing in closer proximity were more similar, indicating convergent local selection on floral morphology and/or local gene transfer through backcrossing. This result indicates that several other nonmutually exclusive evolutionary processes may reinforce or mitigate polyploidization-induced differences.

In *L. bolanderi*, the pollinating seed predator *G. politella* and its closely related pollinating herbivore *G. obscura* constitute major selective agents on floral traits (e.g., 45, 50, 53). Our study confirmed significant effects of the local presence/absence of the two *Greya* pollinators on variation in floral morphology, but plant ploidy level explained more than three times as much of the local floral variation than *Greya* moths. The significant, but relatively modest, interaction effect between plant ploidy and moth community may indicate that some of the response to moth pollination may be cytotype-specific or that the two moth species may differ in their use of plants of different ploidies, as was found in previous work on *H. grossulariifolia* (15, 22, 26). In this relative of *Lithophragma,* diploid and tetraploid plants differed in pollinator communities, and *G. politella* preferred to attack tetraploids, whereas another *Greya* species, *G. piperella*, attacked a higher proportion of diploids (15, 22, 26).

A recent meta-analysis has shown that macroscopic morphological traits were larger in polyploids than in diploids and did not differ between neopolyploids and established polyploids (70). Detailed studies of synthetically generated neopolyploid *H. grossulariifolia* have shown that polyploidization itself causes phenotypic divergence (12) and that this may be reinforced by phenotypic selection (18). Our experimental induction of neopolyploid *L. bolanderi* from one mixed-ploidy population also revealed that neotetraploids were larger than their diploid progenitors. However, our finding that the morphological divergence was larger between neotetraploids and diploids than between natural tetraploids and diploids indicates that natural selection may have converged, rather than diverged, floral morphology of different cytotypes at our focal site. In addition, the neotetraploids differed in size but not in shape from their diploid predecessors. In the light of our results of the relative contribution of plant ploidy and *Greya* moths to floral divergence across populations of established cytotypes, this indicates that polyploidization mainly imposes divergence in floral size, whereas *Greya* pollinators impose selection primarily on floral shape in *L. bolanderi*. Thus, overall, our results suggest that the combined effect of direct changes in floral morphology induced by polyploidization, local selection, and putative different origin of polyploids in *L. bolanderi* provide a likely explanation for the morphological differences among cytotypes and the variation in magnitude and direction of these differences across populations.

By combining our genomic and phenotypic datasets on *L. bolanderi* with experimental comparisons of established cytotypes and neopolyploids, our study casts some light on the long-standing discussion about the presence of a so called “polyploidy paradigm”(3), i.e., whether there are general and predictable genetic and phenotypic changes following polyploidization. Our results indicate that (i) the intriguing floral size and shape variation of *L. bolanderi* has been generated through repeated polyploidization events, albeit with a yet to be resolved minor introgression also from *L. glabrum*. Further, (ii) the phenotypic effects on floral morphology vary among genetically and geographically diverse populations, likely representing different polyploidization events, and (iii) the current floral phenotypes of *L. bolanderi* have been molded also by subsequent, local selection from their specialized *Greya* moth pollinators (also previously shown in 45, 50, 53) and other selective agents. More generally, our findings highlight how large-scale genomic changes can have immediate and strong effects on trait variation that may mediate species interactions and be the basis for selection to act on. Future comparative studies should focus on understanding how these genomic and ecological factors collectively influence the evolution of angiosperm diversification, and how the traits shaped directly by polyploidization may mediate ecological interactions between plants and their mutualists and antagonists, by providing novel trait variation for natural selection to act upon.

## Materials and Methods

### Study Species.

*L. bolanderi* A. Gray (Saxifragaceae) is a perennial, self-incompatible herb largely restricted to the open, grassy areas in the oak woodland of the western slopes of the Sierra Nevada in central California, up to an altitude of 2,000 m a.s.l. (44, 45) (Fig. 1*A*). In natural populations, individuals grow one to few 20 to 40 cm long floral stems, each producing on average 10 to 20 loosely separated flowers that sequentially open from the bottom of the indeterminate ear. Plants grown in the greenhouse can sometimes reach a height of 70 to 80 cm and produce more than 20 stalks and more than 100 flowers. However, flower diameter and corolla-opening diameter did not differ between greenhouse-grown plants and plants within natural populations (*SI Appendix*, Fig. S18). The white, rarely pink, fragrant flowers have five ovate to elliptic petals with edges that vary from entire to lobed (44). Both floral scent and floral morphology have been shown to be highly variable and to be important for the interaction of *L. bolanderi* with its specialized pollinators of the genus *Greya* (Prodoxidae) (45, 50, 53, 71–73). After flowering and fruiting, plants wither and regrow from root bulbils the following spring.

### Greenhouse Common-Garden.

Seeds were collected in 29 natural *L. bolanderi* populations between 2004 and 2017. Population details and sample sizes are listed in *SI Appendix*, Table S1. We grew plants in a greenhouse common-garden in two cohorts in two consecutive years. We sowed 20 seeds per seed family from 2 to 38 seed families (seeds collected from a single plant being half- and/or full-sibs) per population. When well established, up to five seedlings per seed family were transplanted to individual pots and cultivated until flowering. For exact growth conditions, see *SI Appendix*, Supporting Text S1.

### Ploidy Level Assessment.

We assessed the number, distribution, and abundance of different cytotypes based on relative ploidy-level analyses of *L. bolanderi* plants from a total of 40 populations (*SI Appendix*, Table S1) using flow cytometry. For the plants from the 29 populations grown in the common garden, we used a slightly adjusted two-step flow cytometry protocol initially described by Doležel et al. (74) (*SI Appendix*, Supporting Text S2.1). For the remaining 11 populations, we collected leaf material of 16 to 41 plants from the natural populations in spring 2019 (*SI Appendix*, Table S1) and shipped them to Plant Cytometry Service (https://www.plantcytometry.nl/) for relative ploidy-level analysis (*SI Appendix*, Supporting Text S2.2). The flow cytometry analyses conducted by Plant Cytometry Service were highly comparable with those we conducted (*SI Appendix*, Supporting Text S2.3 and Fig. S19). Discontinuities in the ratio of the relative fluorescence of the sample and the IS were used to identify relative ploidy groups, and the information on the known ploidy levels from karyological counts by Taylor (44) was used to assign these groups to absolute ploidy levels (*SI Appendix*, Supporting Text S2.1 and S2.2).

### Evolutionary Origin of Polyploids.

We explored the evolutionary origin of diploid and tetraploid *L. bolanderi*, i.e., the minimal number of polyploidization events and whether those could be attributed to auto- or allopolyploidization, in two different ways. First, we extended the analyses based on ITS region sequences of the nuclear ribosomal DNA conducted by Kuzoff et al. (47), which suggested an allopolyploid origin of polyploid *L. bolanderi* involving diploid *L. bolanderi* and *L. glabrum* as parental species, using an increased geographic sampling (*SI Appendix*, Supporting Text S3.1).

Second, we acquired a genome-wide insight into the polyploidization history of *L. bolanderi* based on whole-genome data. We sequenced a diploid *L. bolanderi* individual from the SMR population resulting in 72 times coverage of PacBio sequel data and assembled a reference genome using HGAP4 in SMRTlink version 8.0.0.79519. The resulting assembly had an N50 of 196,527 and a BUSCO score of 92.7 and was annotated using the BRAKER2 (75) pipeline resulting in a protein set with a BUSCO score of 90% (details in *SI Appendix*, Supporting Text S3.2). We then resequenced four diploid individuals from each of the mixed diploid-tetraploid populations MIN and MXN as well as from BAM, the northernmost diploid population included in the common garden, one tetraploid individual from each of six populations, and one hexaploid individual each from four populations for *L. bolanderi* and four individuals of the population POR for *L. glabrum* (*SI Appendix*, Table S1 and S13). The *L. bolanderi* individuals were genetically identical to the plants used for ploidy-level analysis and floral morphology measurements (*SI Appendix*, Supporting Text S3.3). We extracted DNA using a DNeasy^®^ Plant Mini Kit (QIAGEN^®^) (*SI Appendix*, Supporting Text S3.3). Library preparation and Illumina paired-end sequencing on NovaSeq6000 system was performed at SciLifeLab in Solna, Sweden (https://www.scilifelab.se/). We mapped reads trimmed with fastp (76) of both species to the reference genome of *L. bolanderi* with bwa version 0.7.18 (77), identified and filtered duplicated reads, and called SNPs with GATK version 4.1.4.1 by using Haplotypecaller to call variants per individual, for their specific ploidy levels, and then aggregate variants using GenotypeGVCFs (78, 79). Details on filtering are presented in *SI Appendix*, Supporting Text S3.4. Mean coverage of individuals after filtering is reported in *SI Appendix*, Table S13.

We applied nine complementary analyses to uncover the evolutionary origin of polyploid *L. bolanderi* (*SI Appendix*, Table S4). First, we compared mapping rates between *L. glabrum* diploid and polyploid *L. bolanderi* samples. Second, we displayed genetic similarity among ploidy levels and populations by PCA run in the *adegenet* R package (80) based on 21,667SNPs filtered for linkage disequilibrium and thinned to include only every 15th SNP (*SI Appendix*, Supporting Text S3.5). Third, we used the same SNP set in a clustering analyses in STRUCTURE (57), shown to be robust in analysis of mixed-ploidy populations (81), identifying 4 clusters as the best fit (*SI Appendix*, Supporting Text S3.5 and Fig. S20). Fourth, we inferred phylogenetic relationships between populations and ploidy levels using allele frequency covariance graphs implemented in TreeMix (58, 59). TreeMix was run for 30 iterations for up to 10 migration edges (M), with 5 identified as the best fit by OptM (82) (*SI Appendix*, Supporting Text S3.6 and Fig. S21). The phylogeny remained the same for all values of M. Fifth, we estimated parsimony scores for all possible MUL trees using GRAMPA version 1.4 (60) based on 46 gene trees phased with WhatsHap (83), to examine whether trees with multimapping of polyploids to both *L. bolanderi* and *L. glabrum*, as expected under allopolyploidy, was the most parsimonious explanation. Sixth, we called variants for cpDNA using GATK HaplotypeCaller with ploidy of 1 and produced individual consensus fasta files using VCFtools (84) (*SI Appendix*, Supporting Text S3.8 and Fig. S22. Based on these, a cpDNA tree was built using IQ-TREE 2 (85) with GTR+G model and 1,000 bootstraps, and a median joining haplotype network was built using POPART (86) (*SI Appendix*, Supporting Text S3.8). Seventh, we estimated the site frequency spectra separately for tetraploid and hexaploid *L. bolanderi* individuals to examine whether we found peaks at intermediate frequencies, as expected in allopolyploids (56) (*SI Appendix*, Supporting Text S3.8). Eighth, we assessed allele pairing behavior and verification of ploidy inferences by conducting k-mer analysis with FastK (v1.1) and GenomeScope2 (v2.0.1) (*SI Appendix*, Supporting Text S3.10). Finally, we assess whether tetraploid (4×) and hexaploid (6×) individuals exhibit patterns consistent with auto- or allopolyploidy by calculating the proportion of *L. bolanderi*-type alleles for alleles with fixed differences between diploid *L. bolanderi* and *L. glabrum* (*SI Appendix*, Supporting Text S3.11).

### Floral Morphology Measurements.

For each flowering plant in the common garden, we selected one flower—the second fully open flower from the top of an inflorescence—or, in rare cases, when this flower started withering or was malformed, the first or third flower. We measured the flower diameter and the corolla-opening diameter using a digital caliper (Fig. 2*A*) and then stored the flower in 70% EtOH. Flowers were dissected and photographed, and, based on these photos, 13 additional floral morphology traits were quantified using the image processing program ImageJ (https://imagej.net/Fiji) (Fig. 2*A*) (for details, see *SI Appendix*, Supporting Text S4). In addition, we categorized the shape of the petal edge as either whole (0) or lobed (1). As the measurements of the flowers were apportioned among three persons, we took measures to guarantee repeatability (*SI Appendix*, Supporting Text S4).

### Synthetic Polyploidization Experiment.

Neopolyploids were generated by synthetically inducing polyploidization using colchicine in diploid *L. bolanderi* seedlings grown from seeds collected from the natural, mixed-ploidy population KAW, the population with the most pronounced floral morphology differentiation between established diploids and tetraploids (*Results*). As the seeds were of the same KAW seed families as those included in the common garden, their ploidy level was known. For details on plant growing, see *SI Appendix*, Supporting Text S5.1. Approximately 4.5 to 7 wk after sowing, we transferred the seedling into Petri dishes, incubated them in a 0.2% colchicine solution over night for approximately 16 h at room temperature in a darkened fume hood, rinsed them in DWH_2_O, and planted them back into soil (*SI Appendix*, Supporting Text S5.2). Diploid and tetraploid seedlings treated with DWH_2_O instead of the colchicine solution were used as control plants.

Colchicine is the most widely used chemical to synthetically induce polyploidization (39, 87). A colchicine treatment usually not only results in neotetraploids but also in plants that remain diploid and/or in diploid-tetraploid chimeric plants (12, 87). Thus, the ploidy level of each plant was assessed using flow cytometry (*SI Appendix*, Supporting Text S5.3). We conducted hand-pollination crossings among different colchicine-treated plants, only using plants with at least some tetraploid nuclei, and within the diploid and tetraploid control plants (*SI Appendix*, Supporting Text S5.4) to minimize effects of colchicine other than inducing polyploidization (11, 12). The seeds resulting from these crossings were sown to grow F1 plants (*SI Appendix*, Supporting Text S5.1). Ploidy level of all plants of the colchicine-treated group and of a subset of the plants of the diploid and the tetraploid control groups was verified using flow cytometry (*SI Appendix*, Supporting Text S5.3). When plants were fully flowering, we selected one flower per plant for floral morphology measurements (see above and *SI Appendix*, Supporting Text S5.5). For sample sizes and identities of plants available for measurements, see *SI Appendix*, Table S1 and Fig. S23.

### Statistical Analyses.

All statistical analyses were conducted in the statistical software program R version 4.0.3 (88) unless stated otherwise. For all analyses, we only included the three dominant cytotypes, i.e., diploids, tetraploids, and hexaploids (*Results*), and individuals when there were individuals from at least four seed families per cytotype and population (*SI Appendix*, Table S1).

At the univariate level, we assessed how individual floral morphology traits differed among cytotypes by running a linear mixed-effect model (LMM) for each of the 15 numeric traits and a generalized linear mixed-effect model (GLMM) for the categorical trait petal-edge-shape with the functions *lmer* and *glmer*, respectively, in the R package *lme4* (89) (for model details, see *SI Appendix*, Table S14). In these and subsequent similar models, post hoc tests for pairwise comparisons were conducted using the function *glht* in the R package *multcomp* (90) with Tukey contrasts and holm adjustment, analysis of deviance tables were generated using the function *Anova* in the R package *car* (91) to extract p-values, and adjusted means and 95% CLs were estimated using the *effects* R package (91).

For the multivariate analyses, we only included continuous traits with pairwise correlations <0.7 (*SI Appendix*, Fig. S9). First, we computed a principal component analysis (PCA) on traits scaled to unite variance, using the function *PCA* in the R package *FactoMineR* version 2.3 (92) (*SI Appendix*, Table S8). We then compared the principal component (PC) scores among cytotypes using LMMs (*SI Appendix*, Table S14).

Next, we compared mean multivariate floral morphology among cytotypes using a permutational multivariate ANOVA (PERMANOVA) in PRIMER 6.1.15 (93) with PERMANOVA+ for PRIMER 1.0.5 (94) (*SI Appendix*, Table S15). These PERMANOVAs were also used to estimate the relative contribution of cytotype to the total variation in floral morphology compared to the contribution of population, seed family, and year when the plants were grown based on the sum of squares. In addition, we tested whether the multivariate variation in floral morphology differed among cytotypes by running permutational analyses of multivariate dispersion (PERMDISP) in PRIMER 6.1.15 (93) with PERMANOVA+ for PRIMER 1.0.5 (94) (*SI Appendix*, Table S15).

We tested the relationship among floral morphology, cytotype, and geography by running partial Mantel tests using the function *mantel* in the R package *ecodist* (95) (*SI Appendix*, Table S13). This analysis was conducted at the population level using population means for the floral morphological traits estimated from LMMs (*SI Appendix*, Table S17).

The presence of mixed-ploidy populations (Fig. 1*A*) allowed us to assess whether and how floral morphology differed among cytotypes independent of geography. We used the subset of the numeric floral trait values and PC scores for the three diploid-tetraploid mixed populations KAW, MIN, and MXN and the tetraploid-hexaploid mixed population BAT to assess differences in univariate and multivariate floral morphology among cytotypes within and among mixed-ploidy populations using LMMs, PERMANOVAs, and PERMDISPs (*SI Appendix*, Table S18).

We assessed the relative contribution of plant ploidy and the *Greya* moth community to the geographic divergence in floral morphology using linear models (LMs) for the univariate traits and the PC scores for PC1 and PC2 and a PERMANOVA and a PERMDISP for multivariate floral morphology (*SI Appendix*, Table S19). As the information on the *Greya* moth community (*SI Appendix*, Table S1) was only available at the population level, all analyses were conducted using population means of the 15 continuously varying floral traits and the PC scores estimated from LMMs (*SI Appendix*, Table S17).

We tested for direct effects of polyploidization on floral morphology, that is, whether neopolyploids differed from diploids and established tetraploids, using LMMs for the univariate traits and a PCA with LMMs on the PC scores, PERMANOVAs, and PERMDISPs for multivariate floral morphology (*SI Appendix*, Table S20). In addition, we used the subset of the data for a donor-receiver seed family lineage that comprised three control diploids, three neotriploids, and three neotetraploids, and for a donor-receiver seed family lineage that comprised two diploid control plants and two colchicine-treated plants that remained diploid to assess differences among cytotypes and between treatments independent of seed family effects using PERMANOVAs and PERMDISPs (*SI Appendix*, Table S20).

## Supplementary Material

Appendix 01 (PDF)

## Data Availability

(1) morphological and flow cytometry data (2) whole genome data (3) Sanger sequencing of the ITS region data have been deposited in (1) Dryad (2) NCBI Sequence Read Archives (SRA) (3) GenBank [(1) https://doi.org/10.5061/dryad.k0p2ngfm3 (96) (2) BioProject ID PRJNA1113201 (97) (3) PP828641-PP828659 (98)]. Previously published data were used for this work [Internal transcribed spacer (ITS) sequences from Kuzoff et al. 1999 avaiable on GenBank with the accession numbers AF158916.1 to AF158962.1.].
