# Supplementary material for: Repeated polyploidization shapes divergence in floral morphology in Lithophragma bolanderi (Saxifragaceae)
Source: Proc Natl Acad Sci U S A. 2025 Aug 13;122(33):e2505119122. doi: 10.1073/pnas.2505119122 (PMC12377753; doi:10.1073/pnas.2505119122)
Supplement: Supplementary file 1 — Appendix 01 (PDF) [file pnas.2505119122.sapp.pdf]

PNAS

Supporting Information for

Repeated polyploidization shapes divergence in floral morphology in *Lithophragma bolanderi* (Saxifragaceae)

Karin Gross, Homa Papoli Yazdi, Elisabeth Schlager, Jodie Lilley, Andrés Romero-Bravo, Anna Runemark, John N Thompson, and Magne Friberg

Corresponding author: Karin Gross
Email: karin.gross@plus.ac.at

This PDF file includes:

Supporting Text S1 to S5
Figures S1 to S23
Tables S1 to S20
SI References

Supporting Text

S1 Growth conditions of the plants of the common garden

Seeds were collected in 29 natural *Lithophragma bolanderi* populations between 2004 and 2017. For each individual, the seeds (when possible, pooled from multiple capsules) were stored in separate glassine bags. The seeds collected from a single individual represent a seed family and are, thus, half and/or full-sibs, because *L. bolanderi* is self-incompatible (1). Plants were grown in two cohorts. The seeds for the first cohort were sowed in September/October 2016 and the seeds for the second cohort in August/September 2017.

We sowed 20 seeds per seed family into 9×9×7-cm plastic pots filled with S-jord (Hasselfords, Granngården, Sweden) potting soil. Freshly sowed pots were kept in a growth chamber (BioChambers SPC-56, BioChambers Incorporated, Winnipeg, Manitoba, Canada) at the Department of Plant Ecology and Evolution at Uppsala University, Sweden, for germination under the following conditions: 14 h light/10 h dark, 400 µmol e light intensity during the light phase, 15°C during the light phase and 10°C during the dark phase, 60% humidity, and 65% fan speed. Pots were covered with transparent plastic foil to avoid drying out and watered approximately twice a week with deionized water. The position of the pots was regularly shuffled to equalize growing conditions. After approximately seven weeks, or when seedlings were well established, we transplanted, when available, up to five seedlings per seed family into pots with one seedling per pot.

The individualized plants were kept in a growth room at a 14 h light/10 h dark rhythm, with 150 $\mu Em^{-2}s^{-1}$ light intensity during light phase, and at 15°C during the light phase and 10°C during the dark phase. We watered the plants once to twice a week with deionized water and added liquid fertilizer (10 ml/10 l H_2O) to the water. The position of the pots was regularly shuffled to equalize growing conditions. After another approximately seven weeks or when plants were well established, we moved them to a greenhouse (14 h light/10 h dark rhythm with natural and artificial light during light phase, approximately 18°C during the light phase and approximately 16°C during the dark phase). Plants were automatically watered with nutrient enriched water (1.5 electrical conductivity; SW Bouyant Rika T 3-1-5) every 72 h. Plants were kept in the greenhouse for the entire flowering season, which lasted approximately four to five months. When the aboveground part of a plant withered, it was cut, and the soil and root bulbils in the pots were air-dried for at least two weeks. We then carefully removed the dry soil from the bulbils and stored the bulbils in small paper bags at a dry place at room temperature. In this way, the bulbils can be stored up to several years and genetically identical individuals can be grown when (a piece of) the bulbil is planted into soil and watered.

S2 Ploidy-level assessment

S2.1 Plants from the common garden. When plants were fully established, we collected 1-2 cm^2 fresh *L. bolanderi* leaf material into a 2-ml Eppendorf tube containing a metal bead (ø 5 mm) and added approximately 0.5-1 cm^2 fresh leaf material of *Brassica napus* (Brassicaceae) as internal standard (IS). The *B. napus* plants were grown together with the *L. bolanderi* plants. To each tube, we added 1 ml ice cold Baranyi's solution (2, 3). Leaves were crushed using the bead beating method (4, 5) by shaking the samples 1-2 times for 30 s at 25 HZ using a TissueLyser (Quiagen Retsch MM300). We filtered this solution through a 30-µm filter (CellTrics®, Sysmex Partec GmbH, Görlitz, Germany) into a 1.5-ml Eppendorf tube. The filtrate was centrifuged at 380 g (≙ 2000 RPM) at room temperature for 5 min using a Heraeus Biofuge Pico Microcentrifuge (rotor ø 17 cm). After carefully removing the supernatant, we added 40 µl ice cold Baranyi's solution and resuspended the nuclei by pipetting up and down several times. Samples were then stored at 4 °C until analysis.

For flow cytometry analysis, we resuspended the nuclei by tapping or vortexing the Eppendorf tube. We transferred 30 µl of the solution into a flow cytometer tube, added 160 µl Otto II solution containing 4 $\mu l\ ml^{-1}$ DAPI (4',6-diamidino-2-phenylindole) as fluorochrome (3), and incubated the mixture for approximately 30 s while slightly shaking. The mixture was then diluted with approximately 300 µl PBS (phosphate-buffered saline). Samples were run on a BD LSRFortessa™ flow cytometer at BIOVIS (Biolgical Visualisation) at Uppsala University, Sweden (https://biovis.uu.se/). Samples were run at a rate of 10,000-20,000 events per s (whenever possible) until a total count of 10,000 particles was reached or all sample solution was used up. We first analyzed one plant per seed family. In populations with only one cytotype, we inferred that the non-measured plants had the same cytotype as the measured plants, whereas in populations with more than one cytotype, we analyzed all available individuals, because, when sired by different fathers, individuals of the same seed family could have different ploidy levels.

In each flow cytometric histogram (for some representative examples, see SI Appendix, Fig. S1C), we integrated the *L. bolanderi* peak and the IS peak and calculated the sample:IS ratio as the

median of the *L. bolanderi* peak divided by the median of the IS peak. The mean ± SD number of nuclei counted was 5038 ± 2443 for the *L. bolanderi* peak and 1947 ± 1364 for the IS peak, and the mean ± SD coefficient of variation was 4.01 ± 1.12 for the *L. bolanderi* peak and 3.23 ± 0.97 for the IS peak. We looked for discontinuities in the sample:IS ratios. This resulted in five separated sample:IS ratio groups (SI Appendix, Fig. S1A).

There was considerable variation in the sample:IS ratios also within groups and among populations. Thus, we primarily focused on the samples within a population to be able to clearly assign samples to a group. We calculated the mean ± one standard deviation (SD) of the sample:IS ratios within a group (SI Appendix, Fig. S1A). This showed that, on average, the intervals between consecutive groups were quite evenly distributed and that, for example, the sample:IS ratio group with the third lowest values had values roughly twice as high as the group with the lowest and that the group with the highest values had values roughly three times as high as the group with the lowest values. Together, this indicates that the five sample:IS ratio groups reflected five different relative ploidy levels. We then used the information in the paper by Taylor (6) on the known ploidy levels from karyological counts to assign our relative ploidy levels to absolute ploidy levels. More specifically, we assigned our relative ploidy level with the lowest sample:IS ratio values to the lowest ploidy level found by Taylor (6), i.e. to the absolute ploidy level diploid (2n = 14). Three of the diploid samples Taylor (6) found were from the center of the distribution range of *L. bolanderi* and, thus, are from the vicinity of where we found most of the samples assigned to our relative ploidy level with the lowest sample:IS ratio values. In addition, when comparing the relative abundance of ploidy levels, diploid samples had the highest relative abundance in the samples included in the paper by Taylor (6) and also in our samples, the samples assigned to the relative ploidy level with the lowest sample:IS ratio values had the highest relative abundance. Correspondingly, we assigned our relative ploidy level with the third lowest sample:IS ratio values to the absolute ploidy level tetraploid (2n = 28 in Taylor (6)), our relative ploidy level with the second highest values to the absolute ploidy level pentaploid (2n = 35 in Taylor (6)), and our relative ploidy level with the highest values to the absolute ploidy level hexaploid (2n = 42 in Taylor (6)). One of the tetraploid samples from Taylor (6) was from the south of the distribution range of *L. bolanderi* and, thus, close to where we found a lot of samples of our relative ploidy level with the third lowest sample:IS ratio values. Similarly, two of the hexaploid samples from Taylor (6) were from the north of the distribution range of *L. bolanderi* and, thus, close to where we found most of our samples of the relative ploidy level with the highest sample:IS ratio values. Our relative ploidy level with the second lowest sample:IS ratio values had no equivalent in the samples in Taylor (6) and, thus was a newly detected ploidy level. As this relative ploidy level had sample:IS ratio values between diploids and tetraploids, we assigned it to the absolute ploidy level triploid. Individuals assigned to different ploidy levels were referred to as cytotypes. The relatively high variation in sample:IS ratios also within ploidy levels and among populations in *L. bolanderi* is an interesting finding. If this variation in sample:IS ratios within groups reflects variation in DNA content, this could indicate, for example, the presence of aneuploids or accessory chromosomes. Indeed, Taylor (6) reported that in *Lithophragma*, some individuals contain satellite or accessory chromosomes. An alternative, non-mutually exclusive explanation for the relatively high variation in sample:IS ratios within groups is that there might be backcrosses of the intermediate cytotypes (triploids, pentaploids) with the dominant cytotypes (diploids, tetraploids, hexaploids). Some of the samples we assigned to one of the intermediate ploidy levels might actually be such backcrosses into one of the dominant cytotypes. Because of this and as there were relatively few individuals with an intermediate ploidy level, we focused in all subsequent analyses on the dominant cytotypes diploids, tetraploids, and hexaploids.

S2.2 Field-collected leaf material. We collected approximately 2-4 cm^2 leaf material of well-established *L. bolanderi* plants in natural populations that were not included in the common garden. The leaf material of each plant was wrapped in moist paper towel and enclosed in a separate zip-lock bag to keep it fresh. We then shipped these samples to the Dutch company Plant Cytometry Service (https://www.plantcytometry.nl/) for relative ploidy-level analysis. There, *Allium schoenoprasum* (Amaryllidaceae) was used as IS and Plant Cytometry Service sent us the sample:IS ratio. We used the same procedure as we used for the samples from the common garden to identify sample:IS ratio groups (see SI Appendix, Supporting Text S2.1). In the field-collected leaf material samples, we found, however, a sixth sample:IS ratio group comprising only one sample (SI Appendix, Fig. S1B). This relative ploidy level had the highest sample:IS ratio values, but had, as the triploid ploidy level, no equivalent in the samples in Taylor (6) and, thus was a newly detected ploidy level. As this relative ploidy had sample:IS ratio levels four times as high as the relative ploidy level with the lowest sample:IS ratio values (diploid), it was assigned to the absolute ploidy level octoploid.

S2.3 Comparability of the two flow-cytometry protocols. One major difference between the two flow-cytometry protocols was that we used *B. napus* as IS, whereas Plant Cytometry Service used *A. schoenoprasum*. Thus, we grew some *L. bolanderi* plants, from which we knew their cytotype based on our flow-cytometry analyses to assess the comparability of the two flow-cytometry protocols. In particular, we grew six diploids (three from MIN, three from SMR), four tetraploids (one from MBL, three from MIN), and three hexaploids from JMR (for details on the populations, see Fig. 1A and SI Appendix, Table S1) from seeds in September 2018 in the greenhouse facility at Lund University, Sweden. These seeds derived from crosses between individuals of the same cytotype and from the same population but from different seed families of plants grown from root bulbils of the greenhouse common-garden plants (see SI Appendix, Supporting Text S1). The sowing and growing procedure was the same as for the greenhouse common-garden plants (see main text and SI Appendix, Supporting Text S1) except for the following differences. Pots with freshly sowed seeds were kept in a small greenhouse room at a 14 h light/10 h dark rhythm with a 165 µmol m^{-2} s^{-1} light intensity during the light phase and at approximately 15°C. After transplanting, the plants grown from seeds were kept in another, larger greenhouse at a 14 h light/10 h dark rhythm with natural and artificial light during light phase and at a temperature averaging around 20°C. Alongside these *L. bolanderi* plants, we grew some *B. napus* plants. When plants had fully developed fresh leaves, we collected leaf material of the 13 *L. bolanderi* individuals and some leaves of *B. napus* and shipped them to Plant Cytometry Service. There, the relative ploidy level of each *L. bolanderi* sample was once analyzed with *B. napus* as IS and a second time with *A. schoenoprasum* as IS.

We assessed the comparability of the flow-cytometry protocol by Plant Cytometry Service with the one we used in two ways. First, we compared the sample:IS ratios measured by Plant Cytometry Service using *B. napus* as IS with the mean sample:IS ration of the two parental plants we quantified using *B. napus* as IS to assess the comparability of the ploidy levels quantified by the two different flow cytometers and/or persons analyzing the samples. For one parental plant, the flow cytometry analysis was unfortunately not possible. We, thus, calculated the mean sample:IS ratio of the other individuals of that plant's seed family for which we could measure the sample:IS ratio. The sample:IS ratios we measured and those measured by Plant Cytometry Service were highly correlated (SI Appendix, Fig. S19A). Second, we compared the sample:IS ratios with *B. napus* as IS with the sample:IS ratio with *A. schoenoprasum* as IS, both measured by Plant Cytometry Service to assess the comparability of the ploidy levels quantified through the two different internal standards. The estimates of relative ploidy level were highly correlated between samples analyzed with *A. schoenoprasum* as IS and those analyzed with *B. napus* as IS (SI Appendix, Fig. S19B).

S3 Evolutionary origin of polyploids

S3.1 Sequencing of the internal transcribed spacer (ITS) region of the nuclear ribosomal DNA. *Lithophragma bolanderi* plants from 27 populations represented by one to two individuals per cytotype from different seed families (SI Appendix, Table S1) were grown from root bulbils of the greenhouse common-garden plants (see SI Appendix, Supporting Text S1). Thus, these plants were genetically identical to the plants used for ploidy-level analysis and floral morphology measurements. In addition, we planted root bulbils of the nine other *Lithophragma* species *L. affine*, *L. campanulatum*, *L. cymbalaria*, *L. glabrum*, *L. heterophyllum*, *L. maximum*, *L. parviflorum*, *L. tenellum*, and *L. trifoliatum*. However, most of the bulbils did not sprout and DNA could only be extracted from one individual each of *L. cymbalaria* and *L. heterophyllum* (SI Appendix, Table S1). A 0.25-0.50 cm^3 sized (piece of) root bulbil was placed into a plastic pot filled with potting soil and slightly covered with soil. The growing procedure was the same as for the greenhouse common-garden plants (see SI Appendix, Supporting Text S1) except for the following difference. Pots with freshly planted root bulbils were kept in a small greenhouse room at a 14 h light/10 h dark rhythm with a 165 µmol m^{-2} s^{-1} light intensity during the light phase and at approximately 15°C.

When plants were well established and leaves were fully developed but still fresh, plants were moved into a cold room (temperature: 4°C; 24 h dark) for 24-72 h to maximize DNA extraction yields (7). Two to three young, fresh leaves (approximately 4 cm^2 per leaf) per plant were collected into a tea filter bag (Cilia® Tea Filter M, Mellita) and were dehydrated for approximately 24 h in a plastic box filled with silica gel.

From the dehydrated leaf material, we extracted DNA with a DNeasy® Plant Mini Kit (QIAGEN®), following the standard protocol provided by the company with minor adjustments. We incubated the sample with RNase for 15 min instead of for 10 min. Prior to the PCR, the concentration (mean ± 1 standard deviation: 58.14 ± 30.63 ng/µl) and quality (mean A260/280 ratio ± 1 standard deviation: 1.82 ± 0.05) of the DNA extractions were assessed using a NanoDrop 8000. We selected primers and used the PCR protocol as described in Kuzoff et al. (8) with minor modifications. For the

entire ITS region, the PCR primers were N-nc18s10 (forward primer: 5'-AGGAGAAGTCGTAACAA) anchored in 18S rDNA and C26A (reverse primer: 5'-GTTTCTTTTCCACCGCT) anchored in 26S rDNA. For a separate analysis for the ITS2 subregion the PCR primers were ITS3 (forward primer: 5'-CGATCGATGAAGAACGTAGC) anchored in 5.8S and ITS4 (reverse primer: 5'-TCCTCCGCTTATTGATATGC) anchored in 26S. The PCR reaction mixes contained 1 µl of 10X buffer, 0.6 µl of 25 mM $MgCl_2$, 0.8 µl of 1.25 mM dNTPs, 0.5 µl of 10 µM N-nc18s10 primer, 0.5 µl of 10 µM C26A primer, 0.08 µl of AmpliTaq DNA polymerase, 1.5 µl DNA extract (concentrations ranging from 15.3 to 179.9 ng/µl), and 5.52 µl of Milli-Q deionized water. The PCR reactions were performed using a Bioer LifePro Thermal Cycler (TC-96/G/H(b)A) and the following cycle: heating at 94°C for 2 min; 35 cycles of 94°C for 1 min, 50°C for 1 min, and 72°C for 1.5 min; a terminal extension at 72°C for 5 min; and an indefinite hold at 4°C. The results of the PCR reaction were checked by loading 2µl of the PCR product on a 2% agarose gel stained with GelRed for electrophoresis and UV-visualization in a Molecular Imager® Gel Doc™ XR system. For purification, the PCR products were precipitated with NH_4Ac. These cleaned PCR products were used for sequencing reactions, using the primer N18L18 (forward primer: 5'-AAGTCGTAACAAGG) located in 18S rDNA. Each sequencing reaction contained 1.5 µl of 5X sequencing buffer, 0.5 µl of N18L18 primer, 1 µl of BigDye® Terminator Ready Reaction Mix, 2 µl of PCR product and 5 µl Milli-Q deionized water. The sequencing reactions were programmed as follows: heating at 96°C for 3 min; 25 cycles of 96°C for 30 s, 50°C for 15 s, and 60°C for 4 min; and an indefinite hold at 4°C. The products of the sequencing reactions were purified by EDTA precipitation. The entire ITS region could be successfully sequenced for 13 *L. bolanderi* individuals from 12 populations (six diploids from six populations, five tetraploid individuals from four populations, and two hexaploid individuals from two populations), for four *L. cymbalaria* individuals from one population, and for one *L. heterophyllum* individual (SI Appendix, Table S1). The ITS2 subregion could be successfully sequenced for 29 more *L. bolanderi* individuals so that we had the ITS sequence of a total of 42 *L. bolanderi* individuals from 18 populations (SI Appendix, Table S1).

The chromatograms of these sequences were imported into Geneious Prime® version 2020.1.2 and trimmed, using the function "trim ends" with the error probability limit set to 0.03 (9). We addressed whether there was evidence for ITS variants that were absent in polyploid *L. bolanderi* individuals using the "Find Heterozygotes" plug-in in Geneious Prime® to identify polymorphisms in the sequences of polyploids. Peak similarity was set to 75% for the sequences of diploids (cf. 10) and to 50% for the sequences of polyploids (cf. 11) to take into account possible double peaks due to heterozygosity or overlapping sequences from different genomes (i.e. allopolyploids). In 33% of the diploid individuals, a highly heterozygous sequence was found after position 91 in the ITS2 region, indicating a 6-bp deletion (CCATTT) after that position. Seven individuals from five populations were heterozygous for the 6-bp-long deletion in the ITS2 subregion; these were both individuals from CHM, both individuals from DIN, one of the two individuals from ELP, the only individual from MXN, and the only individual from SMR. For further analyses, the ITS2 sequences of these heterozygous individuals were split into the 6-bp longer variant A without the deletion, which was identical to the homozygote, diploid *L. bolanderi* sequences, and into the variant B with the deletion, into which a 6-bp gap was manually inserted. We addressed whether ITS variants from other species than *L. bolanderi* could be detected in the samples by also downloading the four diploid and the three polyploid *L. bolanderi* sequences, the 26 sequences of the nine other species in the genus *Lithophragma*, and the sequences of the 11 outgroup species belonging to four closely related saxifragaceous genera sequenced by Kuzoff et al. (8) from GenBank (12). All sequences were trimmed to a maximum common length of 639 bp including the whole ITS and 5.8s regions.

We performed two analyses to disentangle the origin of the allopolyploids. First, we conducted an initial exploratory analysis of the phylogenetic relationships by treating all polyploids as homozygous for the dominating ITS variant. For this, the Geneious Prime®'s default reads of the chromatogram peaks of the polyploids' sequences were used and treated as diploid sequences. Multiple alignment was carried out with Clustal Omega, grouping sequences by similarity, and using fast clustering (mBed algorithm). Phylogenetic trees were built with Geneious Tree Builder, using the Tamura-Nei model and the Neighbor-Joining method. A total of 67 sequences were aligned, and pairwise distances were obtained from the multiple alignment. A consensus neighbor-joining tree was generated using 100 bootstrap replicas, a support threshold set to 50%, and *Mitella yoshinagae* as outgroup. With this approach, we found that ITS sequences of polyploid individuals aligned together with sequences of *L. glabrum* (SI Appendix, Fig. S3). Visual inspection of the chromatograms revealed high heterozygosity, which suggested the presence of multiple ITS variants at some loci. Thus, second, we evaluated whether polyploid genetic composition was consistent with an allopolyploid origin by realigning all diploid and polyploid *L. bolanderi* ITS sequences obtained in this study with the ITS sequences of *L. glabrum* from Kuzoff et al. (8) and then identifying all loci with fixed differences

between diploid *L. bolanderi* and *L. glabrum*. At these loci and three positions up- and downstream thereof, we manually aligned both chromatogram peaks of the polyploids' sequences in Geneious Prime® and examined whether both *L. bolanderi*- and *L. glabrum*-specific ITS variants were present at these loci (see, SI Appendix, Table S2).

S3.2 Genome assembly, repeat discovery and gene annotation. An individual plant from the SMR population (plant ID: 11509A; for population details, see SI Appendix, Table S1) was used to produce Sequel data for genome assembly with an average coverage of 72X. Genome assembly was performed using HGAP4 in SMRTlink version 8.0.0.79519. The assembly pipeline included a polishing step using Quiver. For error correction, reads were piled up and corrected based on the consensus to preserve heterozygosity. The assembly consists of 14,056 contigs with a GC level of 37.1%. Genome completeness was assessed using BUSCO (13) and the Embryophyta gene set, revealing a BUSCO score of 92.7%. A de novo repeat identification was performed with RepeatModeler (1.0.8) (14), and the repeat library by RepeatMasker (4.0.7) (15) was used to mask the repetitive elements in the assembly, which resulted in ~ 415 Mb (52.69 %) of the genome was masked. The genome assembly was annotated using the BRAKER2 pipeline for gene prediction (16–24) using protein hints from Viridiplantae, which resulted in a protein set with a BUSCO score of 90%. Initially, the total number of genes was 53,590, containing 14,047 single-exon genes. We removed single-exon genes and retained transcripts supported by extrinsic evidence using TSEBRA (19). This resulted in a total number of 18,120 genes.

S3.3 Samples, sample preparation, and sequencing for whole-genome data. A 0.25-0.50 cm^3 sized (piece of) root bulbil for each *L. bolanderi* individual was placed into a plastic pot filled with potting soil and slightly covered with soil. We used (pieces of) root bulbils collected from the common-garden plants, and, thus, the plants grown for the genomic analyses were genetically identical to the plants used for the ploidy-level analysis and floral morphology measurements. The growing procedure was the same as for the greenhouse common-garden plants (see SI Appendix, Supporting Text S1) except for the following difference. Pots with freshly planted root bulbils were kept in a small greenhouse room at a 14 h light/10 h dark rhythm with a 165 µmol m^{-2} s^{-1} light intensity during the light phase and at approximately 15°C. When the plants were well established and leaves were fully developed but still fresh, we collected and dehydrated leaf material as described for the plants used for ITS sequencing (SI Appendix, Supporting Text S3.1). Leaf material of the *L. glabrum* plants was collected from the field at POR in 2011 (SI, Appendix, Table S1), dehydrated using dry-rite, and stored dry on dry-rite until extraction.

We extracted DNA from the dehydrated leaf material of *L. bolanderi* and *L. glabrum* using a DNeasy® Plant Mini Kit (QIAGEN®) and the standard protocol provided by the company for the procedure using the TissuLyser and lyophilized (in our case silica-gel-dried) leaf material except for the following changes. The leaf material was grinded twice at 20 Hz for 1.5 min instead of once at 30 Hz for 1 min to avoid breakage of the tubes. After centrifugation, Qiagen Elution Buffer (Qiagen) instead of the AE Buffer was pipetted onto the DNeasy membrane and 60 µl elution buffer were used instead of 100 µl to achieve higher DNA concentrations. This was repeated once after the first round of incubation and centrifugation.

The DNA samples were sent to SciLifeLab in Solna, Sweden (https://www.scilifelab.se/), where they were sequenced through Illumina sequencing on NovaSeq6000 with a 151 read length using NovaSeqXp workflow in S4 mode flowcell and Illumina TruSeq PCR-free with the 350 bp insert size library method.

S3.4 Mapping, variant calling and filtering. We implemented a snakemake (25) workflow for mapping and variant calling accessible in https://github.com/Homap/litho_polyploids/blob/main/workflow/Snakefile. Briefly, reads were trimmed with fastp (26) and then mapped to the *L. bolanderi* assembly with bwa version 0.7.17-r1188 (27). Variant calling was performed with GATK version 4.6 (28). GATK HaplotypeCaller was run individually on each sample with the corresponding ploidy (two for diploids, four for tetraploids, and six for hexaploids) to generate GVCF output. The GVCF files for all samples were imported to a GenomicsDB datastore followed by genotyping GATK GenotypeGVCFs (28, 29) to produce a final raw mixed-ploidy variant call set. Several filtering steps were performed on the raw call set to obtain the final call set of high quality. The mix-ploidy VCF presents challenges in terms of common tools that can be used to filter the data and produce the input files for downstream analyses. In case available tools could not be used, we wrote custom scripts available in https://github.com/Homap/litho_polyploids/tree/main/workflow/scripts. Biallelic single nucleotide

polymorphisms (SNPs) were selected with bcftools (30) and filtered with GATK VariantFiltration using best practice optionsQUAL < 30, QualByDepth (QD) < 5.0, RMSMappingQuality (MQ) < 40.0, MappingQualityRankSumTest (MQRankSum) < -12.5, FisherStrand (FS) > 60.0, ReadPosRankSumTest < -8.0, and StrandOddsRatio (SOR) > 3.0. We removed variants with two times the median of the total depth as the maximum depth and half of it as the minimum. We pruned SNPs with no missing data for linkage disequilibrium using bcftools (30). The total number of biallelic SNPs that passed the filtering was 16,751,857. After removing SNPs with missing data, 2,437,962 SNPS remained. After linkage pruning, we had a total of 374,923 SNPs, which we used for downstream analyses.

S3.5 Analyses of population structure and admixture. A global picture of patterns of polyploid population structure and admixture was obtained by conducting a principal component analysis (PCA) and by using STRUCTURE (31). We used STRUCTURE since it has been shown to be more robust in analyses of mixed-ploidy populations (32). We thinned the VCF file for every 15 SNPs to have a computationally manageable dataset with a total of 21,667 SNPs (all non-coding SNPs) genome wide. We conducted the PCA using the *glPca* function in the *adegenet* R package (33). A summary of the eigenvalues and the variance percentage of principal components (PCs) is shown in SI Appendix, Table S6. As STRUCTURE accepts only uniform ploidy as input, with one row per each ploidy, we added four and two rows of missing data indicated by -9 for diploid and tetraploid samples, respectively, making them pseudo-hexaploid. STRUCTURE was run by using the admixture model with correlated allele frequencies and for K-values 2-10 with ten replicates with a burn-in period of 20,000 and 100,000 Markov Chain Monte Carlo (MCMC) replicates. We plotted the ΔK using the Evanno method (34) and determined K = 3 to be most likely value for K (SI Appendix, Fig. S20).

S3.6 Identification of phylogenetic tree and gene flow events using TreeMix. We estimated population splits and migration using TreeMix (35, 36) with the total number of SNPs (325,002) in blocks of 50 SNPs (k = 50) to build a tree model of the populations and then subsequently added 1 to 10 migration events between populations with 30 replicates of each migration event. We used the program OptM (37) to calculate Δm and to find the most likely number of migration events. The latter was m = 5 edges (SI Appendix, Fig. S21).

S3.7 Formal testing of the mode of polyploidization (i.e., auto- vs. allopolyploidization). We used the software GRAMPA (38), to identify the best fitting tree, including the possibility of multimapping “MUL-trees” enabling different alleles to have different evolutionary origins and hence to assess whether allo- or autoploidization produced the most parsimonious tree. We generated the input gene trees for GRAMPA by phasing the SNPs per individual using WhatsHap, a read-based phasing software (39). This led to the generation of two haplotypes for diploids, four for tetraploids and six for hexaploids. The gene trees used in GRAMPA must be rooted and bifurcating. We chose one of the haplotypes in *L. glabrum* as the outgroup and used IQ-TREE 2 (40) to build gene trees. Gene trees were then resolved for polytomies using the R package *ape* (41). A total of 46 gene trees were given as input of GRAMPA. For the species tree, we used the tree produced by TreeMix when m = 0. The likelihood was used to assess which tree was best, and all trees with equal scores are presented (SI Appendix, Fig. S7).

S3.8 Chloroplast DNA assembly and analysis of variation and structure. In addition to the whole nuclear genome, we assembled the complete chloroplast DNA (cpDNA) assembly consisting of 155,384 bp including both inverted repeat sequences. We annotated the cpDNA genomes using GeSeq (42) with Embryophyta cpDNA assemblies as evidence (SI Appendix, Figure S22). Variant calling for cpDNA was done as described in the mapping, variant calling and filtering section above (SI Appendix, Supporting Text S3.4), with the exception that in GATK HaplotypeCaller, the ploidy was set to one and genotyping was done per sample. We then used vcftools (43) to call consensus fasta sequences for each sample. A cpDNA tree was built using IQ-TREE 2 (40) with GTR+G model and 1000 bootstraps. An Integer NJ Net haplotype network was built using POPART (44, 45).

S3.9 Allele frequency spectra. We estimated the site frequency spectra separately for tetraploid and hexaploid *L. bolanderi* individuals by merging the six tetraploid and four hexaploid individuals, respectively, resulting in an equivalent of 12 diploid genomes for each of the two polyploid cytotypes. We used dadi v2.2.0 (46) to retrieve allele frequency spectra for the tetraploids and hexaploids, using the projection with the maximum possible number of bins (24 for both categories). While sample sizes

are relatively small for retrieving allele frequency spectra, and we pool across sampling sites, we expect the signal from allopolyploidization to be strong enough to be distinguishable.

S3.10 K-mer analysis. We characterized genome properties and inferred ploidy levels by conducting k-mer analysis with FastK (v1.1) and GenomeScope2 (v2.0.1) (https://github.com/thegenemyers/FASTK/releases) (47). Raw Illumina sequencing reads after quality filtering were passed through FastK to count all 21-mers in the entire dataset. The k-mer frequency histogram generated was then subjected to an analysis in GenomeScope2, which attempts to parametrize the k-mer distribution into estimates of genome size, heterozygosity, and repeat content, while at the same time inferring the most likely ploidy model. Allele pairing behavior and ploidy inferences verification was assesses using Smudgeplot (v0.4) (47). A Smudgeplot visualizes k-mer pair coverage, which discriminates between signals of diploid, autopolyploid, and allopolyploid origin. Together, these tools gave complementary information regarding the genome structure and helped to test potential polyploid origin.

S3.11 Distribution of per individual reference allele proportion. We assessed whether tetraploid (4x) and hexaploid (6x) individuals exhibit patterns consistent with autopolyploidy or allopolyploidy by first identifying SNP sites where diploid *L. bolanderi* (represented by BAM, MIN, and MXN samples) showed a fixed difference from *L. glabrum*. That is, where the former had only the reference allele and the latter had only the alternate allele. For each such site (of a total of 2162 sites), we calculated the proportion of *L. bolanderi*-type alleles in every polyploid individual by dividing the reference allele count by the total allele count. These per-individual, per-site proportions were plotted as bar plots per genotype to visualize the distribution of *L. bolanderi*-type allele.

S4 Floral morphology measurements
In total, 15 measures were taken from each flower. Flower diameter (FlDi, 1 in Fig. 2A) and corolla-opening diameter (CorOpDi, 2) were measured to the nearest 0.01 mm on fresh flowers using a digital caliper. Then, the flowers were stored in 70% EtOH in a 1.5-ml Eppendorf tube until further processing. For measures 3-15 (Fig. 2A), the flowers stored in 70% EtOH were dissected using micro scissors, forceps, and a scalpel, photographed on a piece of millimeter paper for scale using a handheld digital microscope (Celestron®, Celestron 5 MP Handheld Digital Microscope Pro), and quantified based on four to five photos in the image processing program ImageJ (https://imagej.net/Fiji) (Fig. 2A). The millimeter paper in the background of the photos served as a scale. Petal length (PetLen, 3) and petal width (PetWid, 4) were measured for two randomly selected, non-adjacent petals. Depending on the size of the petals, one photo with both petals was taken or two photos with one of the petals each. A few petals were too large to fit in a photo and were measured with a digital caliper. Measurements with the digital caliper and ImageJ measurements were highly comparable as verified by measuring PetLen and PetWid for a few individuals both with the digital caliper and in ImageJ. Corolla gap (CorGap, 5), overall flower length (FlLen, 6), internal flower length (IntFlLen, 7), ovary depth (OvDep, 8; calculated as FlLen – IntFlLen), floral flair (FlFlair, 9), long floral angle (LgAng, 10; measured for both directions), floral width (FlWid, 11), nectary disc length (NectLen, 12), diameter of the largest stigmatic lobe (LobeDi, 13), outer distances between lobes (LobeC, 14a-c), and pistil height above the nectary disk (StyleHt, 15). As this resulted in two or more measures per flower for PetLen (3), PetWid (4), LgAng (10), and LobeC (14), and as they were, in general, strongly correlated (SI Appendix, Table S7), mean values were calculated for 3, 4, and 10 and all measures for 14 were summed up to a stigma circumference (LobeC) for statistical analyses.

As the flowers were apportioned among three persons to conduct the measurements, we took the following measures to guarantee repeatability. One person remeasured a subset of the flowers measured by the other two persons. The measurements conducted by different persons were highly correlated (Spearman's rank correlations ρ; PetLen1: 0.99, PetLen2: 0.98, PetWid1: 0.99, PetWid2: 0.99, CorGap: 0.96, FlLen: 0.87, IntFlLen: 0.90, FlFlair: 0.89, LgAng1: 0.87, LgAng2: 0.88, FlWid: 0.97, NectLen: 0.96, StyleHt: 0.98), except initially for LobeDi (Spearman's rank correlation $\rho = 0.77$) and for the LobeCs (Spearman's rank correlations ρ; LobeC1: 0.44, LobeC2: 0.64, LobeC3: 0.67), Spearman's rank correlations ρ were < 0.80 due to the difficulty of getting simultaneously a consistent perspective on this three-dimensional structure and all other structures (see 14 in Fig. 2A). For unclear cases, we therefore took another photo focusing on this specific structure, and the same person remeasured measures 13 and 14 on these photos. This one person also visually checked for "outliers" separately for each population and cytotype, double-checked these measures, and remeasured them if necessary. Obviously malformed flowers were excluded from the analyses.

S5 Synthetic polyploidization experiment

S5.1 Sowing and plant growing conditions. The F0 plants were grown in two cohorts, because sample sizes were too low after the first round of the synthetically inducing polyploidization due to a high mortality, most likely caused by the colchicine treatment. The seeds were sown in February and December 2019 for the first and the second cohort, respectively. Across cohorts, we sowed 2414 seeds from 9 diploid seed families from the population KAW with 174-300 seeds per seed family and 660 seeds from 11 tetraploid seed families from the population KAW with 60 seeds per seed family. The sowing and growing conditions were as described for the greenhouse common-garden plants (SI Appendix, Supporting Text S1) with the following exceptions. In the first cohort, the newly sowed pots were kept in a small greenhouse room in the greenhouse facility of Lund University at a 14 h light/10 h dark rhythm with a 165 µmol m^{-2} s^{-1} light intensity during the light phase and at approximately 15°C. In the second cohort, the newly sowed pots were kept in growth chambers (Micro Clima-Series™ Economic Lux Chamber, Snijders Labs, The Netherlands; temperature: 15° C; 14 h light/10 h dark; light intensity during light phase: 165 µmol m^{-2} s^{-1}; air humidity: 60%). We covered the pots with transparent plastic foil to avoid drying out and watered them approximately twice a week. After the colchicine treatment, the seedlings were planted into individual wells (bottom ø: 2.8 cm; top ø: 4 cm; height: 4 cm) of 66-well trays filled with wetted S-jord and kept in the greenhouse (first cohort) or growth chambers (second cohort), under the same conditions as described above. When the seedlings that survived were well established, they were transplanted into 9×9×7-cm plastic pots filled with wetted S-jord potting soil with a few nutrient balls per pot mixed with the potting soil. The plants of the first cohort were, from now on until they finished flowering and dried out, kept in the same small greenhouse room under the conditions described. The plants of the second cohort were kept in the growth chambers under the conditions described until they were well established and then moved to a larger greenhouse (temperature: approximately 15°C; 14 h light/10 h dark; natural and artificial light during light phase), where they were automatically watered every third day.

The F1 plants were grown in two cohorts because sample sizes were too low in the first round due to high mortality, most likely caused by unexpected high temperatures in the greenhouse. The first cohort was sown in March 2021. For colchicine-treated plants as well as for diploid and tetraploid control plants, approximately 20 seeds per crossing were sown in round plastic pots (9×7.5 cm) filled with wetted soil (a substrate similar to S-jord, which we manually mixed and which consisted of 15 parts soil (Einheitserde - BL Bio T Öko torffrei, Einheitserdewerke Werkverband e.V., Sinntal-Altengronau, Germany), one part sand (silica), and two parts perlite (PERLIGRAN ® Extra; Knauf Aquapanel GmbH, Dortmund, Germany)). After approximately two months, up to two seedlings per pot were transplanted into individual plastic pots. The second cohort was sown in December 2021. For the colchicine-treated plants as well as for the diploid and tetraploid control plants, four seeds per crossing were sown into individual pots (4×4×5 cm) of plastic seed trays with 3×4 pots filled with the same wetted substrate as in the first cohort except that another Einheitserde soil was used (Einheitserde CL ED73, Einheitserdewerke Werkverband e.V., Sinntal-Altengronau, Germany).

Starting from approximately two months after sowing, the strongest plants were transferred to larger individual round plastic pots (9×7.5 cm). Across both cohorts, we sowed approximately 880 seeds from 60 crossings with 43 donor plant × receiver plant combinations of the colchicine-treated plants, approximately 610 seeds from 33 crossings with 29 donor plant × receiver plant combinations of the diploid control plants, and approximately 630 seeds from 32 crossings with 32 donor plant × receiver plant combinations of the tetraploid control plants. In both cohorts, seeds were germinated in a climate chamber (Sanyo Growth Cabinet MLR-350T, Sanyo Electric Co. Ltd., Japan; 14 h light at 15° C / 10 h dark at 10° C). In the first cohort, initially the freshly transplanted plants were moved to a greenhouse of the greenhouse facility of the Paris Lodron University of Salzburg (natural light condition augmented with artificial light for 14 h per day; temperature: ≥ 15° C), where they were spot-watered daily. Due to high temperatures for this time of year, the plants did not do well and were placed back into climate chambers (one Sanyo Growth Cabinet MLR-350T, Sanyo Electric Co. Ltd., Japan; two Liebherr UKS 5000, Strauss, Salzburg, Austria) after six to eleven days. Because of that, the plants subsequently transplanted were kept in the climate chambers. The plants of the second cohort were kept all the time in these climate chambers. The temperature and light settings in the climate chambers were the same as described above.

S5.2 Colchicine treatment. We carefully picked diploid seedlings out of the soil using tweezers, rinsed as much soil off the roots as possible in water, and carefully dabbed the seedlings on paper towel to remove as much water as possible. Depending on seedling size, we placed up to 30 seedlings of a seed family into a plastic Petri dish (ø 5 cm). We poured 0.2% colchicine solution (2 mg colchicine powder (97%, Fisher Scientific, Art.-nr.: 10174953) in 1 ml dH_2O) into the Petri dishes until

all seedlings were completely covered with the solution, which corresponded to approximately 5-10 ml depending on seedling size. The seedlings were incubated in the colchicine solution over night for approximately 16 h at room temperature in a fume hood darkened with black covering. Thereafter, we removed the colchicine solution with a 5-ml pipette, rinsed the seedlings three times in DWH_2O by holding the seedlings with a pair of tweezers and swiveling them in three consecutive 100-ml glass bottles filled with DWH_2O, and placed the rinsed seedlings into clean plastic Petri dishes (ø 5cm). We generated the diploid and tetraploid control plants by treating diploid and tetraploid seedling, respectively, as the seedlings of the colchicine-treated plants except that we used DWH_2O instead of the colchicine solution and that we did not rinse them in DWH_2O. Across cohorts, 793 seedlings from 9 diploid seed families with 32-139 seedlings per seed family were treated with the colchicine solution constituting the colchicine-treated plants, 87 seedlings from 9 diploid seed families with 8-10 seedlings per seed family were treated with DWH_2O constituting the diploid control plants, and 149 seedlings from 11 tetraploid seed families with 6-15 seedlings per seed family were treated with DWH_2O constituting the tetraploid control plants.

S5.3 Ploidy-level analyses using flow cytometry. When plants were well established and leaves fully developed but still fresh, leaf material was collected and sent to Plant Cytometry Service as described for the leaf material collected in the natural populations (SI Appendix, Supporting Text S2.2). For the F0 plants, ploidy level was assessed for all plants ($n_{\text{colchicine-treated plants}}$ = 28, $n_{\text{diploid control plants}}$ = 25, $n_{\text{tetraploid control plants}}$ = 113). For the F1 plants, ploidy level was assessed for all plants of the colchicine-treated group (*n* = 54) and of a subset of the plants of the diploid (*n* = 10) and the tetraploid (*n* = 9) control groups.

S5.4 Hand-pollination crossings. Hand pollinations were conducted by touching and completely covering all stigmatic lobes of the receiver plant with pollen of one to several anthers of the donor plant picking and holding the anthers with tweezers. Each hand-pollinated flower was labelled with a small tag. Across both cohorts, we conducted 96 hand pollinations on 16 individuals from six seed families of the colchicine-treated plants using one to five individuals per seed family and conducting one to 11 crosses per individual, 54 hand pollinations on 10 individuals from five seed families of the diploid control plants using one to five individuals per seed family and conducting four to eight crosses per individual, and 116 hand pollinations on 15 individuals from nine seed families of the tetraploid control plants using one to four individuals per seed family and conducting two to 18 crosses per individual. When capsules were mature after approximately 2 weeks (i.e., when the three carpels start to open at the top, and, in case they bore seeds, dark brown/black seeds could be seen through the opening slits but just before they fell out), we picked the entire capsules and stored them in individual 5.5×8-cm glassine bags.

S5.5 Floral-morphology measurements. Flowers were collected and measured as described for the flowers from the common garden (SI Appendix, Supporting Text S4) with the following exceptions. The dissected flowers were photographed using a digital microscope (Leica DVM6 with a Planapo FOV 12.55 magnification objective, Leica Mikrosystems). As a scale, a piece of mm paper was included in each photo. Based on these photos, floral morphology traits were measured using the Leica Application Suite software (LAS X, version 3.0, Leica Mikrosystems). We measured the same floral morphology traits in the same way as for the flowers of the large-scale greenhouse common garden (see Fig. 2A) except that we measured the length and width of all petals of the flower to calculate the mean petal length and mean petal width.

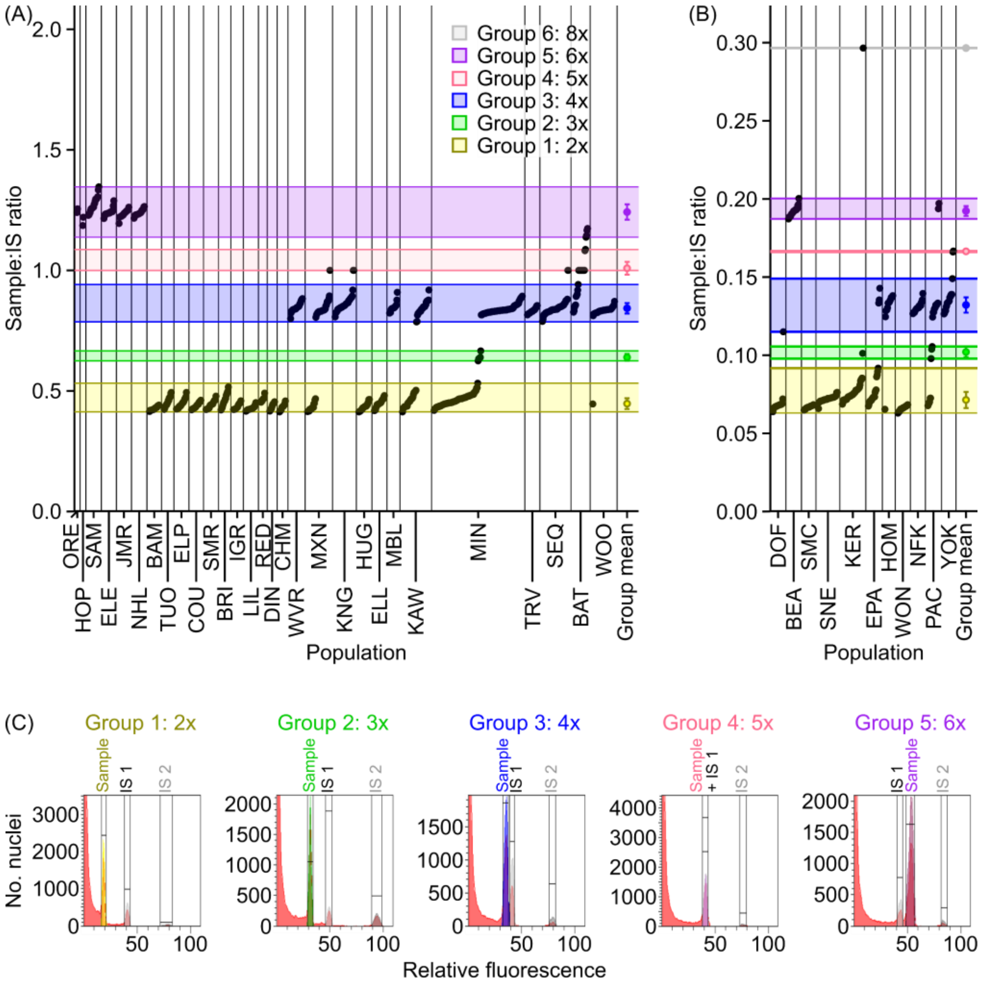

Fig. S1. Sample:IS ratios (IS: internal standard) of 1046 *Lithophragma bolanderi* individuals across 40 populations (**A**, **B**) and relative fluorescence of samples of different ploidy level and of the IS (**C**). (**A**) Samples analyzed by the authors on the BD LSRFortessa^{TM} flow cytometer at BIOVIS at Uppsala University, Sweden, (https://biovis.uu.se/) based on fresh leaf material collected in the common garden with *Brassica napus* as internal standard. (**B**) Samples analyzed by Plant Cytometry Service (https://www.plantcytometry.nl/) based on fresh leaf material collected in natural populations in California with *Allium schoenoprasum* as IS. (**A**, **B**) Discontinuities in sample:IS ratios indicated six different sample:IS ratio groups (group 1-6) across samples. The sample:IS ratios also varied considerably within ploidy groups and populations. In *Lithophragma*, some individuals contain satellite or accessory chromosomes (6), which may contribute to this variation. We used the information of known ploidy levels from karyological counts by Taylor (6) to assign the six sample:IS ratio groups we found to absolute ploidy levels (for details, see SI Appendix, Supporting Text S2.1, S2.2). 2x: diploid, 3x: triploid, 4x: tetraploid, 5x: pentaploid, 6x: hexaploid, 8x: octoploid. Each symbol represents a *L. bolanderi* individual. Individuals are arranged according to sample:IS ratio and according to population. Populations are indicated with their three-letter code (for details, see Fig. 1 and SI Appendix, Table S1) and arranged according to latitude (from north (left) to south (right)). Sample:IS ratio groups and mean ± 1 standard deviation of the sample:IS ratios for each sample:IS ratio group are color-coded according to the assigned ploidy level. (**C**) Representative examples of relative fluorescence histograms for each of the five sample:IS ratio groups in (**A**). The sample- and IS-peaks are gated and color-coded according to the assigned ploidy level or highlighted in grey, respectively. The IS always produced two peaks: a first, main peak (IS 1) and a second, smaller peak (IS 2) with a relative fluorescence twice as high as IS 1. For the calculation of the sample:IS ratio, the IS 1 peak was used. For the sample:IS ratio group 4, which corresponded to the ploidy level 5x, the relative fluorescence of the sample and the IS 1 peak overlapped. The presence of a sample peak was verified by running the sample without IS (not shown here). The samples were individuals from the following populations: the 2x sample from ELL, the 3x sample from MIN, the 4x sample from BAT, the 5x sample from SEQ, and the 6x sample from ELE.

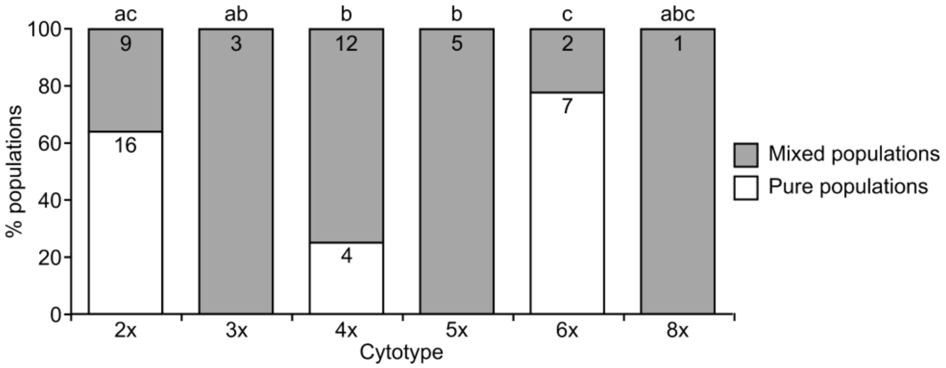

Fig. S2. Percentage of *Lithophragma bolanderi* populations consisting of only one cytotype (pure populations) and of two or more cytotypes (mixed populations). 2x: diploid, 3x: triploid, 4x: tetraploid, 5x: pentaploid, 6x: hexaploid, 8x: octoploid. Numbers within bars indicate the number of populations. Different letters above bars indicate statistically significant differences (Fisher's exact tests: $P < 0.05$).

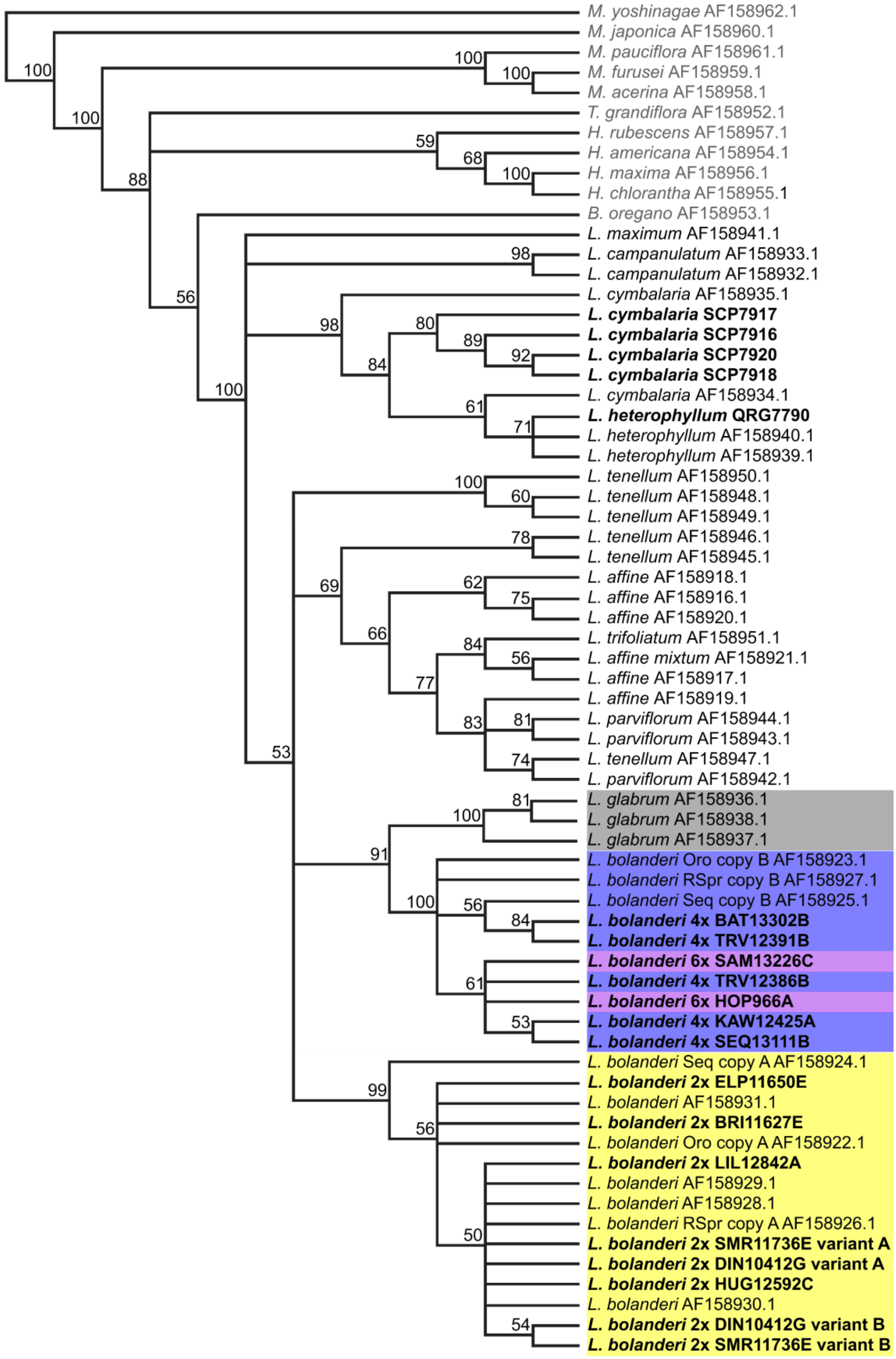

Fig. S3. Consensus tree of the ITS sequences from diploid and polyploid *Lithophragma bolanderi* and 10 other *Lithophragma* (sub)species (in black font) as well as 11 outgroup species belonging to four genera (*Bensoniella*, *Heuchera*, *Mitella*, and *Tellima*; in grey font) obtained by the neighbour joining method. Taxa in bold were sequenced in the present study and taxa in plain text were extracted from Kuzoff et al. (8). Bootstrap values are indicated for each node. The ITS sequences of the polyploids in this study were treated as diploid sequences using the default reads provided by Geneious (for details, see SI Appendix, Supporting Text S3.1). Diploid *L. bolanderi* sequences and copy A *L. bolanderi* sequences from Kuzoff et al. (8) are highlighted in yellow, tetraploid *L. bolanderi* sequences from this study and copy B *L. bolanderi* sequences from Kuzoff et al. (8) are highlighted in blue, hexaploid *L. bolanderi* sequences from this study are highlighted in purple, and *L. glabrum* sequences – the species identified by Kuzoff et al. (8) as the second parental species besides diploid *L. bolanderi* of polyploid *L. bolanderi* – are highlighted in grey. This phylogeny indicates that all polyploid individuals in our study clustered with the three individuals of *L. glabrum* and with the ITS copy type B of the three polyploid *L. bolanderi* individuals sequenced by Kuzoff et al. (8).

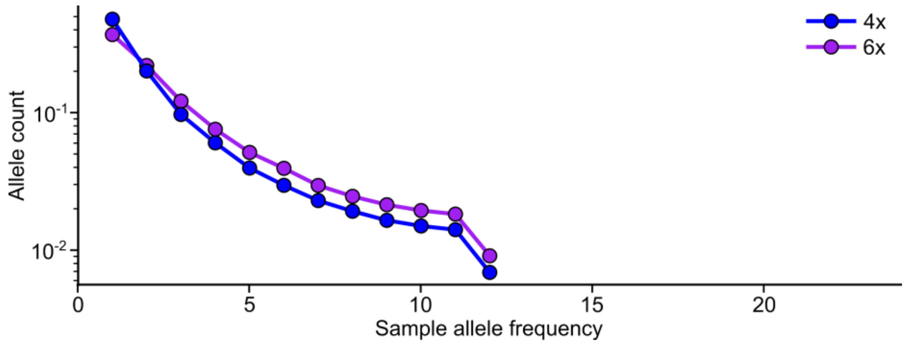

Fig. S4. The site frequency spectrum for the group of sequenced tetraploid (4x; blue) and the group of sequenced hexaploid (6x; purple) individuals of *Lithophragma bolanderi*.

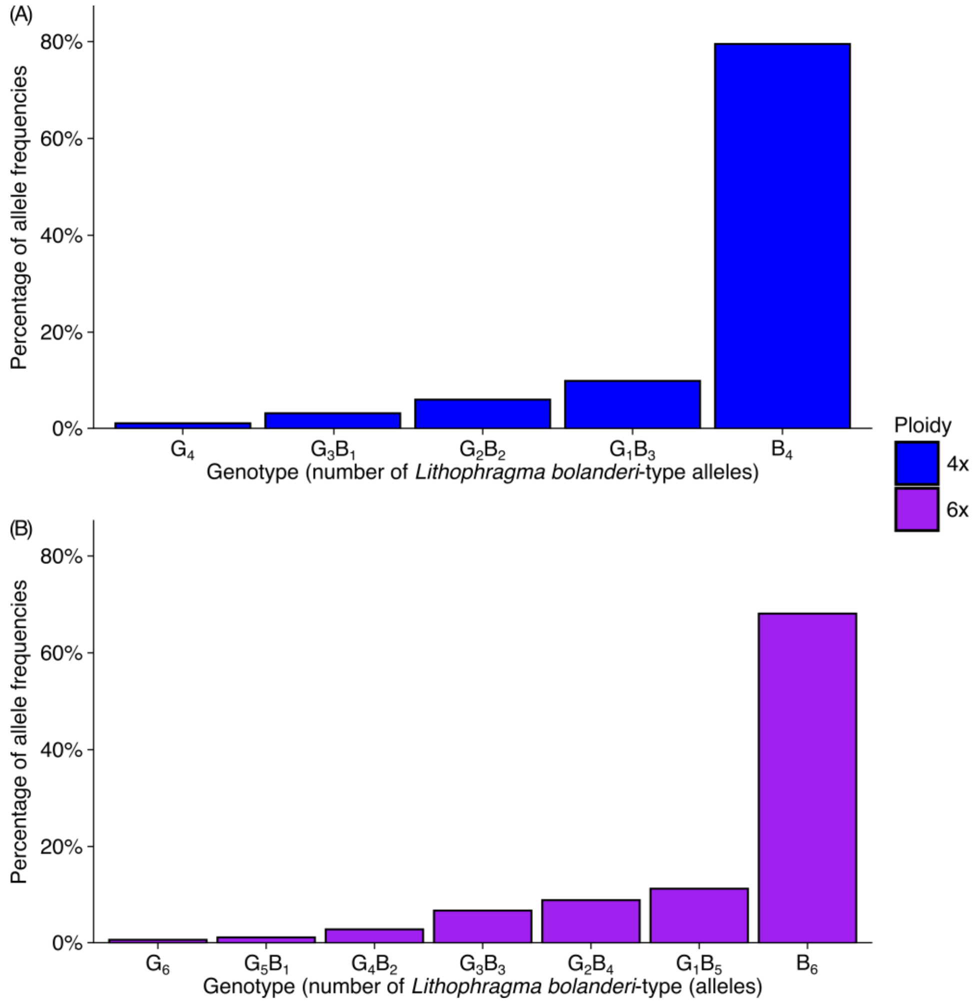

Fig. S5. Distribution of *Lithophragma bolanderi*-type alleles across tetraploids (4x; blue) (**A**) and hexaploids (6x; purple) (**B**). Bar plots show the percentage of SNPs with different proportions of *L. bolanderi*-type alleles across fixed-difference sites between diploid *L. bolanderi* and *L. glabrum*. Each bar represents a genotype class based on the number of *L. bolanderi*-type alleles (B) and *L. glabrum*-type alleles (G). The majority of SNPs in both ploidies showed high proportions of the *L. bolanderi*-type allele, especially in 4x individuals.

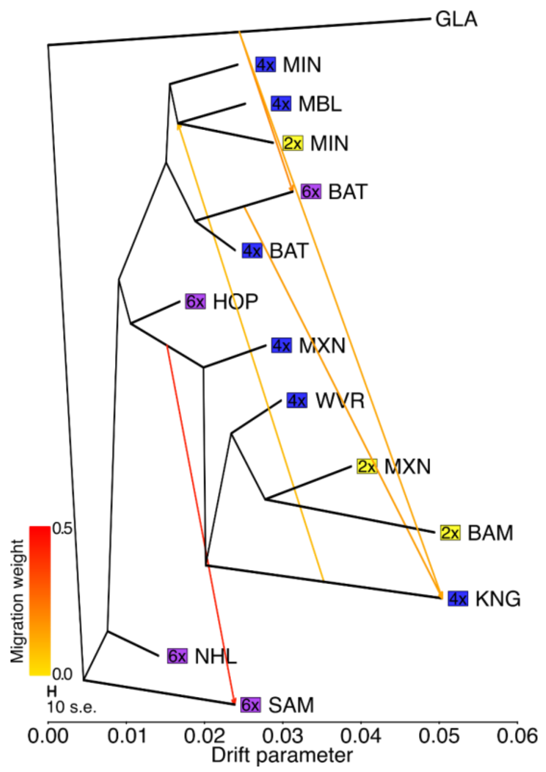

Fig. S6. The tree structure inferred by TreeMix for the outgroup *Lithophragma glabrum* (GLA), three diploid *L. bolanderi* populations, six tetraploid individuals and four hexaploid individuals. The migration edges are colored according to their weight and the individuals/populations of *L. bolanderi* according to cytotype (diploid; 2x, yellow; tetraploid: 4x, blue; hexaploid: 6x, purple). The scale bar indicates ten times the average standard error of the values in the covariance matrix.

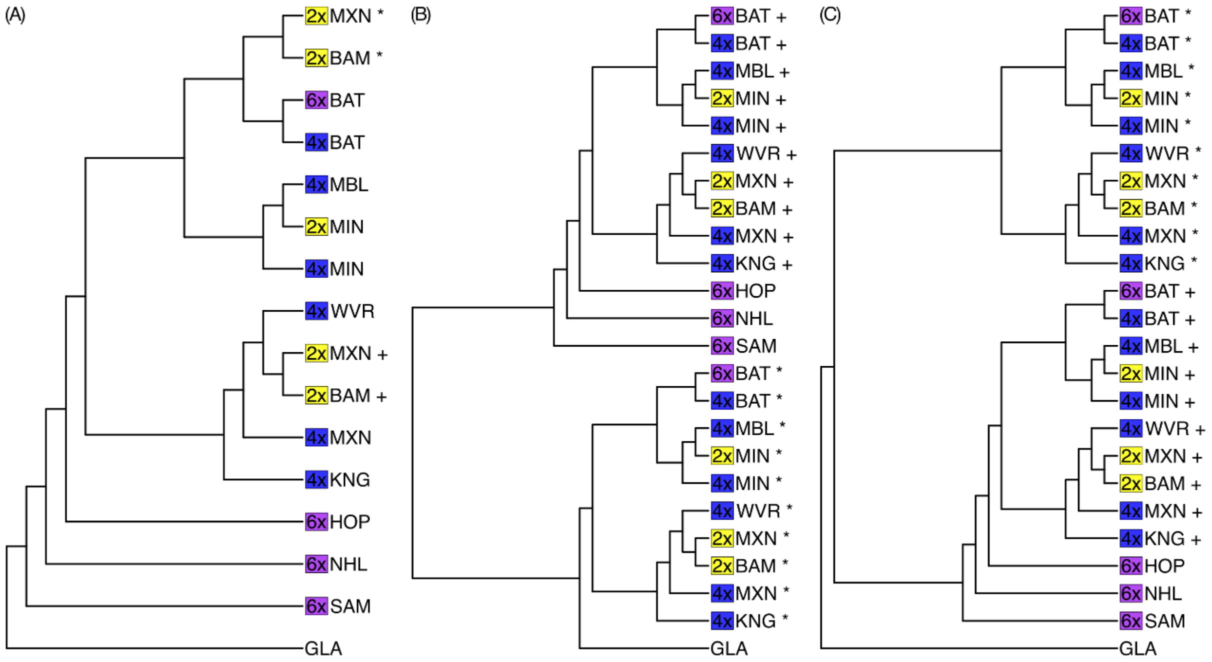

Fig. S7. The three most parsimonious trees generated by GRAMPA. All had the same score of 317. Individuals/populations of *Lithophragma bolanderi* are colored according to cytotype (diploid; 2x, yellow; tetraploid: 4x, blue; hexaploid: 6x, purple). GLA: *L. glabrum*.

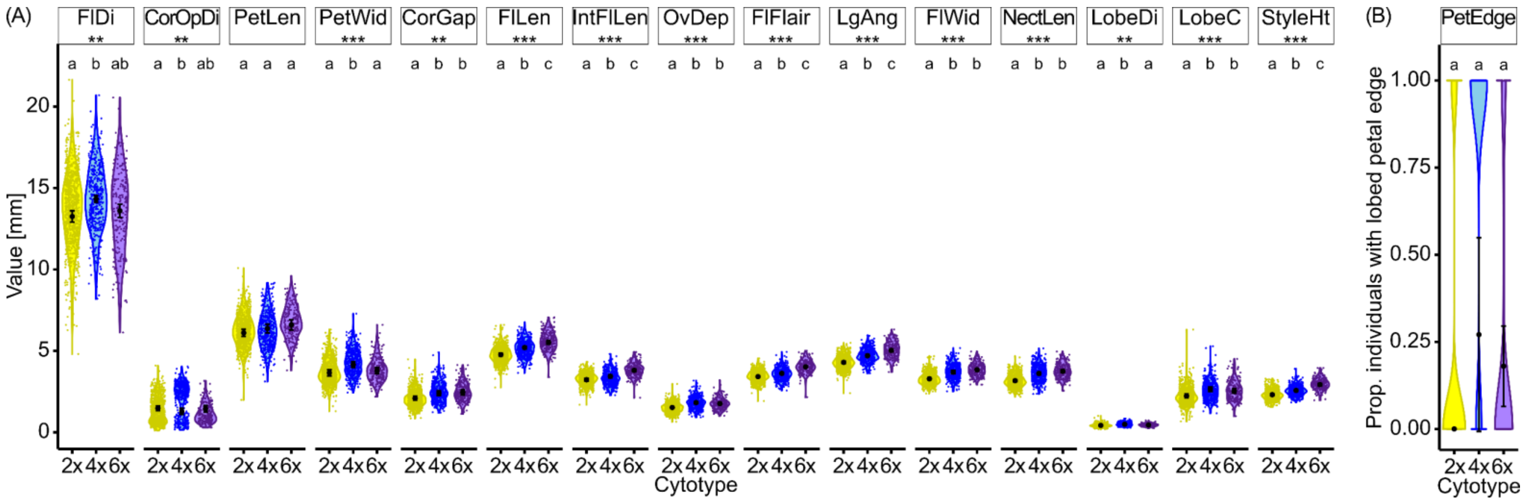

Fig. S8. Variation in individual floral morphology traits (for full names, see Fig. 2) in *Lithophragma bolanderi*. (**A**) Differences in the 15 numeric floral morphology traits among the three dominant cytotypes: diploids (2x), tetraploids (4x), and hexaploids (6x). (**B**) Differences in the categorical floral trait shape of the petal edge (PetEdge; see Fig. 2B), which is either lobed or whole, shown as differences in the proportion of individuals with lobed petal edges among the three dominant cytotypes. Violin plots and points representing individuals are color-coded according to cytotype. Black points and error bars represent means ± 1 standard error as estimated from linear mixed-effect models. Significance levels of the linear mixed-effect models are indicated next to the floral traits: “” $P > 0.5$, “*” $P < 0.05$, “**” $P < 0.01$, “***” $P < 0.001$. Different lowercase letters at the top of the graphs indicate significant ($P < 0.05$) differences in pairwise post hoc comparisons of cytotypes.

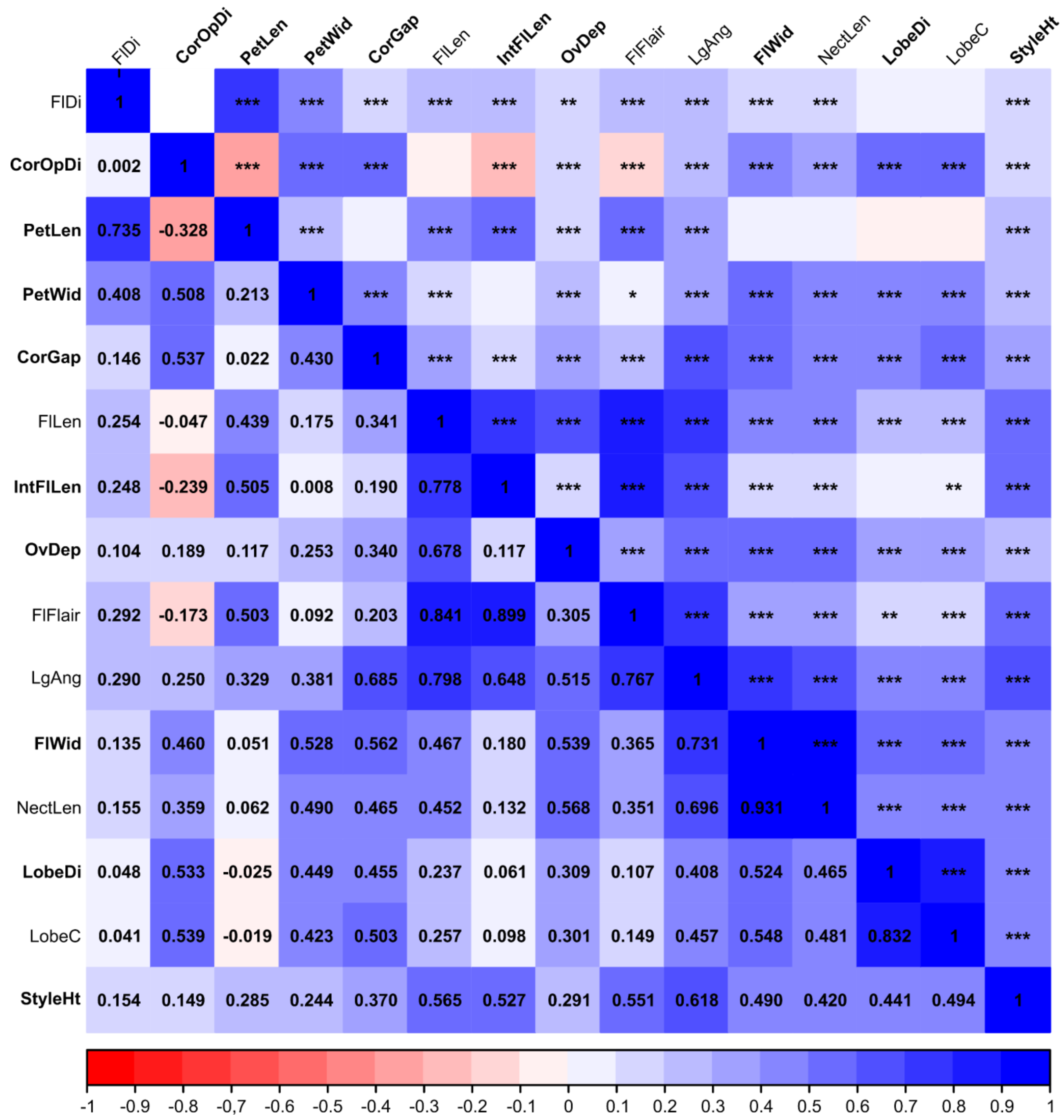

Fig. S9. Pairwise correlations of all floral traits (for full names, see Fig. 2) across all *Lithophragma bolanderi* individuals displayed as heatmap. Colors indicate the strength (the darker the shade the stronger the correlation) and direction (red shades: negative correlations, blue shades: positive correlations) of the correlations. In the lower half of the heatmap, the Spearman's rank correlation coefficients (Spearman's ρ) are indicated. They were computed using the function *rcorr* in the R package *Hmisc* (version 4.4-2 (48)). In the upper half of the heatmap, significance levels for the correlations are given (“” $P > 0.05$, “*” $P < 0.05$, “**” $P < 0.01$, “***” $P < 0.001$). *P*-values were holm-adjusted, using the function *p.adjust* implemented in the *stats* package of R (version 4.0.3 (49)). Names of traits with all pairwise Spearman's $\rho < 0.7$, which were included in multivariate statistical analyses, are in bold.

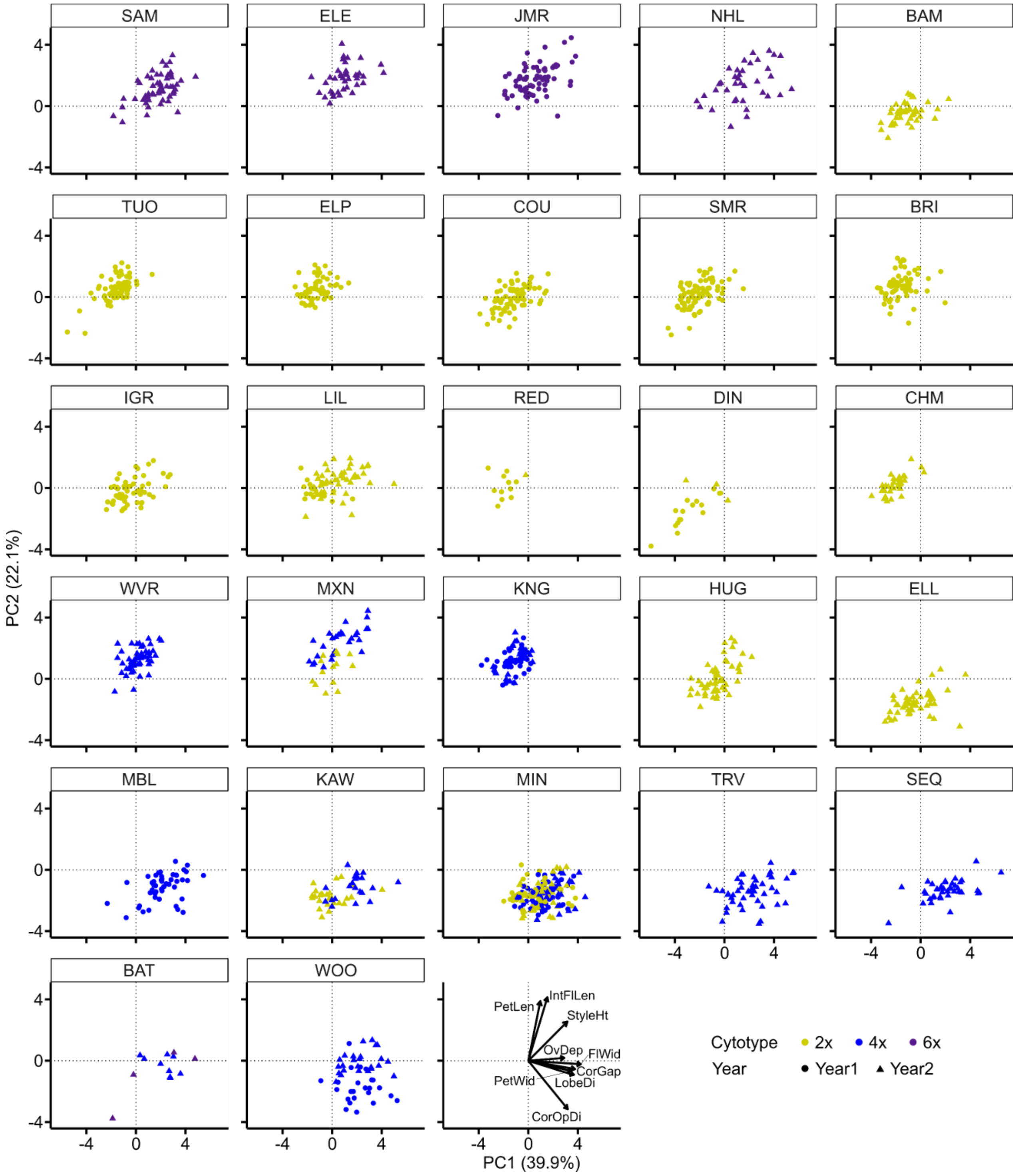

Fig. S10. Differentiation in floral morphology in *Lithophragma bolanderi* among the three dominant cytotypes (2x: diploids, 4x: tetraploids, 6x: hexaploids), populations, and the year when plants were grown according to principal component 1 (PC1) and PC2 (SI Appendix, Table S8). Symbols represent individuals and are color-coded according to cytotype and shaped according to the year when the plants were grown (Year). For better visibility, each population is shown in an individual plot (for details on populations, see Fig. 1A and SI Appendix, Table S1). Populations are ordered according to latitude from north (top left) to south (bottom right). The variance explained by the PCs is given in brackets. Traits loading on the PCs are shown in the last plot (see also SI Appendix, Table S8).

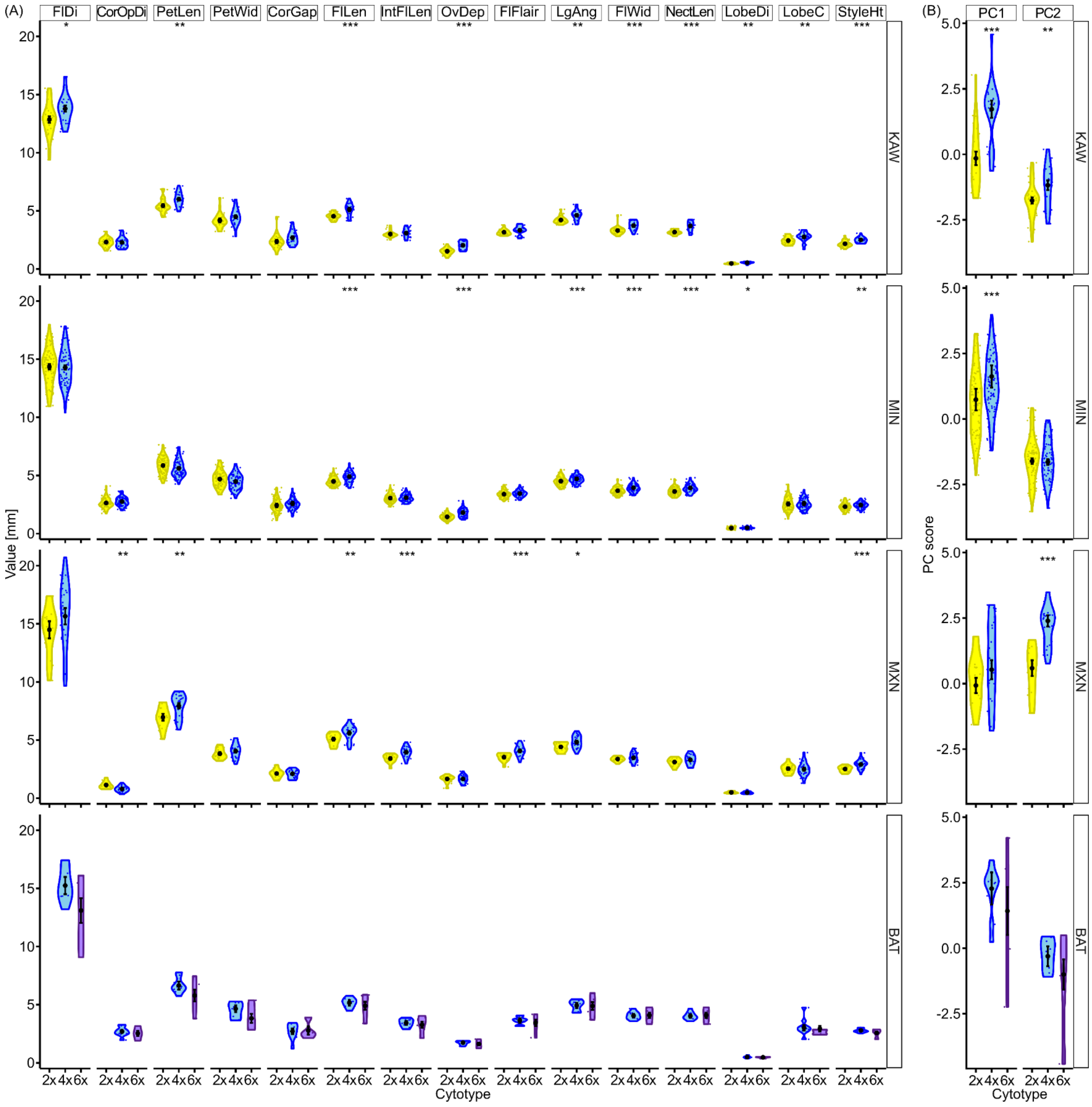

Fig. S11. Differences in the 15 numeric floral morphology traits (**A**; for full names, see Fig. 2) and in the first two principal components (PCs) (**B**; for details see SI Appendix, Table S8) among cytotypes (2x: diploid, 4x: tetraploid, 6x: hexaploid) in the three 2x-4x mixed populations KAW, MIN, and MXN and in the 4x-6x mixed population of *Lithophragma bolanderi* (for details on populations, see Fig. 1A and SI Appendix, Table S1). Violin plots and points representing individuals color-coded according to ploidy level. Black points and error bars represent means ± 1 standard error as estimated from linear mixed-effect models (LMMs). Significance levels of the LMMs are indicated at the top of each graph: “” $P > 0.5$, “*” $P < 0.05$, “**” $P < 0.01$, “***” $P < 0.001$.

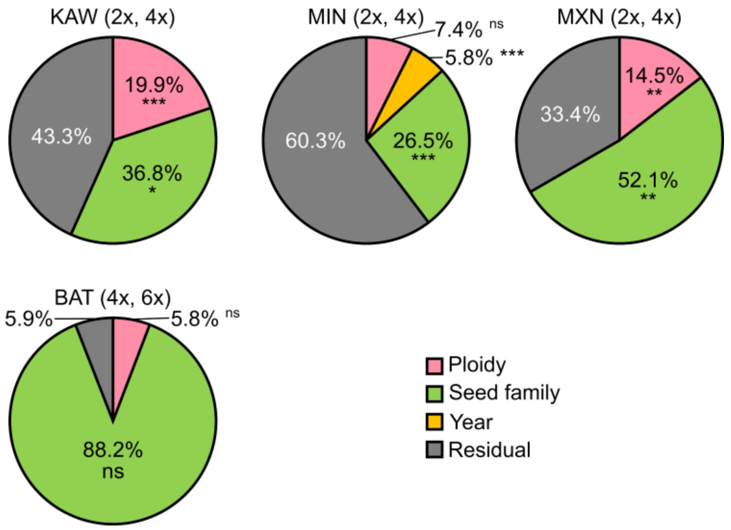

Fig. S12. The percentage of variance in floral morphology explained by the factors cytotype (Ploidy), the year when the plants were grown (Year; applied only for the population MIN), and seed family estimated from permutational analyses of variance in the three *Lithophragma bolanderi* populations KAW, MIN, and MXN with diploid (2x) and tetraploid (4x) plants and in the population BAT with tetraploid and hexaploid (6x) plants. Significance levels for these factors are indicated: "ns" *P* > 0.5, "*" *P* < 0.05, "**" *P* < 0.01, "***" *P* < 0.001.

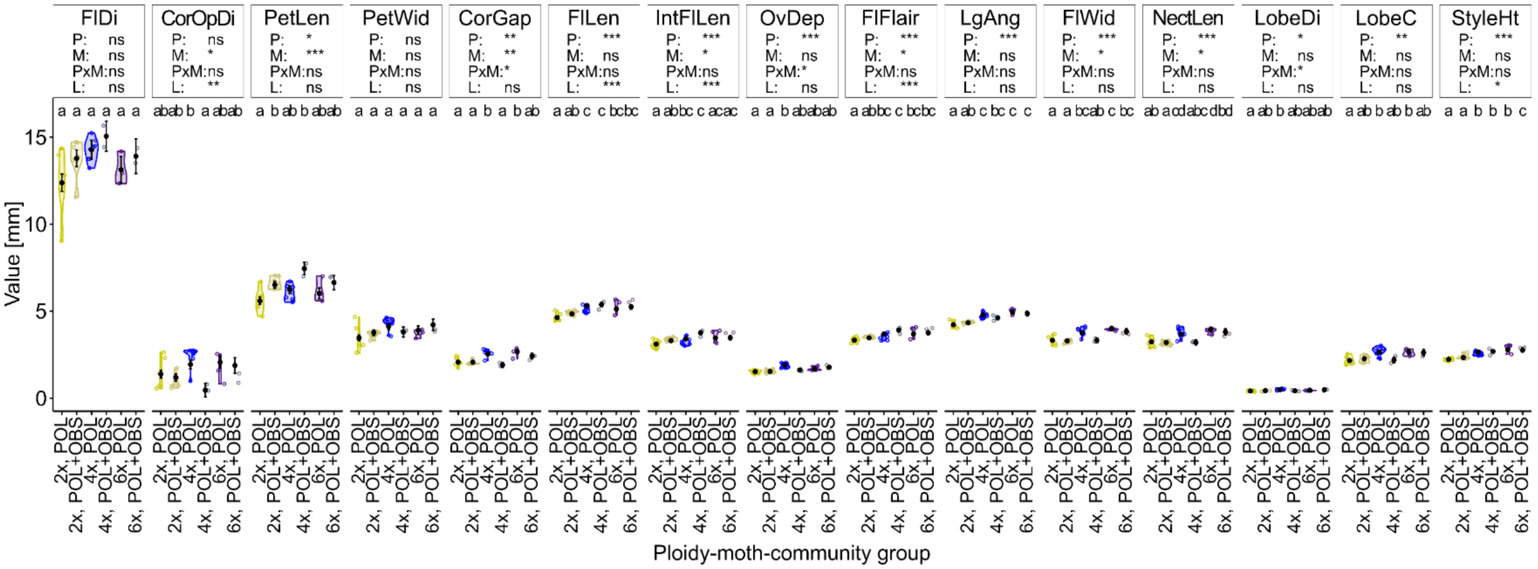

Fig. S13. Differences in the population means computed using linear mixed-effect models (LMMs) (for details, see SI Appendix, Table S17) of the 15 numeric floral morphology traits (for full names, see Fig. 2) among the three dominant cytotypes (2x: diploids, 4x: tetraploids, 6x: hexaploids), between the two different *Greya* moth communities (POL: populations with *G. politella* only, POL+OBS: populations with *G. politella* and *G. obscura*), and with latitude as a covariate. Violin plots and points representing individuals are color-coded according to cytotype and *Greya* moth community. Black points and error bars represent means ± 1 standard error as estimated from linear models (LMs). Different lowercase letters at the top of the graphs indicate significant ($P < 0.05$) differences in pairwise post hoc comparisons among the six groups of these LMs. Significance levels of LMs for cytotype (P), *Greya* moth community (M), the cytotype × moth-community interaction (PxM), and latitude (L) are indicated directly underneath the floral trait names: “ns” $P > 0.5$, “*” $P < 0.05$, “**” $P < 0.01$, “***” $P < 0.001$.

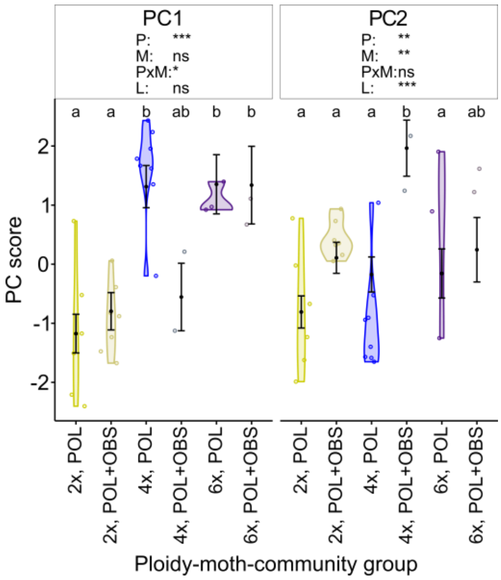

Fig. S14. Differences in the population means computed using linear mixed-effect models (LMMs) (for details, see SI Appendix, Table S174) of principal component (PC) scores of PC1 and PC2 (for details on trait loadings on the PCs, see SI Appendix, Table S8; SI Appendix, Fig. S10) among the three dominant cytotypes (2x: diploids, 4x: tetraploids, 6x: hexaploids), between the two different *Greya* moth communities (POL: populations with *G. politella* only, POL+OBS: populations with *G. politella* and *G. obscura*), and with latitude as a covariate. Violin plots and points representing individuals are color-coded according to cytotype and *Greya* moth community. Black points and error bars represent means ± 1 standard error as estimated from linear models (LMs). Different lowercase letters at the top of the graphs indicate significant ($P < 0.05$) differences in pairwise post hoc comparisons among the six groups of these LMs. Significance levels of LMs for cytotype (P), *Greya* moth community (M), the cytotype × moth-community interaction (PxM), and latitude (L) are indicated directly underneath the floral trait names: “ns” $P > 0.5$, “*” $P < 0.05$, “**” $P < 0.01$, “***” $P < 0.001$.

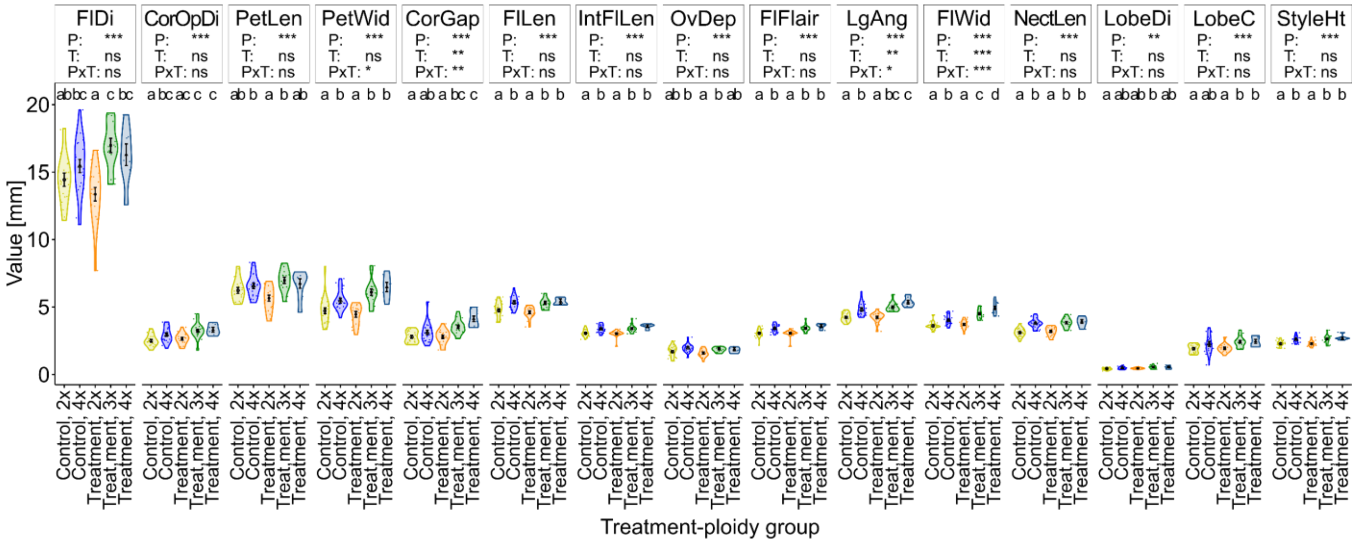

Fig. S15. Differences in individual floral morphology traits (for full names, see Fig. 2) among cytotypes (2x: diploid, 3x: triploid, 4x: tetraploid) and between colchicine-treated (Treatment) and untreated (Control) groups in *Lithophragma bolanderi* of the synthetic polyploidization experiment. Violin plots and points representing individuals are color-coded according to treatment and cytotype. Black points and error bars represent means ± 1 standard error as estimated from linear mixed-effect models (LMMs). Different lowercase letters at the top of the graphs indicate significant (P < 0.05) differences in pairwise post hoc comparisons among the five groups of these LMMs. Significance levels of LMMs for cytotype (P), treatment (T), and the cytotype × treatment interaction (PxT) are indicated directly underneath the floral trait names: “ns” P > 0.5, “*” P < 0.05, “**” P < 0.01, “***” P < 0.001.

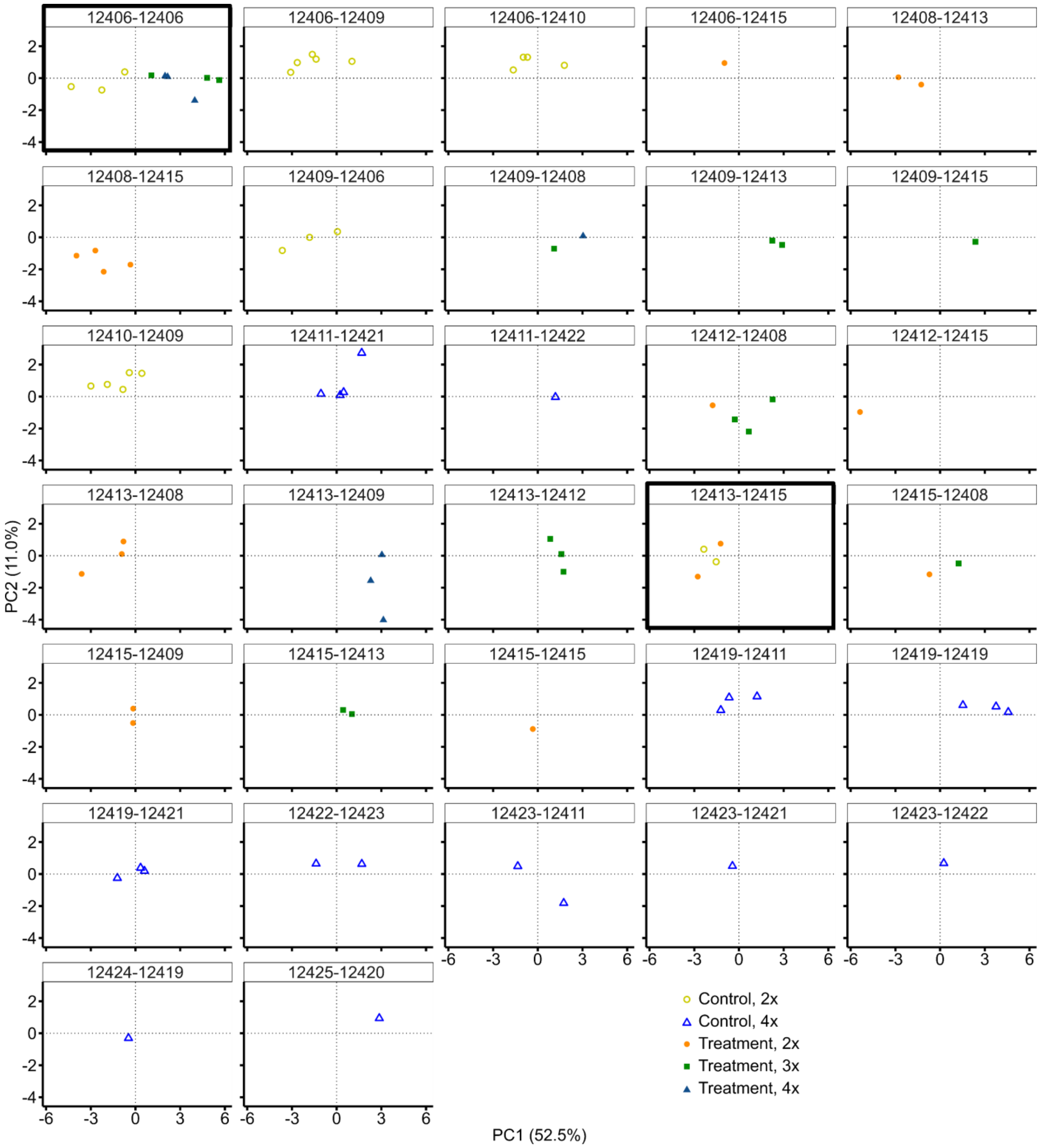

Fig. S16. Differentiation in floral morphology in *Lithophragma bolanderi* among cytotypes (2x: diploid, 3x: triploid, 4x: tetraploid) and between colchicine-treated (Treatment) and untreated (Control) groups according to principal component 1 (PC1) and PC2 and separately for each donor-receiver seed family combination (donor seed family-receiver seed family). Symbols represent individuals and are color-coded according to cytotype and shaped according to treatment. The variance explained by the PCs is given in brackets. Traits loading on the PCs are shown in Fig. 3A (see also SI Appendix, Table S12). Crossings of parents with the same seed family and with at least two of the five groups with at least two individuals per group (framed with a thick line) were used for cross-specific analyses to control for founder effects. In one parental seed family, which comprised natural diploids, neotetraploids, and neotriploids, average (PERMANOVA; SI Appendix, Fig. S17) but not the variance in (PERMDISP: $F_{2,6}$ = 1.25, P = 0.47) multivariate floral morphology differed among cytotypes, and cytotype explained 63.1% of the total variation in floral morphology (PERMANOVA; SI Appendix, Fig. S17). In another parental seed family, which comprised natural diploids and colchicine-treated plants that remained diploid, there was no significant difference in average multivariate floral morphology among treatments, and treatment only explained 12.3% of the total variation in floral morphology (PERMANOVA; SI Appendix, Fig. S17).

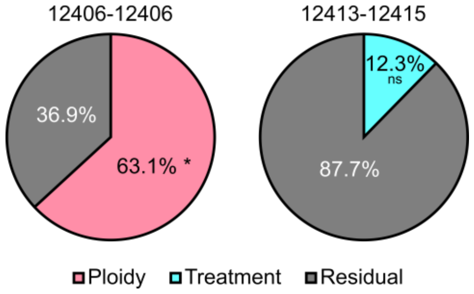

Fig. S17. The percentage of variance in floral morphology explained by the factors cytotype (Ploidy) and treatment (whether plants were treated with colchicine or not) in the crossing lineage 12406-12406 (donor seed family-receiver seed family), which comprised diploid control plants, neotetraploids, and neotriploids and in the crossing lineage 12413-12415, which comprised diploid control plants and colchicine-treated plants that remained diploid, respectively, of the synthetic polyploidization experiment estimated from permutational analyses of variance (PERMANOVAs). Significance levels for these factors are indicated: "^{ns}" $P > 0.5$, "*" $P < 0.05$, "**" $P < 0.01$, "***" $P < 0.001$.

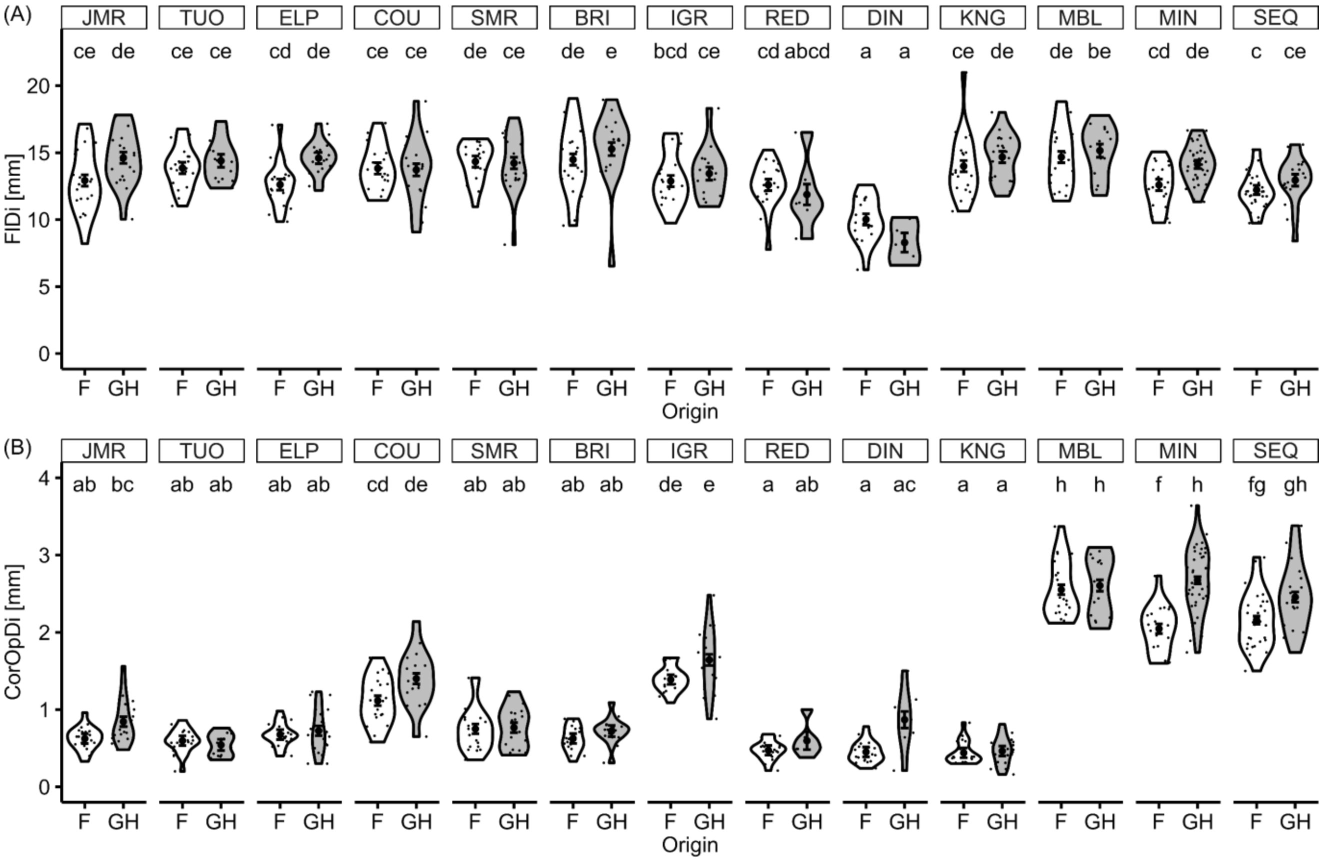

Fig. S18. Comparability of flower morphology between *Lithophragma bolanderi* plants in natural populations (F) and plants grown in the greenhouse (GH) assessed for (**A**) flower diameter (FlDi) and (**B**) corolla-opening diameter (CorOpDi) for 13 populations (for geographical details and full names of the populations, see Fig. 1A and SI Appendix, Table S1). The population are ordered according to latitude from north (left) to south (right). When possible, the second fully open flower from the top of a stalk of each plant when it was fully flowering was selected, and flower diameter and corolla-opening diameter was measured to the nearest 10 µm using a digital caliper. Flowers of plants in the natural population were measured in spring 2017 and sample sizes were as follows: n_{JMR} = 20, n_{TUO} = 20, n_{COU} = 20, n_{ELP} = 20, n_{SMR} = 20, n_{BRI} = 20, n_{IGR} = 20, n_{RED} = 20, n_{DIN} = 20, n_{KNG} = 20, n_{MBL} = 20, n_{MIN} = 20, n_{SEQ} = 30. For the measures of flowers of greenhouse-grown plants, we used a subset of the individuals grown in the common garden descending from these populations. In particular, we selected one individual per seed family, when possible, the individual for which we also measured ploidy level, otherwise a randomly selected individual. This resulted in the following sample sizes: n_{JMR} = 20, n_{TUO} = 15, n_{COU} = 17, n_{ELP} = 20, n_{SMR} = 19, n_{BRI} = 15, n_{IGR} = 15, n_{RED} = 6, n_{DIN} = 7, n_{KGN} = 20, n_{MBL} = 15, n_{MIN} = 37, n_{SEQ} = 18. Small black points represent individuals and violin plots are color-coded according to plant origin (i.e. whether plants were growing in the field (F) or grown in the greenhouse (GH)). Larger black points and error bars represent means ± 1 standard error. The extent of the differences between greenhouse-grown plants and plants in natural populations differed among populations both for flower diameter (linear model (LM): $F_{12,468}$ = 2.06, P = 0.018) and corolla-opening diameter (LM: $F_{12,468}$ = 4.70, P < 0.001). In addition, both traits differed among populations (LM; FlDi: $F_{12,468}$ = 15.51, P < 0.001; CorOpDi: $F_{12,468}$ = 288.32, P < 0.001) and between greenhouse-grown plants and plants in natural populations (LM; FlDi: $F_{1,468}$ = 7.21, P = 0.007; CorOpDi: $F_{1,468}$ = 46.26, P < 0.001). For these analyses, an LM for each trait as response variable was run with plant origin, population, and the plant origin × population interaction as fixed effects. However, pairwise post hoc comparisons indicated that neither flower diameter nor corolla-opening diameter significantly differed in any population except for the population MIN where the corolla-opening diameter was larger in greenhouse-grown plants than in plants growing in the natural population. Different lowercase letters at the top of the graphs indicate significant (P < 0.05) differences in pairwise post hoc comparisons from an LM with population-plant origin group as factor.

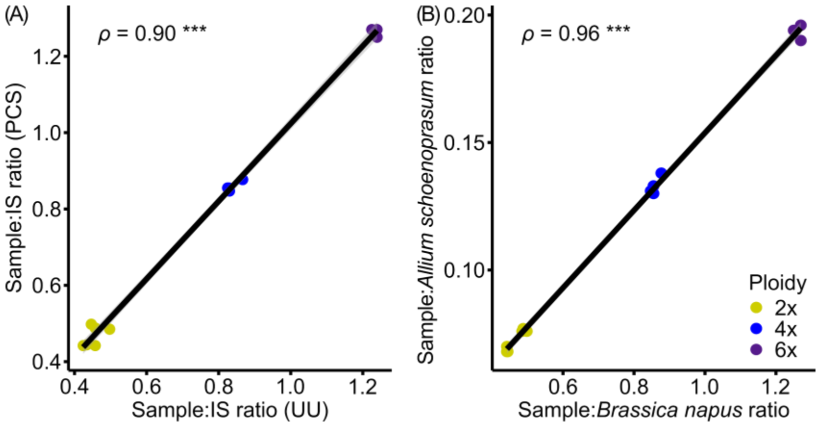

Fig. S19. Comparability of the assessment of the ploidy level of *Lithophragma bolanderi* individuals from fresh leaf material using two different flow cytometry protocols. (**A**) The comparability of the ploidy level assessed on different flow cytometers/by different persons was assessed by correlating the mean ratios of the sample peak and the internal-standard peak (sample:IS ratios) quantified by the authors on a BD LSRFortessa^{TM} flow cytometer at BIOVIS (Biolgical Visualisation) at Uppsala University, Sweden (UU; https://biovis.uu.se/) with the sample:IS ratios quantified by Plant Cytometry Service (PCS; https://www.plantcytometry.nl/). *Brassica napus* was used as internal standard (IS). (**B**) The comparability of the ploidy level assessed using different IS was assessed by correlating the sample:IS ratios of samples with *B. napus* as internal standard (Sample:*Brassica napus* ratio) with the sample:IS ratios of samples with *Allium schoenoprasum* as IS (Sample:*Allium schoenoprasum* ratio). All these samples were analysed by Plant Cytometry Service. In both plots, the samples are colored according to cytotype (Ploidy; 2x: diploid, 4x: tetraploid, 6x: hexaploid). The grey shadings indicate the 95% confidence intervals. Spearman's rank correlations ρ and the significance levels ("***": $P < 0.001$) are given.

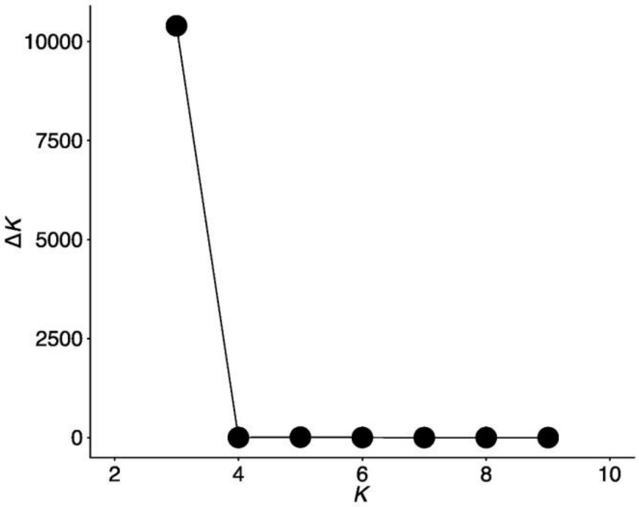

Fig. S20. ΔK across the numbers of *K* run, indicating that $K = 3$ (highest value of ΔK) was the most likely number of the STRUCTURE analysis.

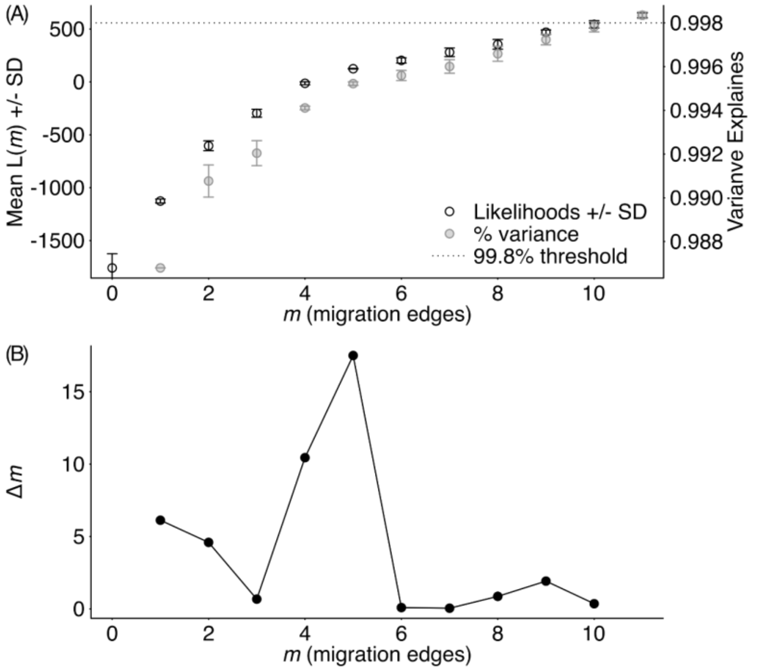

Fig. S21. A total of 30 iterations were run for each possible number of migration edges and for *m* 1-10. (**A)** The mean and the standard deviation (SD) for the composite likelihood L(m) (left axis, black circles) and proportion of variance explained (right axis, grey circles). The 99.8% threshold is reached at *m* = 10. According to (35), a model that explains 99.8% of variation in the relatedness between populations is sufficiently robust to infer the number of migration edges. **(B)** The second-order rate of change (Δ*m*) across values of *m*. The peak is at *m* = 5 edges indicating that five migrations are most likely.

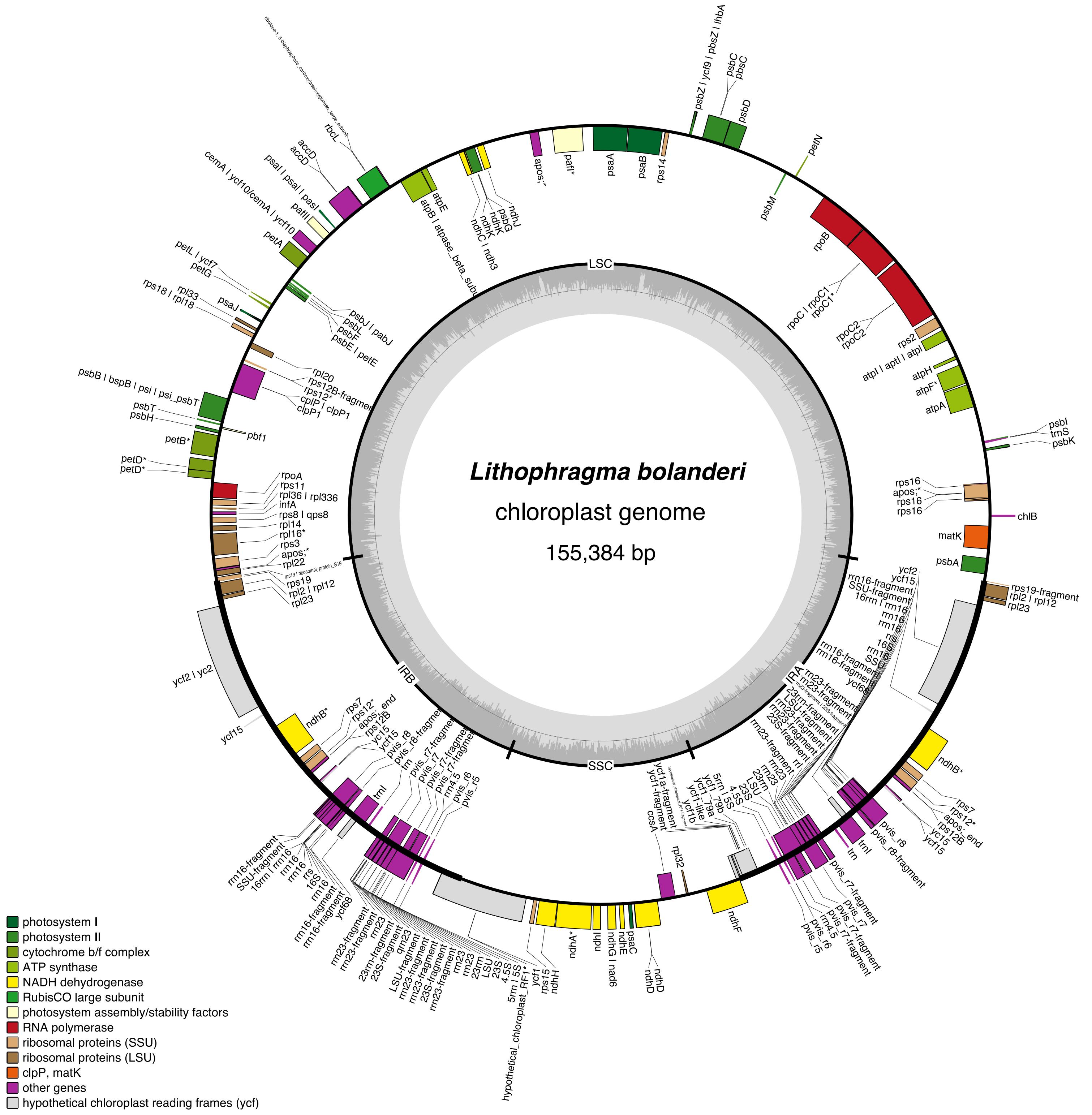

Fig. S22 Complete chloroplast genome of *Lithophragma bolanderi*.

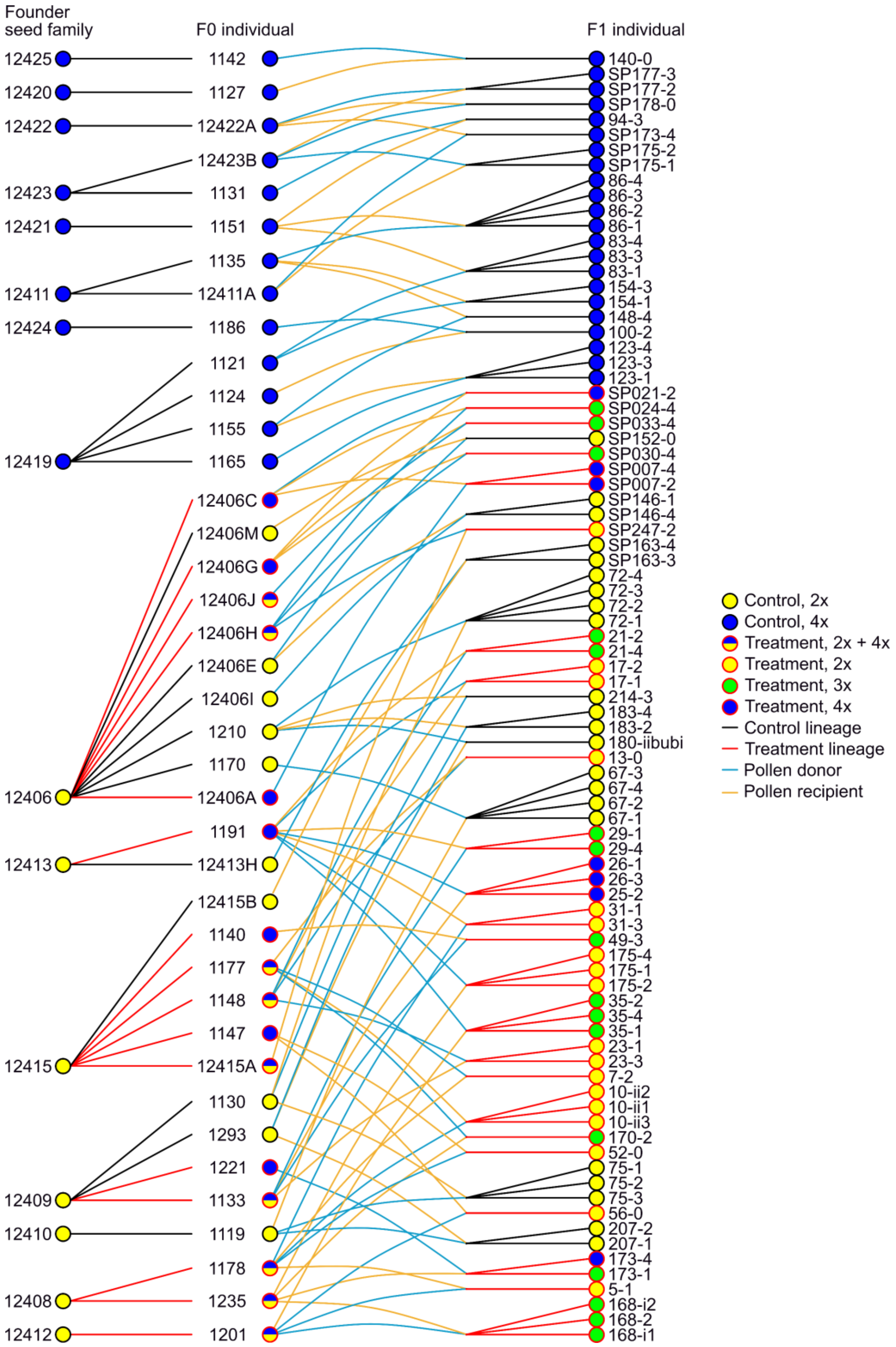

Fig. S23. Sample sizes and genealogy of *Lithophragma bolanderi* plants included in the synthetic polyploidization experiments. Only the F1 individuals for which floral morphology could be measured as well as the identity (F0 individuals) and seed family (Founder seed family) of their parental plants are shown. Each circle represents an individual, and they are color-coded according to cytotype (2x: diploid, 3x: triploid, 4x: tetraploid, 2x + 4x: chimeric plant with 2x and 4x nuclei). Straight lines and borders of the circles are color-coded according to whether the plants belonged to the control groups (i.e. those for which the F0 plants were not treated with colchicine) or the treatment groups (i.e. those for which the F0 plants were treated with colchicine). Curved lines are color-coded according to whether the F0 individuals served as pollen donor (father) and/or as pollen recipient (mother). Note that for some F0 individuals of the treatment plants, flow cytometry analyses indicated that they were tetraploid, but they must have been chimeric plants as (some of) the F1 plants resulting from them were 3x.

Table S1. List of all populations from where plants originally descended for each of the *Lithophragma* species included in the study (BOL = *L. bolanderi*, CYM = *L. cymbalaria*, GLA = *L. glabrum*, HET = *L. heterophyllum*). The population abbreviation, their geographic coordinates, their elevation, and the *Greya* moth present (POL = *G. politella*, OBS = *G. obscura*) are given. Sample sizes for ploidy level analyses from the common garden and from the field, exploratory phylogenetic analysis, whole-genome resequencing, and floral morphology for the common garden and for the synthetic polyploidization experiment are listed.

							Number of individuals (number of seed families)									
							Ploidy level analysis						Floral morphology			
							Common garden			Field †	Phylogenetic analysis (entire ITS sequence / ITS2 Sequence)	Whole-genome resequencing	Common garden ‡			Synthetic polyploidization experiment §
Species	Population	Population abbreviation	Latitude [°N]	Longitude [°E]	Elevation [m a.s.l.]	*Greya* moth community *	2016/2017	2017/2018	Total	2019			2016/2017	2017/2018	Total	
BOL	Oregon Creek	ORE	39.397	-121.077	507			2 (2)	2 (2)							
BOL	Hopland	HOP	39.000	-123.074	297		2 (2)		2 (2)		1 (1) / 1 (1)	1 (1)				
BOL	South Fork American River	SAM	38.795	-120.775	405	POL		21 (20)	21 (20)		1 (1) / 2 (2)	1 (1)		55 (20)	55 (20)	
BOL	Electra	ELE	38.326	-120.683	236	POL+OBS		20 (20)	20 (20)		0 (0) / 1 (1)			39 (19)	39 (19)	
BOL	Jesus Maria Road	JMR	38.286	-120.659	300	POL	20 (20)		20 (20)		0 (0) / 2 (2)				71 (20)	
BOL	New Hogan Lake	NHL	38.199	-120.722	227	POL+OBS		21 (20)	21 (20)		0 (0) / 1 (1)	1 (1)		37 (16)	37 (16)	
BOL	Douglas Flat	DOF	38.110	-120.481	627					21						
BOL	Bear Creek	BEA	38.108	-120.661	321					21						
BOL	Sixmile Creek	SMC	38.096	-120.504	525					19						
BOL	Sneak Gulch	SNE	38.041	-120.457	329					37						
BOL	Bald Mountain	BAM	38.006	-120.350	836	POL		19 (19)	19 (19)		0 (0) / 1 (1)	4 (4)		46 (19)	46 (19)	
BOL	Tuolumne	TUO	37.990	-120.201	730	POL	15 (15)		15 (15)		0 (0) / 2 (2)		74 (15)		74 (15)	
BOL	El Portal	ELP	37.669	-119.838	487	POL+OBS	20 (20)		20 (20)		1 (1) / 2 (2)		56 (20)		56 (20)	
BOL	Coulterville	COU	37.661	-120.145	676	POL+OBS	19 (19)		19 (19)				76 (17)		76 (17)	
BOL	South Fork Merced River	SMR	37.649	-119.883	463	POL+OBS	20 (20)		20 (20)		1 (1) / 1 (1)		84 (19)		84 (19)	
BOL	Briceburg	BRI	37.607	-119.965	363	POL+OBS	15 (15)		15 (15)		1 (1) / 1 (1)		70 (15)		70 (15)	
BOL	Indian Gulch Road	IGR	37.427	-120.165	241	POL+OBS	15 (15)		15 (15)		0 (0) / 1 (1)		60 (15)		60 (15)	
BOL	Lillian Mountain	LIL	37.206	-119.778	355		3 (3)	17 (17)	20 (20)		1 (1) / 2 (2)		15 (3)	45 (17)	60 (20)	
BOL	Kerkhoff Lake	KER	37.148	-119.505	314					41						
BOL	Redinger Lake	RED	37.131	-119.395	927	POL+OBS	6 (6)	2 (2)	8 (7)		0 (0) / 2 (2)		11 (5)	1 (1)	12 (6)	
BOL	Dinkey Creek	DIN	36.989	-119.125	1297	POL	6 (6)	4 (4)	10 (8)		1 (1) / 2 (2)		16 (6)	3 (2)	19 (7)	
BOL	Cats Head Mountain	CHM	36.965	-119.216	655	POL		12 (12)	12 (12)		0 (0) / 2 (2)			34 (12)	34 (12)	
BOL	Watts Valley Road	WVR	36.960	-119.348	592	POL		24 (20)	24 (20)		0 (0) / 1 (1)	1 (1)		53 (20)	53 (20)	
BOL	Maxon Road	MXN	36.917	-119.316	429	POL+OBS		44 (20)	44 (20)		0 (0) / 2 (2)	5 (5)		41 (20)	41 (20)	
BOL	Eagle Peak	EPA	36.914	-119.273	304					23						
BOL	Hog Mountain	HOM	36.885	-119.296	364					16						
BOL	King's River	KNG	36.858	-119.104	336	POL+OBS	11 (11)	26 (9)	37 (20)		0 (0) / 1 (1)	1 (1)	44 (11)	20 (9)	64 (20)	
BOL	Hughes Mountain	HUG	36.856	-119.358	304			21 (20)	21 (20)		1 (1) / 1 (1)			54 (20)	54 (20)	
BOL	Wonder Valley	WON	36.799	-119.321	195					20						
BOL	Ellis Mountain	ELL	36.539	-119.011	329			20 (20)	20 (20)		0 (0) / 1 (1)			48 (20)	48 (20)	
BOL	Paradise Creek	PAC	36.514	-118.760	980					22						
BOL	Marble Falls Trails, Sequoia NP	MBL	36.522	-118.799	686	POL	16 (16)		16 (16)		0 (0) / 1 (1)	1 (1)	50 (16)		50 (16)	
BOL	North Fork Kaweah River	NFK	36.521	-118.894	477					20						
BOL	Kaweah	KAW	36.484	-118.920	345	POL		52 (20)	52 (20)		1 (1) / 2 (2)			49 (19)	49 (19)	85 (46 (15))
BOL	Mineral King Road, Sequoia	MIN	36.459	-118.829	673	POL	98 (20)	73 (18)	171 (38)		0 (0) / 3 (3)	5 (5)	90 (20)	66 (17)	156 (37)	
BOL	Three Rivers	TRV	36.411	-118.894	300	POL		20 (20)	20 (20)		2 (2) / 2 (2)			46 (20)	46 (20)	
BOL	South Fork Kaweah River Road, Sequoia	SEQ	36.351	-118.769	1080	POL		51 (20)	51 (20)		1 (1) / 2 (2)			41 (18)	41 (18)	
BOL	Battle Mountain	BAT	36.263	-118.762	996	POL		28 (13)	28 (13)		1 (1) / 2 (2)	2 (2)		13 (10)	13 (10)	
BOL	Yokohl Road, above Yokohl Valley	YOK	36.231	-118.864	655					19						
BOL	Woody	WOO	35.720	-118.798	740	POL	33 (11)	10 (9)	43 (20)		0 (0) / 1 (1)		24 (10)	25 (9)	49 (19)	
CYM	Santa Cruz Island, Prisoner's Harbor	SCP	34.020	-119.689	62						4 (4) / 0 (0)					
GLA	Portola	POR	39.815	-120.404	1495							4 (4)				
HET	Quail Ridge	QRG	38.494	-122.150	166						1 (1) / 0 (0)					
Total							299 (199)	487 (305)	786 (501)	259	18 (18) / 42 (42)	26 (26)	741 (192)	716 (288)	1457 (479)	85 (46 (15))

* The information on the *Greya* moth community of the populations that we included in the analyses of the floral morphology traits was extracted from SI Appendix, Table S1 in Friberg et al. (50) (the 12 populations: JMR, TUO, COU, SMR, IGR, RED, DIN, KNG, MBL, MIN, SEQ, WOO) or was based on field observations conducted as described in Thompson et al. (51) (the remaining 12 populations).

† As leaf-material samples were collected in the field, only the number of individuals analyzed are given and the number of seed families cannot be provided.

‡ For the four mixed-ploidy populations MXN, KAW, MIN, and BAT, the sample sizes differentiated by cytotypes were as follows. $MXN_{2017/2018}$ / MXN_{Total}: n_{2x} = 14 (9), n_{4x} = 27 (12); $KAW_{2017/2018}$ / KAW_{Total}: n_{2x} = 27 (9), n_{4x} = 22 (10); MIN: $n_{2016/2017, 2x}$ = 38 (9), $n_{2016/2017, 4x}$ = 52 (11), $n_{2017/2018, 2x}$ = 41 (11), $n_{2017/2018, 4x}$ = 25 (6), $n_{Total, 2x}$ = 79 (20), $n_{Total, 4x}$ = 77 (17); $BAT_{2017/2018}$ / BAT_{Total}: n_{4x} = 9 (8), n_{6x} = 4 (4). For all other populations, all individuals were of the same cytotype (for details, see Fig. 2B and/or SI Appendix, Fig. S10).

§ The number in brackets indicates the number of seed families (i.e., the number of different crosses) of the 85 F1, and the number in brackets within the brackets indicates the number of seed families of the parental plants.

Table S2: Sequences of the four loci with the six positions with fixed differences between diploid *Lithophragma bolanderi* and *L. glabrum* and their occurrence in percent. These positions with the fixed species-specific differences are highlighted in bold, and three positions up- and downstream thereof are shown. All polyploid *L. bolanderi* individuals were heterozygous at these positions shown as the main sequence (Polyploid) and the sub-sequence (Polyploid (sub-seq.)), and, in general, exhibited both *L. glabrum* and *L. bolanderi* specific ITS variants at each locus. At locus 2, all polyploids possessed the *L. bolanderi* specific ITS variant in one of the three positions. Sample sizes: $n_{\text{diploid } L.\ bolanderi}$ = 6, $n_{\text{polyploid } L.\ bolanderi}$ = 7 (5 tetraploids, 2 hexaploids), $n_{\text{diploid } L.\ glabrum}$ = 3 (extracted from Kuzoff et al. (8)).

Locus	Position	Species	Ploidy level	Sequence	Occurrence
1	37	*L. bolanderi*	Diploid	CGT**G**GTC	100%
		L. bolanderi	Polyploid (sub-seq.)	CGT**G**GTC	86% [*]
		L. bolanderi	Polyploid	CGT**T**GTC	100%
		L. glabrum	Diploid	CGT**T**GTC	100%
2	93/97/99	*L. bolanderi*	Diploid	AGG**C**GTT**A**T**T**GGG	100%/100%/100%
		L. bolanderi	Polyploid (sub-seq.)	AGG**C**GTT**A**T**T**GGG	57%/43%/71% [†]
		L. bolanderi	Polyploid	AGG**T**GTT**A**T**C**GGG	100%/100%/100%
		L. glabrum	Diploid	AGG**T**GTT**G**T**C**GGG	100%/100%/100%
3	456	*L. bolanderi*	Diploid	GGG**A**CAA	100%
		L. bolanderi	Polyploid (sub-seq.)	GGG**A**CAA	100%
		L. bolanderi	Polyploid	GGG**G**CAA	100%
		L. glabrum	Diploid	GGG**G**CAA	100%
4	654	*L. bolanderi*	Diploid	GCT**T**TTG	100%
		L. bolanderi	Polyploid (sub-seq.)	GCT**T**TTG	100%
		L. bolanderi	Polyploid	GCT**A**TTG	100%
		L. glabrum	Diploid	GCT**A**TTG	100%

[*] 14% (1 sample) had an A in position 37.
[†] 43% (3 samples), 57% (4 samples), and 29% (2 samples) had a G, a T, and a G in position 93, 97, and 99, respectively.

Table S3: Mapping percentages of diploid, tetraploid, and hexaploid *Lithophragma bolanderi* individuals as well as of *L. glabrum* individuals on the *L. bolanderi* reference genome. Samples are ordered according to species, ploidy level (Ploidy), and populations from north to south. For details on populations see, SI Appendix, Table S1.

Species	Ploidy	Population	Plant ID	Sample code	% Primary mapped *
L. bolanderi	2x	BAM	13198A	P18758_101	97.39
			13184A	P18758_102	97.39
			13180A	P18758_103	97.43
			13192C	P18758_104	97.40
		MXN	12715A	P18758_161	97.42
			12707C	P18758_162	97.39
			12708C	P18758_163	97.47
			12711A	P18758_164	97.37
		MIN	11483C	P18758_131	97.37
			11484B	P18758_132	97.14
			11479C	P18758_133	97.31
			11478E	P18758_134	97.24
	4x	WVR	12731B	P18758_174	97.29
		MXN	12714A	P18758_170	97.19
		KNG	11544A	P18758_169	97.19
		MBL	8857A	P18758_172	97.22
		MIN	11453D	P18758_171	97.41
		BAT	13308A	P18758_173	97.33
	6x	HOP	966A	P18758_178	97.46
		SAM	13220A	P18758_176	97.33
		NHL	12853B	P18758_175	97.26
		BAT	13299B	P18758_177	97.38
L. glabrum	2x	POR	1	P18758_201	94.82
			2	P18758_202	95.13
			5	P18758_203	94.60
			6	P18758_204	93.39

* Primary mapped: Reads that are properly matched to the reference genome and are counted only once, even if they could match in several places. Primary means the read is the main or best match, not an extra one.

Table S4. Summary of all nuclear genome analyses conducted and their prediction for allopolyploid and autopolyploid origins of polyploids. The predictions supported by the results of our analyses are highlighted in grey. Note that all these analyses support an autopolyploid origin. Figures and/or tables where the results are presented are also listed.

Analysis	Allopolyploidy	Autopolyploidy	Result
Mapping rates	Lower mapping rates for polyploid samples compared to *L. bolanderi* diploids	Similar mapping rates of *L. bolanderi* polyploids and diploids	SI Appendix, Table S3
K-mer	The k-mer spectrum plot has aaab < aabb	The k-mer spectrum plot has aaab > aabb	Fig. 1D; SI Appendix, Table S5
Allele frequency spectra	Increase of alleles at intermediate frequency in polyploids but not diploids	No peak at intermediate allele frequencies in polyploids	SI Appendix, Fig. S4
L. bolanderi allele proportion	Most polyploids would have both parental alleles (peak around 50%)	Most polyploids share allele with one of the two putative parental taxa	SI Appendix, Fig. S5
Principal component analysis	Polyploids have intermediate loadings on the first PC separating the two parental taxa	Polyploids clustering with one of the putative parental taxa	Fig. 1B
Clustering analysis (STRUCTURE)	A substantial likelihood of polyploids but not diploids to belong to the cluster of the parental taxa putatively giving rise to allopolyploidization at the best value of K	Polyploids cluster mainly with one of two putative parental taxa at the best value of K	Fig. 1C
TreeMix	Strong migration edges from the species suggested to give rise to allopolyploidization to polyploids but not diploids	No strong migration edges leading to polyploids but not diploids	SI Appendix, Fig. S6
GRAMPA	Best trees include both trees where the polyploids cluster with one and trees where they cluster with the other putative parental species, whereas diploids only cluster with one parental species	Best trees where polyploids cluster with diploids of one of the two putative parental taxa	SI Appendix, Fig. S7

Table S5. GenomeScope2 k-mer analysis output (k-mer = 21) on tetraploid and hexaploid *Lithophragma bolanderi* individuals. Note that aaab>aabb, which is reported only for tetraploids, indicates autopolyploidy.

Sample	Population	Plant ID	Ploidy	Estimated genome length [Mb]	aaab%	aabb%	K-mer coverage
P18758_169	KNG	11544A	4x	470.4	3.38	1.84	13
P18758_170	MXN	12714A	4x	454.8	3.32	1.79	13.7
P18758_171	MIN	11453D	4x	465.3	3.12	1.69	14
P18758_172	MBL	8857A	4x	464.2	3.3	1.8	16.9
P18758_173	BAT	13308A	4x	466.1	3.2	1.77	14.8
P18758_174	WVR	12731B	4x	475.4	3.16	1.73	15.9
P18758_175	NHL	12853B	6x	441	NA	NA	14.9
P18758_176	SAM	13220A	6x	461	NA	NA	12.5
P18758_177	BAT	13299B	6x	461	NA	NA	12.5
P18758_178	HOP	966A	6x	431.7	NA	NA	12.8

Table S6: Eigenvalues, percentage of variance explained, and cumulative percentage of variance explained by the first 25 principal components (PCs) resulting from a principal component analysis (PCA) on 21,667 single nucleotide polymorphisms (SNPs) (all non-coding SNPs) genome wide. PC1 and PC2 (framed with a thick line) were used to produce the PCA plot shown in Fig. 1B.

PC	Eigenvalue	% variance	Cumulative % variance
1	213.96	20.20	20.20
2	140.34	13.25	33.46
3	88.97	8.40	41.86
4	56.41	5.33	47.18
5	42.02	3.97	51.15
6	40.91	3.86	55.01
7	37.11	3.50	58.52
8	36.12	3.41	61.93
9	35.77	3.38	65.31
10	34.10	3.22	68.53
11	33.59	3.17	71.70
12	33.21	3.14	74.83
13	32.48	3.07	77.90
14	30.47	2.88	80.78
15	25.78	2.43	83.21
16	24.86	2.35	85.56
17	24.81	2.34	87.90
18	23.60	2.23	90.13
19	22.96	2.17	92.30
20	20.37	1.92	94.22
21	17.95	1.69	95.92
22	16.41	1.55	97.47
23	16.02	1.51	98.98
24	5.55	0.52	99.51
25	5.23	0.49	100.00

Table S7: Pairwise Spearman's rank correlations among multiple measures of the same trait (for details on traits, see Fig. 2A). Significance levels are indicated: "***": $P < 0.001$.

Trait	Measure 1	Measure 2	ρ
Petal length (PetLen)	PetLen1	PetLen2	0.851***
Petal width (PetWid)	PetWid1	PetWid2	0.878***
Long floral angle (LgAng)	LgAng1	LgAng2	0.883***
Outer distance between lobes (LobeC)	LobeC1	LobeC2	0.874***
	LobeC1	LobeC3	0.865***
	LobeC1	LobeC4	0.675***
	LobeC2	LobeC3	0.852***
	LobeC2	LobeC4	0.777***
	LobeC3	LobeC4	0.762***

Table S8: Eigenvalues, percentage of variance explained, and cumulative percentage of variance explained by the principal components (PCs) resulting from a principal component analysis on the nine morphology traits (for full names, see Fig. 2) measured on *Lithophragma bolanderi* flowers from the greenhouse common-garden, and the correlations between the floral morphology traits and the PCs. Significant correlations are highlighted in bold and significance levels are indicated: "" $P > 0.5$, "*" $P < 0.05$, "**" $P < 0.01$, "***" $P < 0.001$. Significant positive correlations are shaded in blue and significant negative correlations in red. PC1 and PC2 (framed with a thick line) were indicated as significant PCs by the broken stick criterion indicated PC1 and PC2 as significant PCs and were used for further considerations.

Trait	PC1	PC2	PC3	PC4	PC5	PC6	PC7	PC8	PC9
Eigenvalue	3.60	1.99	0.85	0.80	0.59	0.40	0.29	0.27	0.22
% variance	39.95	22.07	9.41	8.86	6.56	4.40	3.23	3.04	2.49
Cumulative % variance	39.95	62.02	71.42	80.28	86.84	91.25	94.47	97.51	100.00
CorOpDi	**0.638** ***	**-0.626** ***	**0.134** ***	**-0.147** ***	**0.099** ***	-0.028	**0.123** ***	**0.218** ***	**0.299** ***
PetLen	**0.194** ***	**0.772** ***	**0.417** ***	**0.299** ***	0.024	**0.178** ***	**0.106** ***	**-0.137** ***	**0.203** ***
PetWid	**0.697** ***	**-0.133** ***	**0.576** ***	**0.242** ***	0.006	**-0.199** ***	-0.007	**0.111** ***	**-0.235** ***
CorGap	**0.756** ***	**-0.107** ***	-0.013	**-0.291** ***	**0.458** ***	**0.282** ***	**0.054** *	**-0.148** ***	**-0.136** ***
IntFlLen	**0.309** ***	**0.822** ***	**-0.053** *	**-0.251** ***	**0.130** ***	-0.001	**-0.253** ***	**0.287** ***	0.017
OvDep	**0.583** ***	0.037	**-0.496** ***	**0.602** ***	**0.056** *	**0.122** ***	**0.084** **	**0.151** ***	-0.044
FlWid	**0.849** ***	-0.044	**-0.179** ***	**0.108** ***	**0.060** *	**-0.294** ***	**-0.248** ***	**-0.258** ***	**0.122** ***
LobeDi	**0.734** ***	**-0.184** ***	0.038	**-0.117** ***	**-0.545** ***	**0.308** ***	**-0.141** ***	-0.021	-0.020
StyleHt	**0.632** ***	**0.507** ***	**-0.202** ***	**-0.304** ***	**-0.223** ***	**-0.219** ***	**0.330** ***	-0.032	-0.051

Table S9: Differences in multivariate dispersion of floral morphology in *Lithophragma bolanderi* Significant *P*-values are highlighted in bold. Different lowercase letters indicate significant (*P* < 0.05) differences in pairwise comparisons of cytotypes.

	Mean ± SE			Statistics		
Measure	2x	4x	6x	*F*	df1,df2	*P*
Multivariate dispersion	2.40 ± 0.03 [a]	2.88 ± 0.04 [c]	2.57 ± 0.06 [b]	43.86	2,1454	**< 0.001**

Table S10: Statistical output of linear mixed-effect models (LMMs) assessing differences in floral morphology traits (for full names, see Fig. 2) and principal component (PC) scores for PC1 and PC2 among cytotypes and among the three diploid-tetraploid mixed populations MIN, KAW, and MXN (for full names, see SI Appendix, Table S1) in *Lithophragma bolanderi*. Significant *P*-values are highlighted in bold.

	Ploidy		Population		Ploidy × population	
Trait	χ^2_1	*P*	χ^2_2	*P*	χ^2_2	*P*
FlDi	3.52	0.060	18.20	**< 0.001**	4.56	0.102
CorOpDi	1.93	0.165	630.53	**< 0.001**	10.46	**0.005**
PetLen	8.86	**0.003**	73.52	**< 0.001**	17.73	**< 0.001**
PetWid	1.35	0.245	27.06	**< 0.001**	6.23	**0.044**
CorGap	3.17	0.075	27.52	**< 0.001**	2.03	0.362
FlLen	33.64	**< 0.001**	19.71	**< 0.001**	1.02	0.601
IntFlLen	10.15	**0.001**	44.65	**< 0.001**	8.31	**0.016**
OvDep	33.07	**< 0.001**	2.94	0.230	14.45	**0.001**
FlFlair	16.13	**< 0.001**	31.17	**< 0.001**	9.06	**0.011**
LgAng	27.66	**< 0.001**	1.94	0.378	3.26	0.196
FlWid	17.66	**< 0.001**	45.38	**< 0.001**	2.87	0.238
NectLen	25.92	**< 0.001**	67.22	**< 0.001**	4.15	0.125
LobeDi	2.76	0.097	1.15	0.562	5.07	0.079
LobeC	1.51	0.219	2.76	0.251	4.13	0.127
StyleHt	30.02	**< 0.001**	29.70	**< 0.001**	6.58	**0.037**
PC1	24.21	**< 0.001**	22.55	**< 0.001**	5.48	0.065
PC2	28.83	**< 0.001**	245.93	**< 0.001**	24.18	**< 0.001**

Table S11: Differences in multivariate dispersion of floral morphology in *Lithophragma bolanderi* in the three populations KAW, MIN, and MXN with diploid (2x) and tetraploid (4x) plants and the population BAT with tetraploid and hexaploid (6x) plants (for details on population, see Fig. 1A and SI Appendix, Table S1) based on permutational analyses of multivariate dispersion (PERMDISP) for each mixed-ploidy population. Significant *P*-values are highlighted in bold.

	Mean ± SE			Statistics		
Population	2x	4x	6x	*F*	df1,df2	*P*
KAW	1.89 ± 0.19	2.30 ± 0.14		2.67	1,47	0.137
MIN	2.25 ± 0.08	2.13 ± 0.07		1.19	1,154	0.292
MXN	1.98 ± 0.12	2.43 ± 0.13		5.11	1,39	**0.037**
BAT		2.10 ± 0.18	3.29 ± 0.55	7.02	1,11	**0.021**

Table S12: Eigenvalues, percentage of variance explained, and cumulative percentage of variance explained by the principal components (PCs) resulting from a principal component analysis on the nine morphology traits (for full names, see Fig. 2) measured on *Lithophragma bolanderi* flowers from synthetic polyploidization experiment, and the correlations between the floral morphology traits and the PCs. Significant correlations are highlighted in bold and significance levels are indicated: "" *P* > 0.5, "*" *P* < 0.05, "**" *P* < 0.01, "***" *P* < 0,001. Significant positive correlations are shaded in blue and significant negative correlations in red. The broken stick criterion indicated PC1 as the only significant PC. For consistency with the analyses of the common garden plants and for visualization purposes, the first two PCs (framed with a thick line) and not only PC1 were used for further considerations.

Trait	PC1	PC2	PC3	PC4	PC5	PC6	PC7	PC8	PC9
Eigenvalue	4.72	0.99	0.94	0.70	0.59	0.38	0.31	0.21	0.15
% variance	52.48	11.04	10.46	7.77	6.60	4.25	3.42	2.31	1.69
Cumulative % variance	52.48	63.52	73.97	81.74	88.34	92.59	96.00	98.31	100.00
CorOpDi	**0.741** ***	**-0.256** *	**-0.322** **	0.117	**0.264** *	**0.413** ***	-0.099	0.131	0.04
PetLen	**0.725** ***	**0.426** ***	0.043	**-0.291** **	**0.339** **	-0.144	0.122	0.209	-0.108
PetWid	**0.845** ***	0.151	-0.058	**-0.269** *	0.227 *	-0.056	-0.16	**-0.304** **	0.121
CorGap	**0.796** ***	-0.195	**-0.288** **	**0.262** *	-0.053	**-0.264** *	**0.26** *	0.042	0.186
IntFlLen	**0.806** ***	0.116	0.045	-0.059	**-0.424** ***	-0.102	**-0.331** **	0.17	0.05
OvDep	**0.505** ***	**0.63** ***	0.163	**0.537** ***	-0.06	0.141	0.044	-0.085	-0.034
FlWid	**0.834** ***	**-0.392** ***	-0.099	0.161	-0.027	-0.141	-0.045	-0.109	**-0.285** **
LobeDi	**0.377** ***	**-0.344** **	**0.822** ***	0.131	0.197	-0.026	-0.024	0.041	0.068
StyleHt	**0.747** ***	-0.064	0.187	**-0.354** ***	**-0.363** ***	**0.26** *	**0.274** *	-0.058	-0.017

Table S13. Mean coverage per individual for genomic sites used in this study after filtering for minimum and maximum coverage for diploid (2x), tetraploid (4x), and hexaploid (6x) *Lithophragma bolanderi* (BOL) as well as for diploid *L. glabrum* (GLA), which was identified as a potential parental lineage to polyploid *L. bolanderi* by Kuzoff et al. (8). The file names on NCBI (Fastq_file_ID) are also given.

Species	Cytotype	Population	Individual ID	Fastq file ID	Mean coverage (X)
BOL	2x	BAM	13198A	P18758_101	20.0943
			13184A	P18758_102	20.2138
			13180A	P18758_103	20.2858
			13192C	P18758_104	22.0861
		MIN	11483C	P18758_131	27.5902
			11484B	P18758_132	18.6105
			11479C	P18758_133	29.3068
			11478E	P18758_134	28.1548
		MXN	12715A	P18758_161	26.551
			12707C	P18758_162	31.8876
			12708C	P18758_163	30.821
			12711A	P18758_164	21.7614
	4x	BAT	13308A	P18758_173	50.9031
		KNG	11544A	P18758_169	44.0064
		MBL	8857A	P18758_172	56.546
		MIN	11453D	P18758_171	47.8851
		MXN	12714A	P18758_170	46.1582
		WVR	12731B	P18758_174	53.5608
	6x	BAT	13299B	P18758_177	54.7706
		HOP	966A	P18758_178	67.6427
		NHL	12853B	P18758_175	64.2888
		SAM	13220A	P18758_176	57.3986
GLA	2x	POR	GLA1	P18758_201	24.4548
			GLA2	P18758_202	36.7517
			GLA5	P18758_203	32.5888
			GLA6	P18758_204	28.3407

Table S14. Detailed list of the model structure of the models used to analyze differences in individual floral morphology traits and principal components (PCs) among the three major cytotypes – diploids, tetraploids, and hexaploids – of *Lithophragma bolanderi* flowers from the greenhouse common-garden. Type II sum of squares were used for models without interactions in the fixed effects and type III sum of squares for models with interactions.

Question	Model type	Response variable	Fixed effect	Random effects
Did the 15 numeric floral morphology traits differ among cytotypes?				
	LMM	Numeric floral morphology trait *	Cytotype	Cytotype | Population / Seed family<br>1 | Year when plants were grown
Did the categorical trait shape of the petal edge differ among cytotypes?				
	GLMM †	Petal edge shape ‡	Cytotype	Cytotype | Population / Seed family<br>1 | Year when plants were grown
Did PC scores differ among cytotypes?				
	LMM	PC score §	Cytotype	Cytotype | Population / Seed family<br>1 | Year when plants were grown

GLMM: generalized linear mixed-effect model
LMM: linear mixed-effect model
* An LMM for each of the 15 numerical floral morphology traits: FlDi, CorOpDi, PetLen, PetWid, CorGap, FlLen, IntFlLen, OvDep, FlFlair, LgAng, FlWid, NectLen, LobeDi, LobeC, and StyleHt (for full names, see Fig. 2).
† With a binominal error distribution and logit link function.
‡ Petal edge shape was categorized as either whole (0) or lobed (1) (see Fig. 2B).
§ An LMM each for PC1 and PC2.

Table S15. Detailed list of the model structure of the models used to analyze differences in multivariate floral morphology among the three major cytotypes – diploids, tetraploids, and hexaploids – of *Lithophragma bolanderi* flowers from the greenhouse common-garden. All models were computed with the number of permutations set to 9999. Type II sum of squares were used for models without interactions in the fixed effects and type III sum of squares for models with interactions.

Question	Model type	Response variables	Fixed effect	Random effect(s)
Did multivariate floral morphology differ among cytotypes and what was the relative contribution of cytotype to multivariate variation?				
	PERMANOVA	Euclidean distance matrix *	Cytotype	Cytotype / Population<br>Cytotype / Population / Seed Family<br>Year when plants were grown
Did multivariate variation in floral morphology differ among cytotypes?				
	PERMDISP	Euclidean distance matrix *	Cytotype	---

PERMANOVA: permutational multivariate analysis of variance
PERMDISP: permutational analysis of multivariate dispersion
---: not applicable
* Euclidean distances were calculated based on normalized trait values.

Table S16. Detailed list of the model structure of the models used to analyze relationships among floral morphology, cytotype, and geography in multivariate for the three major cytotypes – diploids, tetraploids, and hexaploids – of *Lithophragma bolanderi* flowers from the greenhouse common-garden. All models were computed with the number of permutations set to 9999.

Question	Model type	Distance matrix 1	Distance matrix 2	Distance matrix 3
Was floral morphology more similar among populations of the same cytotype than among populations of different cytotypes while controlling for geographical distance among populations?				
	Partial Mantel test	Morphological distance *	Same vs. different cytotype †	Geographical distance ‡
Was floral morphology more similar among populations in closer geographic distance while controlling for cytotype?				
	Partial Mantel test	Morphological distance *	Geographical distance ‡	Same vs. different cytotype †

* Euclidean distances were calculated based on normalized population mean floral trait values estimated from linear mixed-effect models (see SI Appendix, Table S17) using the function *distance* in the R package *ecodist* (52).

† A matrix containing the information whether populations were of the same (0) or of a different (1) cytotype was computed using the function *bcdist* in the R package *ecodist* (52).

‡ Geographical distances among populations in kilometers were computed using the function *geodist* in the R package *geodist* (53).

Table S17. Detailed list of the model structure of the models used to estimate population means of the 15 continuously varying floral morphological traits and the principal component (PC) scores for PC1 and PC2 separately for the three major cytotypes – diploids, tetraploids, and hexaploids – of *Lithophragma bolanderi* from the greenhouse common-garden. For all models, we used type II sum of squares.

Question	Model type	Response variable	Fixed effect	Random effects
What were the population means of the floral morphological traits?				
	LMM	Numeric floral morphology trait *	Cytotype-population group	1 | Seed family<br>1 | Year when plants were grown
What were the population means of the PC scores?				
	LMM	PC †	Cytotype-population group	1 | Seed family<br>1 | Year when plants were grown

LMM: linear mixed-effect model

* An LMM for each of the 15 numerical floral morphology traits: FlDi, CorOpDi, PetLen, PetWid, CorGap, FlLen, IntFlLen, OvDep, FlFlair, LgAng, FlWid, NectLen, LobeDi, LobeC, and StyleHt (for full names, see Fig. 2).

† An LMM each for PC1 and PC2

Table S18. Detailed list of the model structure of the models used to analyze differences in univariate and multivariate floral morphology among *Lithophragma bolanderi* cytotypes in the three diploid-tetraploid mixed-ploidy populations KAW, MIN, and MXN and the tetraploid-hexaploid mixed-ploidy population BAT part of greenhouse common-garden. For details on the populations, see Fig. 1A and SI Appendix, Table S1. Type II sum of squares were used for models without interactions in the fixed effects and type III sum of squares for models with interactions. All PERMANOVAs and PERMDISPs were computed with the number of permutations set to 9999.

Question	Model type	Response variables	Fixed effect(s)	Random effect(s)
Did the 15 numeric floral morphology traits differ between diploids and tetraploids in the mixed-ploidy population KAW?				
	LMM	Numeric floral morphology trait *	Cytotype	Cytotype | Seed family
Did the 15 numeric floral morphology traits differ between diploids and tetraploids in the mixed-ploidy population MIN?				
	LMM	Numeric floral morphology trait *	Cytotype	Cytotype | Seed family<br>1 | Year when plants were grown
Did the 15 numeric floral morphology traits differ between diploids and tetraploids in the mixed-ploidy population MXN?				
	LMM	Numeric floral morphology trait *	Cytotype	Cytotype | Seed family
Did the 15 numeric floral morphology traits differ between tetraploids and hexaploids in the mixed-ploidy population BAT?				
	LMM	Numeric floral morphology trait *	Cytotype	1 | Seed family
Did differences in the 15 numeric floral morphology traits between diploids and tetraploids differ among the three diploid-tetraploid mixed-ploidy population KAW, MIN, and MXN?				
	LMM	Numeric floral morphology trait *	Cytotype<br>Population<br>Cytotype × Population	Cytotype | Seed family<br>1 | Year when plants were grown
Did PC scores differ between diploids and tetraploids in the mixed-ploidy population KAW?				
	LMM	PC scores †	Cytotype	Cytotype | Seed family
Did PC scores differ between diploids and tetraploids in the mixed-ploidy population MIN?				
	LMM	PC scores †	Cytotype	Cytotype | Seed family<br>1 | Year when plants were grown
Did PC scores differ between diploids and tetraploids in the mixed-ploidy population MXN?				
	LMM	PC scores †	Cytotype	Cytotype | Seed family
Did PC scores differ between tetraploids and hexaploids in the mixed-ploidy population BAT?				
	LMM	PC scores †	Cytotype	1 | Seed family
Did differences in the PC scores between diploids and tetraploids differ among the three diploid-tetraploid mixed-ploidy population KAW, MIN, and MXN?				
	LMM	PC scores †	Cytotype<br>Population<br>Cytotype × Population	Cytotype | Seed family<br>1 | Year when plants were grown
Did multivariate floral morphology differ between diploids and tetraploids and what was the relative contribution of cytotype to multivariate variation in the mixed-ploidy population KAW?				
	PERMANOVA	Euclidean distance matrix ‡	Cytotype	Cytotype / Seed Family
Did multivariate floral morphology differ between diploids and tetraploids and what was the relative contribution of cytotype to multivariate variation in the mixed-ploidy population MIN?				
	PERMANOVA	Euclidean distance matrix ‡	Cytotype	Cytotype / Seed Family<br>Cytotype / Year when plants were grown
Did multivariate floral morphology differ between diploids and tetraploids and what was the relative contribution of cytotype to multivariate variation in the mixed-ploidy population MXN?				
	PERMANOVA	Euclidean distance matrix ‡	Cytotype	Cytotype / Seed Family
Did multivariate floral morphology differ between diploids and tetraploids and what was the relative contribution of cytotype to multivariate variation in the mixed-ploidy population BAT?				
	PERMANOVA	Euclidean distance matrix ‡	Cytotype	Cytotype / Seed Family
Did multivariate variation in floral morphology differ between diploids and tetraploids in the mixed-ploidy population KAW?				
	PERMDISP	Euclidean distance matrix ‡	Cytotype	---
Did multivariate variation in floral morphology differ between diploids and tetraploids in the mixed-ploidy population MIN?				
	PERMDISP	Euclidean distance matrix ‡	Cytotype	---
Did multivariate variation in floral morphology differ between diploids and tetraploids in the mixed-ploidy population MXN?				
	PERMDISP	Euclidean distance matrix ‡	Cytotype	---
Did multivariate variation in floral morphology differ between tetraploids and hexaploids in the mixed-ploidy population BAT?				
	PERMDISP	Euclidean distance matrix ‡	Cytotype	---

LMM: linear mixed-effect model
PC: principal component
PERMANOVA: permutational multivariate analysis of variance
PERMDISP: permutational analysis of multivariate dispersion
---: not applicable
* An LMM for each of the 15 numerical floral morphology traits: FlDi, CorOpDi, PetLen, PetWid, CorGap, FlLen, IntFlLen, OvDep, FlFlair, LgAng, FlWid, NectLen, LobeDi, LobeC, and StyleHt (for full names, see Fig. 2).
† An LMM each for PC1 and PC2.
‡ Euclidean distances were calculated based on normalized trait values of the nine numerical floral morphology traits with all pairwise Spearman's rank correlation coefficients < 0.7 (see SI Appendix, Fig. S9): CorOpDi, PetLen, PetWid, CorGap, IntFlLen, OvDep, FlWid, LobeDi, and StyleHt (for full names, see Fig. 2).

Table S19. Detailed list of the model structure of the models used to analyze differences univariate and multivariate floral morphology of *Lithophragma bolanderi* among the three major cytotypes and between the two different *Greya* moth communities based on population means of traits (computed using linear mixed-effect models as detailed in SI Appendix, Table S17). Type II sum of squares were used for models without interactions in the fixed effects and type III sum of squares for models with interactions. All PERMANOVAs and PERMDISPs were computed with the number of permutations set to 9999.

Question	Model type	Response variables	Fixed effect(s)
Did the 15 numeric floral morphology traits differ among cytotypes and/or Greya *moth communities?*			
	LM	Numeric floral morphology trait *	Cytotype<br>Moth<br>Cytotype × Moth<br>Latitude ¶
Did the 15 numeric floral morphology traits differ among the six groups? †			
	LM	Numeric floral morphology trait *	Group<br>Latitude ¶
Did PC scores differ among cytotypes and/or Greya *moth communities?*			
	LM	PC scores ‡	Cytotype<br>Moth<br>Cytotype × Moth<br>Latitude ¶
Did PC scores differ among the six groups? †			
	LM	PC scores ‡	Group<br>Latitude ¶
Did multivariate floral morphology differ among cytotypes and/or Greya *moth communities and what was the relative contribution of cytotype and* Greya *moth communities to multivariate variation?*			
	PERMANOVA	Euclidean distance matrix §	Cytotype<br>Moth<br>Cytotype × Moth Latitude ¶
Did multivariate variation in floral morphology differ among the six groups?			
	PERMDISP	Euclidean distance matrix §	Group

LM: linear model (more specifically a two-way analysis of variance (ANOVA))
PC: principal component
PERMANOVA: permutational multivariate analysis of variance
PERMDISP: permutational analysis of multivariate dispersion
Cytotype: The three major cytotypes were included: diploids (2x), tetraploids (4x), and hexaploids (6x).
Moth: There were two *Greya* moth communities: populations with *G. politella* only and populations with *G. politella* and *G. obscura*.
Group: There were the following six groups: diploid populations with *G. politella* only (2x, POL), diploid populations with both *Greya* species (2x, POL+OBS), tetraploid populations with *G. politella* only (4x, POL), tetraploid populations with both *Greya* species (4x, POL+OBS), hexaploid populations with *G. politella* only (6x, POL), hexaploid populations with both *Greya* species (6x, POL+OBS).
---: not applicable
* An LM for each of the 15 numerical floral morphology traits: FlDi, CorOpDi, PetLen, PetWid, CorGap, FlLen, IntFlLen, OvDep, FlFlair, LgAng, FlWid, NectLen, LobeDi, LobeC, and StyleHt (for full names, see Fig. 2).
† This analysis was used to compute all pairwise post hoc comparisons among the six groups.
‡ An LM each for PC1 and PC2.
§ Euclidean distances were calculated based on normalized trait values of the nine numerical floral morphology traits with all pairwise Spearman's rank correlation coefficients < 0.7 (see SI Appendix, Fig. S9): CorOpDi, PetLen, PetWid, CorGap, IntFlLen, OvDep, FlWid, LobeDi, and StyleHt (for full names, see Fig. 2).
¶ Latitude was included as a covariate in the model to account for the potential for spatial autocorrelation.

Table S20. Detailed list of the model structure of the models used to analyze differences in univariate and multivariate floral morphology of *Lithophragma bolanderi* among neopolyploids, diploids and established tetraploids descending from the population KWA, the diploid-tetraploid mixed-ploidy population with the most pronounced differences between diploids and tetraploids in the greenhouse common-garden (see Results; for details on the populations, see Fig. 1A and SI Appendix, Table S1). Neopolyploids were generated by synthetically inducing polyploidization in diploid seedlings using colchicine. Type II sum of squares were used for models without interactions in the fixed effects and type III sum of squares for models with interactions. All PERMANOVAs and PERMDISPs were computed with the number of permutations set to 9999.

Question	Model type	Response variables	Fixed effect(s)	Random effect(s)
Did the 15 numeric floral morphology traits differ among cytotypes and/or treatments?				
	LMM	Numeric floral morphology trait [*]	Cytotype<br>Treatment<br>Cytotype × Treatment	Original cytotype | Donor-receiver seed family
Did the 15 numeric floral morphology traits differ among the five groups? [†]				
	LMM	Numeric floral morphology trait [*]	Group	Original cytotype | Donor-receiver seed family
Did PC scores differ among cytotypes and/or treatments?				
	LMM	PC scores [‡]	Cytotype<br>Treatment<br>Cytotype × Treatment	Original cytotype | Donor-receiver seed family
Did PC scores differ among the five groups? [†]				
	LMM	PC scores [‡]	Group	Original cytotype | Donor-receiver seed family
Did multivariate floral morphology differ among cytotypes and/or treatments and what was the relative contribution of cytotype and treatment to multivariate variation?				
	PERMANOVA	Euclidean distance matrix [§]	Cytotype<br>Treatment<br>Cytotype × Treatment	Original cytotype / Donor-receiver seed family
Did multivariate floral morphology differ among the five groups? [†]				
	PERMANOVA	Euclidean distance matrix [§]	Group	Original cytotype / Donor-receiver seed family
Did multivariate floral morphology differ among the five groups? [¶]				
	PERMANOVA	Euclidean distance matrix [§]	Group	Treatment-ploidy group / Donor-receiver seed family
Did multivariate variation in floral morphology differ among the five groups?				
	PERMDISP	Euclidean distance matrix [§]	Group	---
Did multivariate floral morphology differ among cytotypes and what was the relative contribution of cytotype to multivariate variation in the donor-receiver seed family 12406-12406?				
	PERMANOVA	Euclidean distance matrix [§]	Cytotype	---
Did multivariate variation in floral morphology differ among cytotypes in the donor-receiver seed family 12406-12406?				
	PERMDISP	Euclidean distance matrix [§]	Cytotype	---
Did multivariate floral morphology differ between treatments and what was the relative contribution of treatment to multivariate variation in the donor-receiver seed family 12413-12415?				
	PERMANOVA	Euclidean distance matrix [§]	Treatment	---
Did multivariate variation in floral morphology differ between treatments in the donor-receiver seed family 12413-12415?				
	PERMDISP	Euclidean distance matrix [§]	Treatment	---

Donor-receiver seed family: The cross-specific combination of the seed family of the pollen donor and the pollen receiver.
LMM: linear mixed-effect model
Original cytotype: The cytotype of the founder plants of the five different groups, which was diploid for the groups “control, 2x”, “treatment, 2x”, “treatment, 3x”, and “treatment, 4x” and tetraploid for the group “control, 4x” (see “group”).
PC: principal component
PERMANOVA: permutational multivariate analysis of variance
PERMDISP: permutational analysis of multivariate dispersion
Treatment: There were two treatments. One treatment consisted of the plants that were colchicine-treated to synthetically induce polyploidization, and the other treatment consisted of the control plants, i.e. the plants that were not treated with colchicine.
Group: There were the following five groups: control diploids (control, 2x), control tetraploids (control, 4x), colchicine-treated plants that remained diploid (treatment, 2x), neotriploids (treatment, 3x; most likely resulting from a crossing involving at least one ploidy chimeric parental plant), and neotetraploids (treatment, 4x).
---: not applicable
[*] An LMM for each of the 15 numerical floral morphology traits: FlDi, CorOpDi, PetLen, PetWid, CorGap, FlLen, IntFlLen, OvDep, FlFlair, LgAng, FlWid, NectLen, LobeDi, LobeC, and StyleHt (for full names, see Fig. 2).
[†] This analysis was used to compute all pairwise post hoc comparisons among the five groups.
[‡] An LMM each for PC1 and PC2.
[§] Euclidean distances were calculated based on normalized trait values.
[¶] This analysis was only carried out to get the statistics for the pairwise post hoc comparison between treatment, 2x and treatment, 4x, because data for the plant individuals available did not allow for such a comparison with the model structure of the analysis just above where the donor-receiver seed family was nested in original cytotype.

SI References

1. K. Gross, M. Undin, J. N. Thompson, M. Friberg, Components of local adaptation and divergence in pollination efficacy in a coevolving species interaction. *Ecology* **104**, e4043 (2023).

2. M. Baranyi, J. Greilhuber, Flow cytometric analysis of genom size variation in cultivated and wild *Pisum sativum* (*Fabaceae*). *Plant Syst. Evol.* **194**, 231–239 (1995).

3. J. Doležel, J. Greilhuber, J. Suda, Estimation of nuclear DNA content in plants using flow cytometry. *Nature Protoc.* **2**, 2233–2244 (2007).

4. A. V. Roberts, The use of bead beating to prepare suspensions of nuclei for flow cytometry from fresh leaves, herbarium leaves, petals and pollen. *Cytometry Part A* **71A**, 1039–1044 (2007).

5. A. Cousin, K. Heel, W. A. Cowling, A. N. Nelson, An efficient high-throughput flow cytometric method for estimating DNA ploidy level in plants. *Cytometry A* **75A**, 1015–1019 (2009).

6. R. L. Taylor, The genus *Lithophragma* (Saxifragaceae). *University of California Publications in Botany* **37**, 1–122 (1965).

7. C. E. Barta, *et al.*, In situ dark adaptation enhances the efficiency of DNA extraction from mature pin oak (*Quercus palustris*) leaves, facilitating the identification of partial sequences of the 18S rRNA and isoprene synthase (*IspS*) genes. *Plants* **6**, 52 (2017).

8. R. K. Kuzoff, D. E. Soltis, L. Hufford, P. S. Soltis, Phylogenetic relationships within *Lithophragma* (Saxifragaceae): Hybridization, allopolyploidy, and ovary diversification. *Syst. Bot.* **24**, 598–615 (1999).

9. M. Kearse, *et al.*, Geneious Basic: An integrated and extendable desktop software platform for the organization and analysis of sequence data. *Bioinformatics* **28**, 1647–1649 (2012).

10. J. B. Horne, L. van Herwerden, Long-term panmixia in a cosmopolitan Indo-Pacific coral reef fish and a nebulous genetic boundary with its broadly sympatric sister species. *J Evol Biol* **26**, 783–799 (2013).

11. S. F. Ryan, *et al.*, Global invasion history of the agricultural pest butterfly *Pieris rapae* revealed with genomics and citizen science. *PNAS* **116**, 20015–20024 (2019).

12. K. Clark, I. Karsch-Mizrachi, D. J. Lipman, J. Ostell, E. W. Sayers, GenBank. *Nucleic Acids Res* **44**, D67–D72 (2016).

13. F. A. Simão, R. M. Waterhouse, P. Ioannidis, E. V. Kriventseva, E. M. Zdobnov, BUSCO: assessing genome assembly and annotation completeness with single-copy orthologs. *Bioinformatics* **31**, 3210–3212 (2015).

14. A. Smit, R. Hubley, RepeatModeler Open-1.0. Retrieved from http://www.repeatmasker.org. (2008). Deposited 2015 2008.

15. A. Smit, R. Hubley, P. Green, RepeatMasker Open-4.0. Retrieved from http://www.repeatmasker.org. (2013). Deposited 2015 2013.

16. T. Brůna, K. J. Hoff, A. Lomsadze, M. Stanke, M. Borodovsky, BRAKER2: automatic eukaryotic genome annotation with GeneMark-EP+ and AUGUSTUS supported by a protein database. *NAR Genomics and Bioinformatics* **3**, lqaa108 (2021).

17. M. Stanke, M. Diekhans, R. Baertsch, D. Haussler, Using native and syntenically mapped cDNA alignments to improve *de novo* gene finding. *Bioinformatics* **24**, 637–644 (2008).

18. M. Stanke, O. Schöffmann, B. Morgenstern, S. Waack, Gene prediction in eukaryotes with a generalized hidden Markov model that uses hints from external sources. *BMC Bioinformatics* **7**, 62 (2006).

19. L. Gabriel, K. J. Hoff, T. Brůna, M. Borodovsky, M. Stanke, TSEBRA: transcript selector for BRAKER. *BMC Bioinformatics* **22**, 566 (2021).

20. T. Brůna, A. Lomsadze, M. Borodovsky, GeneMark-EP+: eukaryotic gene prediction with self-training in the space of genes and proteins. *NAR Genomics and Bioinformatics* **2**, lqaa026 (2020).

21. B. Buchfink, C. Xie, D. H. Huson, Fast and sensitive protein alignment using DIAMOND. *Nat Methods* **12**, 59–60 (2015).

22. A. Lomsadze, V. Ter-Hovhannisyan, Y. O. Chernoff, M. Borodovsky, Gene identification in novel eukaryotic genomes by self-training algorithm. *Nucleic Acids Research* **33**, 6494–6506 (2005).

23. H. Iwata, O. Gotoh, Benchmarking spliced alignment programs including Spaln2, an extended version of Spaln that incorporates additional species-specific features. *Nucleic Acids Research* **40**, e161 (2012).

24. O. Gotoh, M. Morita, D. R. Nelson, Assessment and refinement of eukaryotic gene structure prediction with gene-structure-aware multiple protein sequence alignment. *BMC Bioinformatics* **15**, 189 (2014).

25. J. Köster, S. Rahmann, Snakemake - a scalable bioinformatics workflow engine. *Bioinformatics* **28**, 2520–2522 (2012).

26. S. Chen, Y. Zhou, Y. Chen, J. Gu, fastp: an ultra-fast all-in-one FASTQ preprocessor. *Bioinformatics* **34**, i884–i890 (2018).

27. H. Li, R. Durbin, Fast and accurate short read alignment with Burrows–Wheeler transform. *Bioinformatics* **25**, 1754–1760 (2009).

28. G. A. Van der Auwera, B. D. O'Connor, *Genomics in the cloud: using Docker, GATK, and WDL in Terra*, 1st Edition (O'Reilly Media, Inc., 2020).

29. M. A. DePristo, *et al.*, A framework for variation discovery and genotyping using next-generation DNA sequencing data. *Nat Genet* **43**, 491–498 (2011).

30. H. Li, A statistical framework for SNP calling, mutation discovery, association mapping and population genetical parameter estimation from sequencing data. *Bioinformatics* **27**, 2987–2993 (2011).

31. J. K. Pritchard, J. K. Pickrell, G. Coop, The genetics of human adaptation: hard sweeps, soft sweeps, and polygenic adaptation. *Current Biology* **20**, R208–R215 (2010).

32. M. Stift, F. Kolář, P. G. Meirmans, STRUCTURE is more robust than other clustering methods in simulated mixed-ploidy populations. *Heredity* **123**, 429–441 (2019).

33. T. Jombart, I. Ahmed, adegenet 1.3-1: new tools for the analysis of genome-wide SNP data. *Bioinformatics* **27**, 3070–3071 (2011).

34. G. Evanno, S. Regnaut, J. Goudet, Detecting the number of clusters of individuals using the software STRUCTURE: a simulation study. *Molecular Ecology* **14**, 2611–2620 (2005).

35. J. K. Pickrell, J. K. Pritchard, Inference of population splits and mixtures from genome-wide allele frequency data. *PLOS Genetics* **8**, e1002967 (2012).

36. J. K. Pickrell, *et al.*, The genetic prehistory of southern Africa. *Nat Commun* **3**, 1143 (2012).

37. R. R. Fitak, *OptM*: estimating the optimal number of migration edges on population trees using *Treemix*. *Biology Methods and Protocols* **6**, bpab017 (2021).

38. G. W. C. Thomas, S. H. Ather, M. W. Hahn, Gene-tree reconciliation with MUL-trees to resolve polyploidy events. *Systematic Biology* **66**, 1007–1018 (2017).

39. M. Martin, *et al.*, WhatsHap: fast and accurate read-based phasing. [Preprint] (2016). Available at: https://www.biorxiv.org/content/10.1101/085050v2 [Accessed 15 January 2025].

40. B. Q. Minh, *et al.*, IQ-TREE 2: New models and efficient methods for phylogenetic inference in the genomic era. *Molecular Biology and Evolution* **37**, 1530–1534 (2020).

41. E. Paradis, K. Schliep, ape 5.0: an environment for modern phylogenetics and evolutionary analyses in R. *Bioinformatics* **35**, 526–528 (2019).

42. M. Tillich, *et al.*, GeSeq – versatile and accurate annotation of organelle genomes. *Nucleic Acids Research* **45**, W6–W11 (2017).

43. P. Danecek, *et al.*, The variant call format and VCFtools. *Bioinformatics* **27**, 2156–2158 (2011).

44. H. J. Bandelt, P. Forster, A. Röhl, Median-joining networks for inferring intraspecific phylogenies. *Molecular Biology and Evolution* **16**, 37–48 (1999).

45. J. W. Leigh, D. Bryant, POPART: full-feature software for haplotype network construction. *Methods in Ecology and Evolution* **6**, 1110–1116 (2015).

46. R. N. Gutenkunst, R. D. Hernandez, S. H. Williamson, C. D. Bustamante, Inferring the joint demographic history of multiple populations from multidimensional SNP frequency data. *PLOS Genetics* **5**, e1000695 (2009).

47. T. R. Ranallo-Benavidez, K. S. Jaron, M. C. Schatz, GenomeScope 2.0 and Smudgeplot for reference-free profiling of polyploid genomes. *Nat Commun* **11**, 1432 (2020).

48. F. E. Harrell Jr., C. Dupont, many others, Hmisc: Harrell Miscellaneous. **R package version 4.4-2** (2020).

49. R Core Team, *R: A language and environment for statistical computing.* (R Foundation for Statistical Computing, 2020).

50. M. Friberg, C. Schwind, P. R. Guimarães, R. A. Raguso, J. N. Thompson, Extreme diversification of floral volatiles within and among species of *Lithophragma* (Saxifragaceae). *PNAS* **116**, 4406–4415 (2019).

51. J. N. Thompson, C. Schwind, M. Friberg, Diversification of trait combinations in coevolving plant and insect lineages. *Am. Nat.* **190**, 171–184 (2017).

52. S. C. Goslee, D. L. Urban, The ecodist package for dissimilarity-based analysis of ecological data. *J. Stat. Softw.* **22**, 1–19 (2007).

53. M. Padgham, M. D. Sumner, geodist: fast, dependency-free geodesic distance calculations. R package version 0.0.7. (2021). Available at: https://CRAN.R-project.org/package=geodist.
